# Fluorinated Twists: A Pathway to a Stable Pd_8_L_16_ Square Antiprism

**DOI:** 10.1021/jacs.5c09573

**Published:** 2025-08-08

**Authors:** Soumalya Bhattacharyya, Stephen P. Argent, Ben S. Pilgrim

**Affiliations:** School of Chemistry, 6123 University of Nottingham, University Park, Nottingham NG7 2RD, U.K.

## Abstract

Within the extensive family of Pd_*n*_L_2*n*_ metal–organic cages, some structures are much easier to access than others. Ligands are generally constructed from planar aromatic linkers. While small structures (Pd_2_L_4_ to Pd_6_L_12_) and large pseudospherical structures (≥Pd_12_L_24_) can be readily obtained from these flat aryl-based ligands, strategies to intermediate-sized structures have remained elusive as the required angle between metal–ligand coordination vectors (90–120°) is hard to construct from the common toolkit of organic molecules. Herein, we report the Pd_8_L_16_ square antiprism as a new addition to the Pd_*n*_L_2*n*_ family, a structure shown as thermodynamically stable in both solution and the solid state, for the first time. The ligand achieves close to the ideal angle of 105.1° needed for the square antiprism through the incorporation of a perfluorobiphenyl backbone. The substantial dihedral twist induced by the fluorines diverges the coordination vectors compared to nonfluorous examples while crucially maintaining ligand rigidity to avoid the formation of mixtures of structures—a strategy we believe will have widespread applications in (metallo)­supramolecular chemistry.

## Graphical abstract

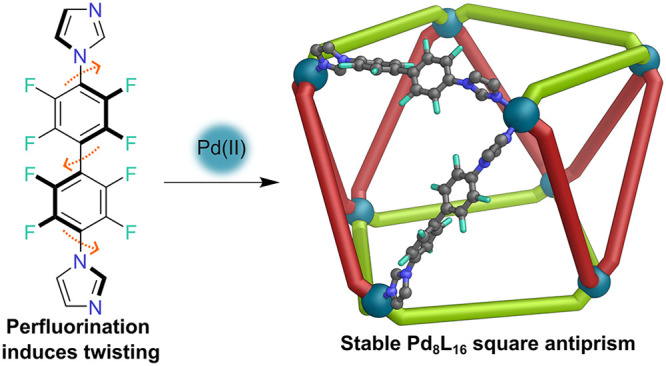

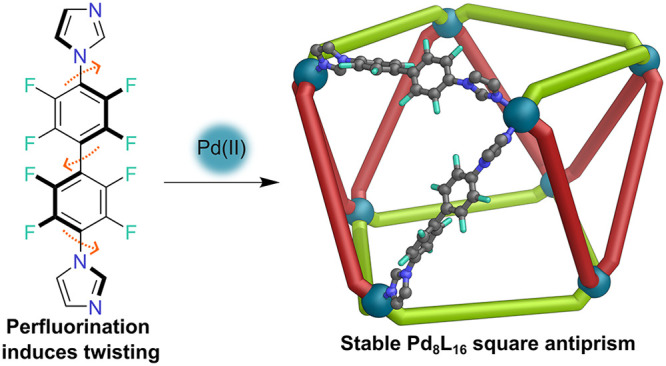

## Introduction

The field of self-assembly looks to construct ever more complex and functional structures which can form from the combination of simple, well-designed pieces in a facile manner. Metal–organic cages, which are held together through dynamic metal–ligand coordination, typically consist of geometrically precise metal nodes linked by rigid organic ligands.^1^ Compared to other supramolecular hosts, metal–organic cages have gained popularity due to their ease of construction, the diverse range of structures that are accessible,^2^ and their well-defined cavities.^3^ Properties under confinement vary substantially from those in the bulk,^4^ and encapsulation of small molecule guests in cage cavities has been employed in areas as diverse as catalysis,^5^ separation science,^6^ drug delivery,^7^ and sensing.^8^

One of the most widely studied metal–organic cage families are Pd_*n*_L_2*n*_ assemblies, which reliably self-assemble from Pd­(II) salts and ditopic monodentate ligands.^9^ A plethora of different assemblies are known, encompassing many common three-dimensional shapes (Figure 1a). Which shape forms is governed primarily by the ligand geometry, specifically the average angle, θ_av_, between the metal–ligand coordination vectors that the ligand possesses in the assembled structure.^10^

**Figure 1:**
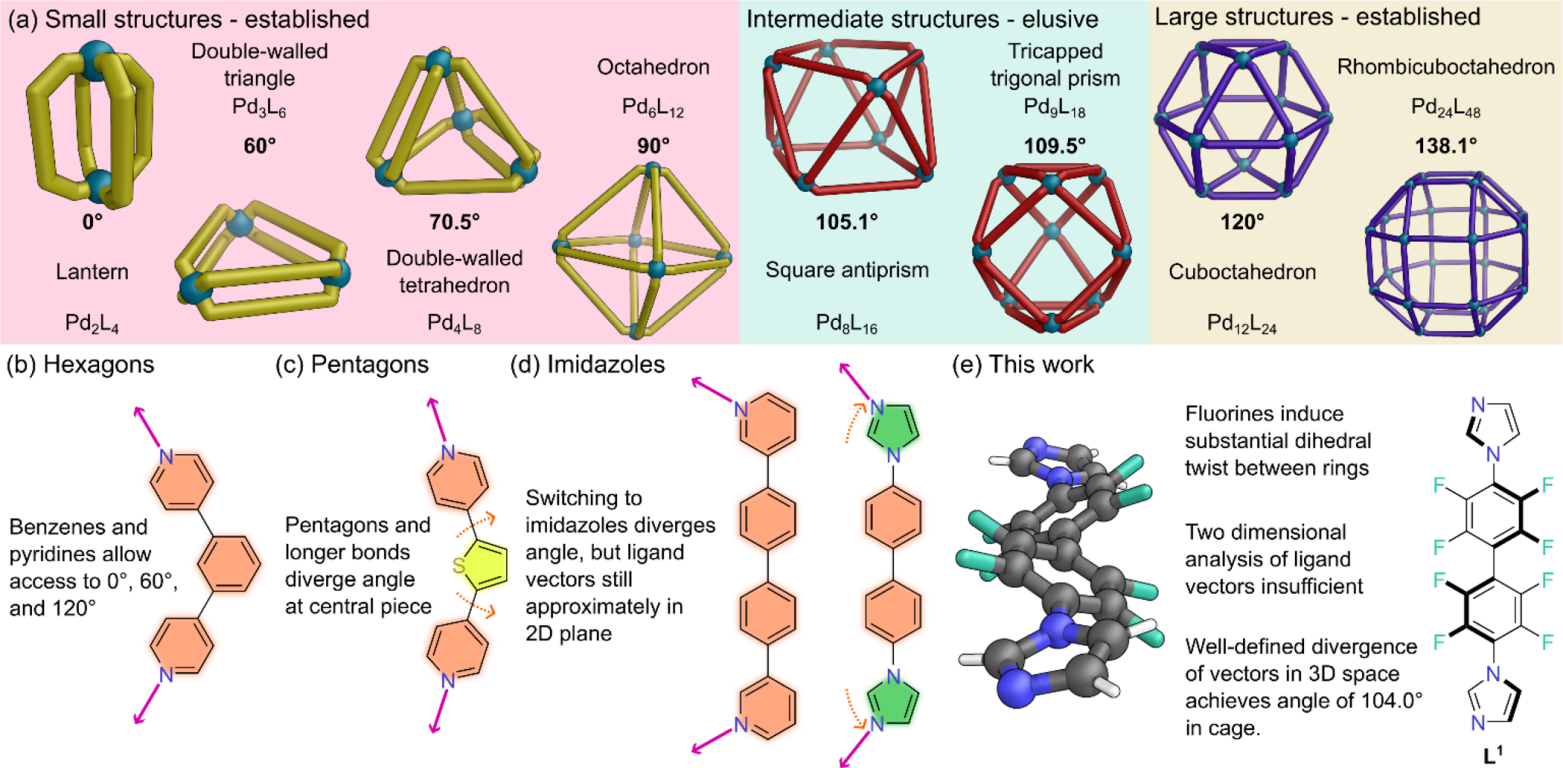
(a) A selection of the possible Pd_*n*_L_2*n*_ metal–organic cage architectures; routes to small structures and to large structures are now well established, but being able to selectively target intermediate-sized structures has proved elusive. (b) Hexagonal pieces allow angles of 0°, 60°, and 120° to be obtained. (c) Central pentagonal pieces diverge angles allowing large structures to be accessed. (d) The use of pentagonal imidazoles as coordinating groups gives access to Pd_6_L_12_ octahedra. (e) In this work, a perfluorobiphenyl linker on ligand **L** ^**1**^ induces a substantial dihedral twist. With the ligand now no longer planar, an angle of 104.0°, close to the ideal 105.1° for a Pd_8_L_16_ square antiprism, is readily obtained. Color: C = gray, *N* = blue, H = white, *F* = aquamarine.

To form an assembly of a particular shape, the ligand must be able to closely match the ideal angle between the coordination planes of the square planar Pd­(II) ions sitting at the vertices of the polyhedra.

In the first generation of assemblies, pyridine nitrogens were the donors of choice, linked by benzene spacers due to their rigidity and planarity. Use of these hexagonal pieces allowed access to Pd_2_L_4_ lanterns (ideal θ = 0°),^11^ Pd_3_L_6_ triangles (θ = 60°),^12^ and Pd_12_L_24_ cuboctahedra (θ = 120°)^13^ (Figure 1b). Occasionally, where ideal angles between structures are close (such as 60° for Pd_3_L_6_ triangles and 70.5° for Pd_4_L_8_ tetrahedra) the same ligand can assemble into more than one type of structure.^14^ Often these mixed assemblies can be directed toward a single product by changing counteranions^15^ or solvents.^16^

Fujita and co-workers pioneered routes to larger structures such as the Pd_24_L_48_ rhombicuboctahedron,^17^ through replacement of the benzene linker with five-membered aromatic heterocycles, as the introduction of the pentagonal piece diverged the coordination vectors beyond 120° (Figure 1c). Even larger structures up to Pd_48_L_96_ Goldberg polyhedra could be accessed through changing the bond lengths in the pentagon (with a selenophene).^18^ Alternatively, Mukherjee and co-workers showed *meta*-substituted, six-membered pyridines could be replaced by *N*-linked imidazoles, allowing vectors to be diverged from 60° to allow formation of Pd_6_L_12_ octahedra (ideal θ = 90°) (Figure 1d).^19^

While construction strategies for small assemblies and large assemblies are now well established, access to intermediate-sized assemblies such as the Pd_8_L_16_ square antiprism (ideal θ = 105.1°) and the Pd_9_L_18_ tricapped trigonal prism (ideal θ = 109.5°) have remained elusive. Fujita and co-workers have reported a single example of a Pd_9_L_18_ structure,^20^ but there has only been limited evidence for a Pd_8_L_16_ species being a metastable intermediate en route to assembly of larger Pd_12_L_24_ cuboctahedra,^20^ although its geometry was not determined.

Structural planning of all these architectures assumes ligand planarity, with the angle between coordination vectors rationalized in a two-dimensional plane. Structures requiring intermediate angles have proved hard to achieve as it has not been possible to construct ligands possessing the requisite angles entirely of aromatic/flat units with continuous sp^2^/sp hybridized atoms. While moving from aryl linkers to nonaromatic linkers could achieve this, the greater flexibility makes mixtures of structures far more likely to form. Instead, imparting a dihedral twist between the aryl rings could allow access to these angles, while not increasing ligand flexibility.

While systems of multiple aryl rings are assumed flat as a first approximation, there is free rotation around biaryl bonds. The dihedral twist or torsion angle, φ, between rings is influenced by multiple factors. The coplanar conformation allows greater electronic delocalization which lowers energy. However, coplanar systems suffer significant steric clashes between the *ortho*-substituents (even if these are hydrogen), which favors twisting. In the solid state, the rings of biphenyl are essentially coplanar (φ ∼ 0°);^21^ more twisted conformations are reported/calculated in solution and in the gas phase.^22^ Regardless of the lowest energy conformation, there is a small energy difference between planar and twisted forms, meaning any twisted conformations are not strictly enforced.

While the addition of many substituents increases the twist angle, we were drawn to perfluorobenzenes for several reasons. Although the difference is not huge, fluorine is larger than hydrogen.^23^ Fluorine’s high electronegativity results in strong polarization in the C–F bond and inversion of the benzene ring quadrupole. These both favor additional twisting, as there is less conjugative stabilization between the rings and twisting also reduces any local electrostatic repulsion between fluorine atoms (as points of negative potential on the surface). This is demonstrated in the solid state structure of decafluorobiphenyl which has a twist, φ = 63.8°.^24^ Decafluorobiphenyl also has the advantage of being commercially available and considerably easier to functionalize through S_N_Ar chemistry than accessing other multiply substituted benzene derivatives through sequential metal coupling reactions.

Our target bisimidazole-substituted biphenyl, **L** ^**1**^, (Figure 1e) possessed a significant dihedral twist as expected, bringing the angle between coordination vectors to 104.0° once coordinated, sufficiently close to the ideal angle of 105.1° to direct assembly into a Pd_8_L_16_ square antiprism. This square antiprism was the exclusive thermodynamically stable architecture made by this system in both the solution state (NMR, DOSY, electrospray ionization mass spectrometry (ESI-MS)) and solid state (single-crystal X-ray diffraction (SCXRD)). The importance of fluorine was confirmed by control experiments with both a nonfluorous ligand and two other sterically demanding ligands producing the Pd_6_L_12_ octahedron. We believe this perfluorobiphenyl linker approach may be of general applicability and provide a straightforward route to access unusual metal–organic architectures.

## Results and Discussion

Ligand **L** ^**1**^ was prepared using a facile S_N_Ar reaction between decafluorobiphenyl and imidazole, with high selectivity for the 4,4’-disubstituted product (Section S4.1 in the Supporting Information). Slow vapor diffusion of hexane into a chloroform solution of **L** ^**1**^ resulted in plate-shaped crystals after 1 week at 22 °C. SCXRD analysis revealed, as expected, that there was a considerable dihedral twist angle, φ, between the aryl rings, with φ = 50.7° between the perfluorobenzene rings and φ = 40.0° and 37.0° between the perfluorobenzene and imidazole rings (Figure 1e and Table S13).

Ligand **L** ^**1**^ and Pd­(NO_3_)_2_ were heated for 3 h at 100 °C in DMSO-*d*_6_, resulting in a clear, pale-yellow solution (Figure 2a). ^1^H and ^19^F NMR spectroscopic studies revealed two sets of ligand signals of equal intensity (Figure 2b and Figures S14, S18–S19). This doubling of signals indicated two different types of ligand environment, something commonly observed with Pd_4_L_8_ double-walled tetrahedra.^25^ However, the ideal θ_av_ for Pd_4_L_8_ tetrahedra is 70.5° and the nitrogen donor atoms on **L** ^**1**^ were too divergent to make this assembly. DOSY NMR spectroscopy indicated a single diffusion coefficient for all protons with a log *D* = – 10.02 (Figure 2C and Figure S16), indicating a single, considerably larger supramolecular assembly had formed. Peaks in the ESI-MS indicated Pd_8_ **L** ^**1**^_16_ metal–organic cage **1** as the predominant species, with well resolved isotopic distribution patterns matching theoretically calculated patterns (Figures S21–S22).

**Figure 2:**
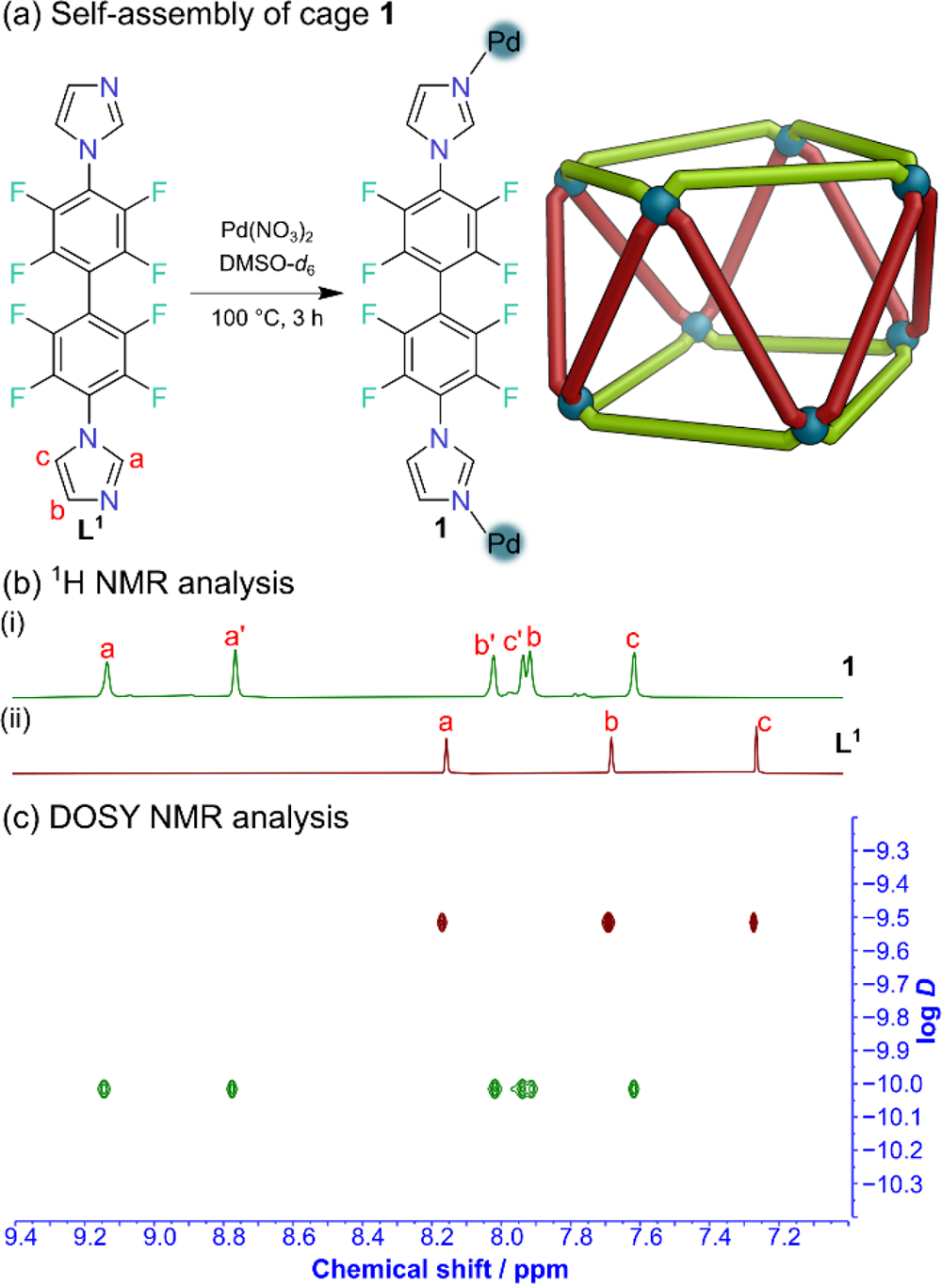
(a) Self-assembly of cage **1**. (b) Stacked ^1^H NMR spectra (DMSO-*d*_*6*_, 500 MHz, 298 K) of: (i) cage **1** (green) and (ii) ligand **L** ^**1**^ (maroon). (c) Overlaid DOSY NMR spectra of cage **1** (green) and ligand **L** ^**1**^ (maroon).

The only thoroughly characterized Pd_8_L_16_ complex is an interlocked catenane of two double-walled squares.^26^ This interlocked catenane has four different types of ligand environment and so can be excluded from the data here. The square antiprism Pd_8_L_16_ structure possesses two types of ligand environment, with ligands that (i) occupy edges between two triangular faces (red), and (ii) occupy edges between a triangular face and a square face (green) (Figure 3d). While Fujita and co-workers reported some evidence for a metastable Pd_8_L_16_ intermediate en route to the assembly of a Pd_12_L_24_ cuboctahedron,^27^ this was the first clear evidence of such a species existing as the thermodynamically favored product of the system. There was no change in the ^1^H NMR spectrum both after 1 week and 3 weeks at 25 °C indicating thermodynamic stability (Figure S44). In the self-assembly of this Pd_8_L_16_ square antiprism, in line with previous studies,^28^ there was also some evidence of smaller metastable intermediates present at shorter time scales (after ∼ 10 min) via ^1^H NMR analysis (Figure S17), but these soon disappeared.

**Figure 3:**
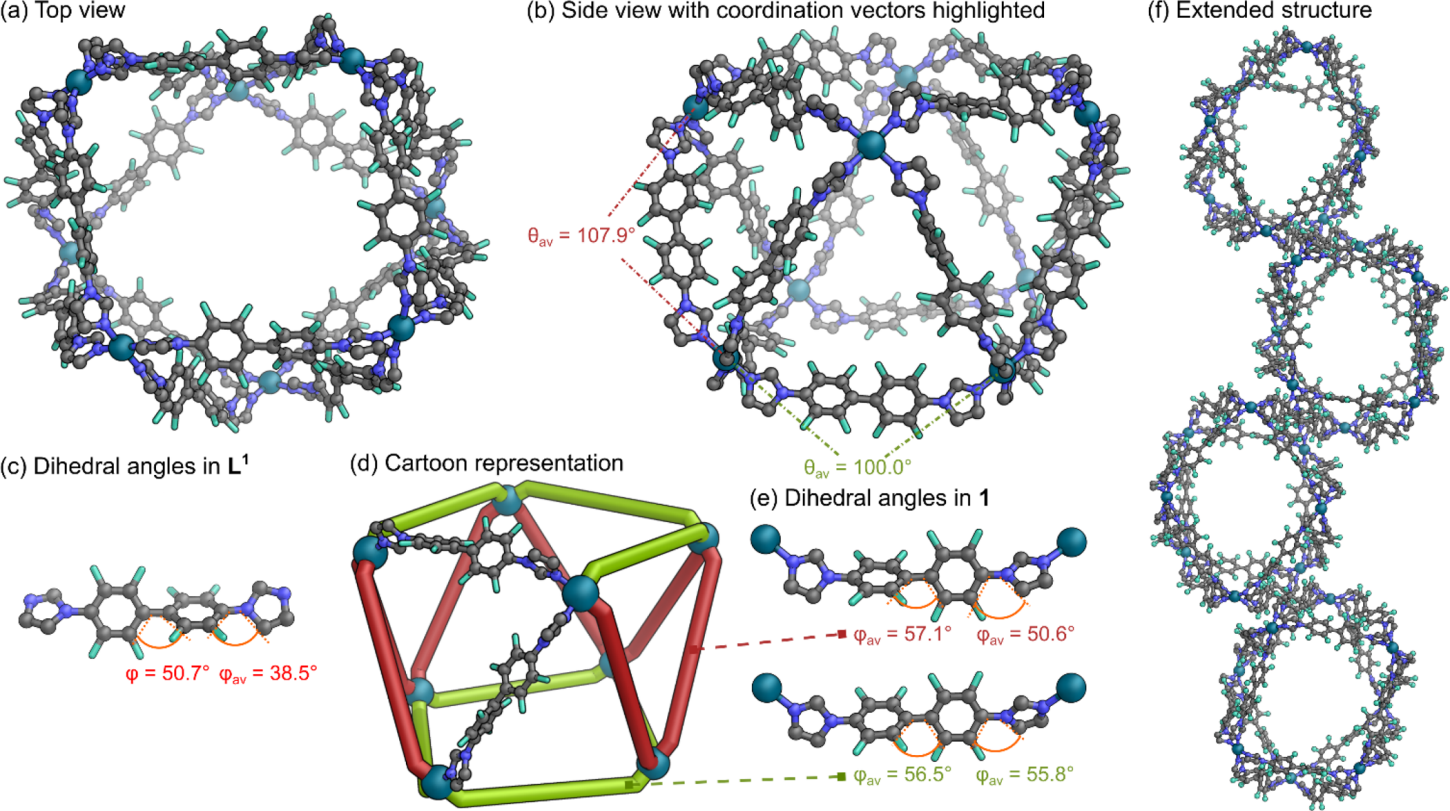
(a) Top-down view of SCXRD structure of cage **1**. (b) Side view of SCXRD structure of cage **1**, with average metal–ligand coordination vector angle, θ_av_, indicated for both ligand environments. (c) SCXRD structure of ligand **L** ^**1**^, with dihedral twist angles, φ, indicated. (d) Cartoon representation of cage **1**, highlighting the two different ligand environments: triangle/triangle edge = red; square/triangle edge = green. (e) Fragments of SCXRD structure of cage **1**, illustrating the average dihedral twist angles, φ, in both triangle/triangle edges and square/triangle edges. (f) Extended solid-state structure of cage **1**. Hydrogen atoms, disorder, solvents, and counteranions have been omitted for clarity. Color: C = gray, *N* = blue, *F* = aquamarine, Pd = turquoise.

With complex **1** being thermodynamically stable, various crystallization conditions were attempted. Slow vapor diffusion of EtOAc into a DMSO solution of **1** resulted in block-shaped crystals after 10 days at 22 °C. SCXRD analysis was performed using a synchrotron radiation source at 100 K. High quality data up to 1.3 Å resolution was obtained (this is considerably better than the typical resolution obtained in crystal data for large Pd_*n*_L_2*n*_ structures). The solid-state structure of cage **1** was unambiguously determined to be a Pd_8_L_16_ distorted square antiprism (Figure 3a,b,f and Figure S53), was refined in space group *P*2_1_/*n*, and contains a complete cage molecule in the asymmetric unit. Using the CageCavityCalc (*C*3) program,^29^ we determined that the void space within **1** was 5420 Å^3^ (Figure S52). The void diameter was ∼ 26.8 Å. The calculated electrostatic surface potential of cage **1** also differs markedly from the other cages in this work due to the inverted quadrupole moment of the perfluorobenzene rings. However, fullerenes were not observed to bind, likely due to the large cavity volume. Interestingly, in the extended solid state structure (Figure 3f) the cages were observed to pack in such a way that channels exist throughout the material (Figure S54) and we intend to investigate applications of these in future work.

In the idealized *D*_4d_ point group structure of a square antiprism, the two square faces (consisting of eight ligands total) are rotated 45° with respect to each other and are connected by eight additional ligands bridging pairs of eight triangular faces (Figure 3d). This cage present in the crystal structure is distorted from the ideal square antiprismatic geometry with distortion of the square faces toward more rhomboidal shape. The lengths of the edges of the square antiprism, as defined by the adjacent Pd to Pd distances encompass the range 16.7–17.6 Å. Analysis of the metal–ligand coordination vectors, determined a value of θ_av_ = 104.0° ± 7.5°, close to the ideal angle of 105.1° in a square antiprism, with a slightly larger angle of θ_av_ = 107.9° ± 7.2° for ligands on triangle/triangle edges than for ligands on square/triangle edges, where θ_av_ = 100.0° ± 5.5° (Figure 3b and Tables S1–S2). The overall dihedral twist angle was φ_av_ = 56.8° ± 4.2° between the perfluorophenyl rings, with similar twists along triangle/triangle and square/triangle edges (Figure 3e and Tables S3–S4). Between the imidazole and perfluorophenyl rings there was an overall twist of φ_av_ = 53.2° ± 11.7°, with a slightly larger twist of φ_av_ = 55.8° ± 14.4° along square/triangle edges than along triangle/triangle edges, φ_av_ = 50.6° ± 7.3° (Tables S5–S6). The underlying conformational preferences of the free ligand (Figure 3c) are reflected in the conformational preferences of the coordinated ligand, justifying our choice of perfluorobenzene linking groups in targeting formation of the Pd_8_ **L** ^**1**^_16_ square antiprism. Importantly, the perfluorophenyl rings induced twists along all three biaryl bonds, a factor we believe to be crucial in driving formation of the square antiprism.

While the introduction of fluorines had biased the inherent conformational preference of the ligand away from planarity, we were curious as to whether they were a prerequisite for square antiprism formation. While the analogous nonfluorous diimidazole ligand **L** ^**2**^ lacks this inherent preference for the twisted conformation,^30^ there is a low barrier to rotation around its biaryl bonds, and the twisted conformations are energetically accessible.

Whereas cages from fluorous ligand **L** ^**1**^ are reported in this work for the first time, nonfluorous ligand **L** ^**2**^ has been previously reported to assemble into a Pd_6_ **L** ^**2**^_12_ octahedron by Mukherjee and co-workers, with a SCXRD structure reported with a PF_6_^–^ counteranion.^19^ In all self-assemblies, there is an entropic driving force to form the smallest geometrically feasible architecture. However, many factors including solvent and counteranion can direct assembly to particular structures.^31^ In order to have a direct comparison with our fluorous assembly **1**, and to exclude any anion effects, we investigated assembly of **L** ^**2**^ ourselves with Pd­(NO_3_)_2_. After 3 h at 100 °C in DMSO-*d*_6_, we too observed the formation of Pd_6_ **L** ^**2**^_12_ octahedron **2** with the ^1^H NMR spectrum containing a single major set of ligand signals, as expected for a symmetric octahedral assembly (Figures S23–S26).

We grew block-shaped single crystals of Pd_6_ **L** ^**2**^_12_ octahedron **2** with NO_3_^–^ as the counteranion, through slow vapor diffusion over 1 week of EtOAc into a DMSO solution of **2** at 22 °C. The SCRXD structure revealed the expected Pd_6_ **L** ^**2**^_12_ octahedron, with idealized *O*_h_ point group symmetry (Figure 4a and Figure S55). Analysis of the average metal–ligand coordination vectors revealed θ_av_ = 93.3° ± 3.4°, close to the ideal angle of 90° for an octahedron (Table S7). The overall dihedral twist angle within cage **2** was determined as φ_av_ = 31.5° ± 3.7° between the benzene rings and φ_av_ = 30.7° ± 9.8° between the benzene and imidazole rings (Figure 4b and Tables S8–S9), both twist angles considerably less than in cage **1**. Block-shaped single crystals of ligand **L** ^**2**^ were also grown through vapor diffusion of hexane into chloroform over 1 week. SCXRD analysis revealed, as expected, that free ligand **L** ^**2**^ exhibited less preference for twisting, with a twist of φ = 20.7° between the benzene rings and φ = 8.7° and 16.2° between the benzene and imidazole rings (Figure 4c and Table S14). As with biphenyl itself, the energetic gain from stronger intermolecular interactions present in a more efficiently packed crystal structure with smaller dihedral twists likely outweighs any favorable energetic contribution from greater twisting.

**Figure 4:**
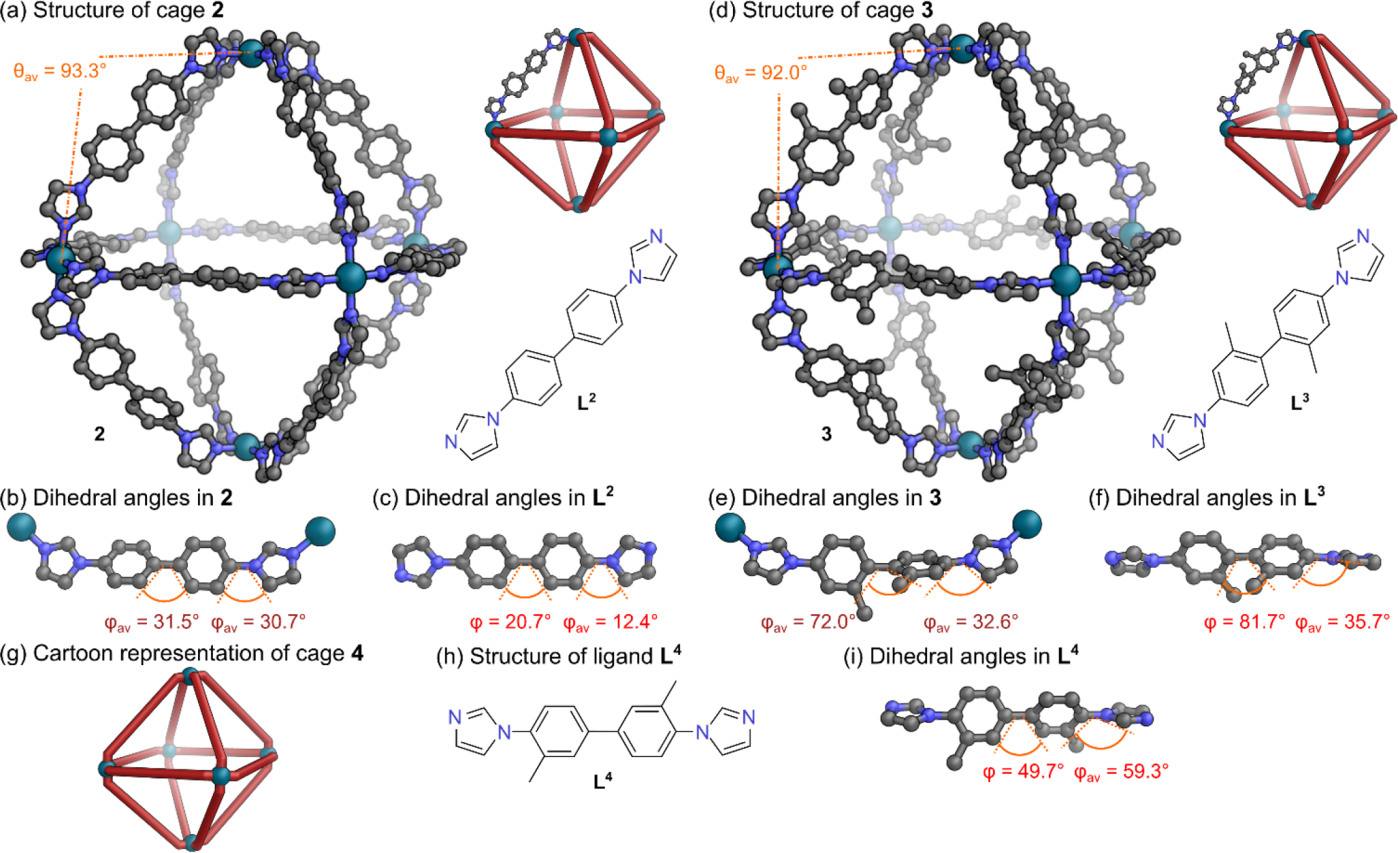
(a) SCXRD structure and cartoon representation of octahedral cage **2**, with average metal–ligand coordination vector angle, θ_av_, indicated, and structure of ligand **L** ^**2**^. (b) Fragment of SCXRD structure of cage **2**, with dihedral twist angles, φ, indicated. (c) SCXRD structure of ligand **L** ^**2**^, with dihedral twist angles, φ, indicated. (d) SCXRD structure and cartoon representation of octahedral cage **3**, with average metal–ligand coordination vector angle, θ_av_, indicated, and structure of ligand **L** ^**3**^. (e) Fragment of SCXRD structure of cage **3**, with dihedral twist angles, φ, indicated. (f) SCXRD structure of ligand **L** ^**3**^, with dihedral twist angles, φ, indicated. (g) Cartoon representation of octahedral cage **4**. (h) Chemical structure of ligand **L** ^**4**^. (i) SCXRD structure of ligand **L** ^**4**^, with dihedral twist angles, φ, indicated. Hydrogen atoms, disorder, solvents, and counteranions have been omitted for clarity. Color: C = gray, *N* = blue, Pd = turquoise.

The original paper on the Pd_6_ **L** ^**2**^_12_ octahedron reported that the ^1^H NMR spectra became considerably more complex during variable temperature experiments at low temperature, something that was attributed to rotamers within the biphenyl ligands. When we reran the ^1^H NMR spectrum of our sample after leaving it for 1 week at 25 °C (Figure S45), we observed the presence of numerous additional signals, which we can now conclusively confirm are due to the additional presence of a second structure, Pd_8_ **L** ^**2**^_16_ square antiprism **2’**. This second structure possesses two sets of signals per ligand environment as expected. DOSY NMR analysis after 1 week at 25 °C confirmed the presence of two different sized assemblies, with Pd_6_ **L** ^**2**^_12_ octahedron **2** having a log *D* = – 9.98 and Pd_8_ **L** ^**2**^_16_ square antiprism **2’** having a log *D* = – 10.05 (Figure S45). ESI-MS analysis also confirmed the presence of Pd_8_ **L** ^**2**^_16_ square antiprism assembly **2’** (Figures S47–S48). There was a small further increase in the amount of square antiprism **2’** after 3 weeks at 25 °C (with approximately equal amounts of **2** and **2’** present at this point), and there were no further changes after leaving the solution for considerably longer, suggesting equilibrium had been reached. Variable temperature ^1^H NMR analysis of this equilibrated sample (Figure S46) showed that square antiprism **2’** converted back into octahedron **2** relatively quickly at higher temperatures. We were unable to crystallize Pd_8_ **L** ^**2**^_16_ square antiprism assembly **2’** from this mixture.

The fact that initial NMR analysis performed immediately after synthesis revealed just the octahedron can be attributed to the entropic preference for smaller assemblies at higher temperatures. While the square antiprism is energetically accessible for nonfluorous ligand **L** ^**2**^, the flexibility in **L** ^**2**^ and the ability to adopt nearly planar conformations with limited dihedral twisting means the octahedron is formed preferentially.

The nonplanar conformations enforced by perfluorination of the central rings appeared crucial to drive exclusive square antiprism formation, as they drive twists between the benzene rings, and between the imidazole and the benzene rings. However, as previously discussed, there are several reasons why the fluorine atoms impart this effect (their slightly larger size compared to hydrogen, their high electronegativity, and their effect on the orbital energies reducing effective conjugation between rings). To further examine the interplay of these factors, we designed another similar ligand **L** ^**3**^, containing methyl groups in the 2 and 2’-position of the biphenyl moiety. These methyl groups have a significantly greater steric demand than a fluorine atom, however they lack the same electronic influence. While they enforce a larger twist around the biphenyl bond, they have less influence on the twist between the benzene and imidazole rings.

Ligand **L** ^**3**^ was synthesized with an Ullmann coupling (Section S4.2 in the Supporting Information). Block-shaped single crystals of **L** ^**3**^ were grown through vapor diffusion of hexane into chloroform over 1 week. SCXRD analysis revealed that free ligand **L** ^**3**^ exhibited a substantial dihedral twist angle φ = 81.7° between the benzene rings and smaller twists of φ = 34.5° and 36.8° between the benzene and imidazole rings (Figure 4f and Table S15). Self-assembly of **L** ^**3**^ with Pd­(NO_3_)_2_ (3 h at 100 °C in DMSO-*d*_6_), gave a structure with a single set of signals in the ^1^H NMR spectrum, suggesting that the symmetric Pd_6_ **L** ^**3**^_12_ octahedral assembly **3** had formed (Figures S30–S32). This octahedral assembly was further confirmed by ESI-MS analysis (Figures S35–S36) and DOSY NMR spectroscopy with log *D* = – 9.98 (Figure S33).

Block-shaped single crystals suitable for SCXRD analysis were grown by slow vapor diffusion of EtOAc into a DMSO solution of **3** over 10 days, with the solid-state structure unequivocally confirming the symmetric Pd_6_ **L** ^**3**^_12_ structure of **3**, with idealized *O*_h_ point group symmetry (Figure 4d and Figure S56). Analysis of the average metal–ligand coordination vectors revealed θ_av_ = 92.0° ± 5.5°, close to the ideal angle of 90° for an octahedron (Table S10). The overall dihedral twist angle within cage **3** was determined as φ_av_ = 72.0° ± 5.3° between the benzene rings and φ_av_ = 32.6° ± 4.1° between the benzene rings and imidazole rings (Figure 4e and Tables S11–S12). There was no change in the ^1^H NMR spectrum both after 1 week and 3 weeks at 25 °C indicating thermodynamic stability (Figure S49).

We also investigated the effect of instead placing the methyl groups on the benzene ring *ortho* to the imidazole. To test this, ligand **L** ^**4**^ was synthesized with an Ullmann coupling (Section S4.3 in the Supporting Information). Block-shaped single crystals of **L** ^**4**^ were grown through slow evaporation of a dichloromethane solution over 2 days. SCXRD analysis revealed that free ligand **L** ^**4**^ exhibited a dihedral twist angle φ = 49.7° between the benzene rings and an average twist of φ_av_ = 59.3° between the benzene and imidazole rings (Figure 4i and Table S16). Self-assembly of **L** ^**4**^ with Pd­(NO_3_)_2_ (3 h at 100 °C in DMSO-*d*_6_), gave a structure with a single set of signals in the ^1^H NMR spectrum, suggesting that the symmetric Pd_6_ **L** ^**4**^_12_ octahedral assembly **4** had formed (Figures S37–S39). This octahedral assembly was further confirmed by ESI-MS analysis (Figures S42–S43) and DOSY NMR spectroscopy with log *D* = – 10.2 (Figure S40).

Overall, in both methyl-substituted examples (SCXRD structures of ligands **L** ^**3**^ and **L** ^**4**^ and cage **3**), the methyl group always induces a substantial twist in whatever biaryl bond is *ortho* to the methyl group. However, the other biaryl bond remains unaffected. While this can sometimes also be twisted (driven by crystal packing effects particularly in the free ligand), crucially it does not have to be twisted, and conformations with less twisting remain energetically accessible. Hence, neither ligand **L** ^**3**^ nor **L** ^**4**^ give square antiprism. This highlights that significant dihedral twisting is required in all three biaryl connections in these systems to diverge the coordination vectors enough to drive square antiprism formation. Perfluorination of the ligand is an easy way to achieve this twist through altering the electronics of the ligand. Conversely, accessing twisted ligands through steric effects from multiple substituents is synthetically much more laborious.

## Conclusions

In summary, we report square antiprismatic metal–organic cage **1**, as the first targeted Pd_8_L_16_ assembly in the Pd_*n*_L_2*n*_ family. This previously elusive and metastable structure has been stabilized both in solution and in the solid state through fine-tuning of the ligand conformational preferences through perfluorination of the biphenyl ligand backbone. The presence of fluorine induces dihedral twists along all three biaryl bonds of the ligand (perfluorobenzene-perfluorobenzene and perfluorobenzene-imidazole). This twisting diverges the imidazole coordination motifs considerably beyond the 90° required for an octahedron and close to the ideal angle of 105.1° for a square antiprism. Perfluorination is crucial to drive this change in structure, with methyl-substituted ligands **L** ^**3**^ and **L** ^**4**^, which have a significantly larger dihedral twist along one type of biaryl bond but not the other, still only producing the simpler octahedron. While Pd_*n*_L_2*n*_ cages have largely been constructed through structural planning in two dimensions with planar ligand moieties, this work demonstrates how well-engineered dihedral twisting^32^ allows access into three-dimensional space. Within the Pd_*n*_L_2*n*_ family, all previously reported true polyhedral structures–those lacking double-walled edges–belong to high-symmetry point groups (e.g., *O*_h_ and *I*_h_), characterized by multiple highest-order rotational axes. In contrast, the *D*_4d_ square antiprism presented here represents the first thermodynamically stable member to be characterized from a lower-symmetry group, possessing only a single principal rotational axis. We believe this strategy of exploiting dihedral twists may have wide scope for accessing new types of previously inaccessible supramolecular constructs, both within the metal–organic cage field, and in other classes of self-assembled supramolecular materials, and work to examine this further is currently ongoing in our laboratory.

## Supplementary Material



## Notes

Experimental procedures, NMR spectra (^1^H, COSY, DOSY, ^13^C, ^19^F), high-resolution ESI-MS, and analysis of the SCXRD data (PDF)
