# Supplementary material for: Fluorinated Twists: A Pathway to a Stable Pd8L16 Square Antiprism
Source: J Am Chem Soc. 2025 Aug 8;147(33):30296–303. doi: 10.1021/jacs.5c09573 (PMC12371867; doi:10.1021/jacs.5c09573)
Supplement: Supplementary file 1 [file ja5c09573_si_001.pdf]

Supporting Information for:

Fluorinated twists: A pathway to a stable Pd_8L_{16} square antiprism

Soumalya Bhattacharyya,[a] Stephen P. Argent,[a] and Ben S. Pilgrim[a*]

[a]*School of Chemistry, University of Nottingham, University Park, Nottingham, NG7 2RD, UK*

*E-mail: ben.pilgrim@nottingham.ac.uk

Contents

S1 Reagents and general materials ... **4**

S2 Characterisation and analysis methods ... **5**

S2.1 NMR spectroscopy ... 5

S2.2 Mass spectrometry ... 5

S2.3 Access to raw data ... 5

S3 Ligand reference chart ... **6**

S3.1 Ligand reference chart ... 6

S4 Ligand synthesis and characterisation ... **7**

S4.1 Synthesis of 1,1'-(perfluoro-[1,1'-biphenyl]-4,4'-diyl)bis(1H-imidazole) L^1 ... 7

S4.2 Synthesis of 1,1'-(2,2'-dimethyl-[1,1'-biphenyl]-4,4'-diyl)bis(1H-imidazole) L^3 ... 11

S4.3 Synthesis of 1,1'-(3,3'-dimethyl-[1,1'-biphenyl]-4,4'-diyl)bis(1H-imidazole) L^4 ... 14

S5 Synthesis and characterisation of metal-organic cages ... **17**

S5.1 Self-assembly of $Pd_8L^1{}_{16}$ square antiprism 1 ... 17

S5.2 Self-assembly of $Pd_6L^2{}_{12}$ octahedron 2 ... 23

S5.3 Self-assembly of $Pd_6L^3{}_{12}$ octahedron 3 ... 28

S5.4 Self-assembly of $Pd_6L^4{}_{12}$ octahedron 4 ... 33

S6 Stability studies of cage systems ... **38**

S6.1 Cage 1 ... 38

S6.2 Cage 2 ... 38

S6.2.1 Variable temperature NMR of cage 2 ... 39

S6.3 Cage 3 ... 40

S7 Determination of ligand vector angles and dihedral angles ... **41**

S7.1 Coordination vectors in $Pd_8L^1{}_{16}$ square antiprism 1 ... 43

S7.2 Dihedral twist angles in $Pd_8L^1{}_{16}$ square antiprism 1 ... 45

S7.3 Coordination vectors in $Pd_6L^2{}_{12}$ octahedron 2 ... 47

S7.4 Dihedral twist angles in $Pd_6L^2{}_{12}$ octahedron 2 ... 48

S7.5 Coordination vectors in $Pd_6L^3{}_{12}$ octahedron 3 ... 49

S7.6 Dihedral twist angles in $Pd_6L^3{}_{12}$ octahedron 3 ... 50

S7.7 Dihedral twist angles in ligand L^1 ... 51

S7.8 Dihedral twist angles in ligand L^2 ... 51

S7.9 Dihedral twist angles in ligand L^3 ... 51

S7.10 Dihedral twist angles in ligand L^4 52

S8 Cavity modelling 53

S9 X-ray crystallography 54

S9.1 Single crystal structure of $Pd_8L^1_{16}$ square antiprism 1 56

S9.1.1 Specific crystal structure and refinement details for 1 56

S9.2 Single crystal structure of cage 2 58

S9.2.1 Specific crystal structure and refinement details for 2 58

S9.3 Single crystal structure of cage 3 60

S9.3.1 Specific crystal structure and refinement details for 3 60

S9.4 Single crystal structure of ligand L^1 62

S9.4.1 Specific crystal structure and refinement details for L^1 62

S9.5 Single crystal structure of ligand L^2 63

S9.5.1 Specific crystal structure and refinement details for L^2 63

S9.6 Single crystal structure of ligand L^3 64

S9.7 Single crystal structure of ligand L^4 65

S10 References 66

S1 Reagents and general materials

All chemicals and dry solvents were purchased from Sigma-Aldrich or Fluorochem. Commercial solvents and reagents were used without further purification unless specified. Bottles of $Pd(NO_3)_2•2H_2O$ were observed to degrade slightly over time, even when stored in a desiccator, presumably due to excess water content; best results were obtained with fresh bottles of $Pd(NO_3)_2•2H_2O$. Flash column chromatography was performed using silica gel high purity grade (pore size 60 Å, 230–400 mesh particle size, Sigma-Aldrich). Automated flash column chromatography was performed using a CombiFlash NextGen 300 Automated Flash Chromatography System, with a UV-Vis detector 200-800 nm with PeakTrak software control via a 12" capacitive touchscreen, with a flow rate range of 1-300 mL min^{-1} and a maximum pressure limit of 160 psi (11 bar). RediSep silver disposable flash silica columns were used to purify compounds. TLC analyses were performed on Merck TLC silica gel 60 F_{254} plates. Product spots were visualised under UV light (λ_{max} = 254 nm) and/or by staining with potassium permanganate or vanillin solutions. Centrifugation was carried out on a Grant-bio LMC-3000 or a Corning Mini Microcentrifuge. All reactions were stirred with magnetic followers. Room temperature is taken as 293K.

S2 Characterisation and analysis methods

S2.1 NMR spectroscopy

NMR spectra were recorded at 298 K using Bruker Avance(III) 500 or 400 spectrometers. (^{1}H, 500 / 400 MHz; $^{13}C[^{1}H]$ and $^{13}C[^{19}F]$, 125 / 101 MHz; ^{19}F, 376 MHz). Spectrometers were automatically tuned and matched to the correct operating frequencies. Routine ^{1}H NMR characterisation was carried out using a zg30 pulse program (30° pulse). ^{1}H and ^{13}C NMR spectra are referenced to the residual solvent peak for DMSO (^{1}H: 2.50 ppm, ^{13}C: 39.53 ppm) or an internal standard of hexafluorobenzene (^{19}F: −164.90 ppm). Deuterated dimethylsulfoxide (DMSO) was obtained from Sigma Aldrich and was used without any further purification. NMR signals are reported in terms of chemical shift (δ) in parts-per-million (ppm), multiplicity, coupling constants (in Hz), and relative integral in that order. The following abbreviations for multiplicity are used: s, singlet; d, doublet; t, triplet; qu, quartet; qn, quintet; m, multiplet; br, broad. Where spectra have been assigned this has been done on accompanying figures. Spectra were digitally processed (phase and baseline corrections, integration, peak analysis) using Mestrenova 14.0.0-23239. DOSY NMR experiments were performed on 5 mm BBO probe and Standard QUAD Probe on a Bruker Avance(III) 500 MHz, NMR spectrometer. Gradient strength was between 1.73 and 17.5 G/cm. DOSY measurements were performed using the standard pulse program, dstebpgp3s, employing a stimulated echo and longitudinal eddy-current delay (LED) using bipolar gradient pulses for diffusion using two spoil gradients. SinE.100 gradients were used. Diffusion times Δ = 200 ms and δ = 2000 μs were used for the experiments. The size of fid = 32. Individual rows of the S4 psuedo-2D diffusion databases were phased and baseline corrected. Raw DOSY data were processed using the Peak fit DOSY transform programme in Mestrenova 14.0.0-23239.

S2.2 Mass spectrometry

High resolution electrospray ionisation (ESI) mass spectra were obtained on a Bruker ESI-TOF MicroTOF II spectrometer or a Bruker Impact II. The exported raw data were processed on Data Analysis software to access them in a .csv format, which then was used to plot in OriginPro. For the cage samples in DMSO, dilution was performed with acetonitrile (1:1, DMSO-acetonitrile, v/v) before subjecting them to ESI.

S2.3 Access to raw data

Raw data are available upon reasonable request to the corresponding author Dr Ben Pilgrim ben.pilgrim@nottingham.ac.uk.

S3 Ligand reference chart

S3.1 Ligand reference chart

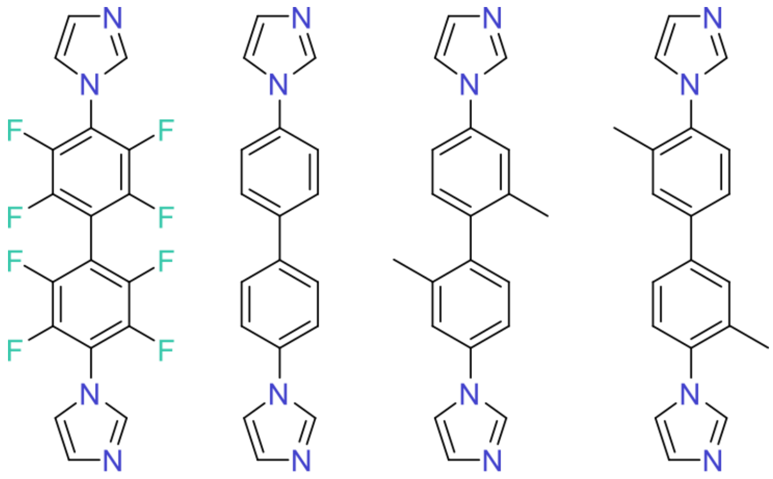

L^1 L^2 L^3 L^4

Figure S1: Ligand reference table

S4 Ligand synthesis and characterisation

Ligand **L²** was synthesised according to a previous literature procedure.[1]

S4.1 Synthesis of 1,1'-(perfluoro-[1,1'-biphenyl]-4,4'-diyl)bis(1H-imidazole) L¹

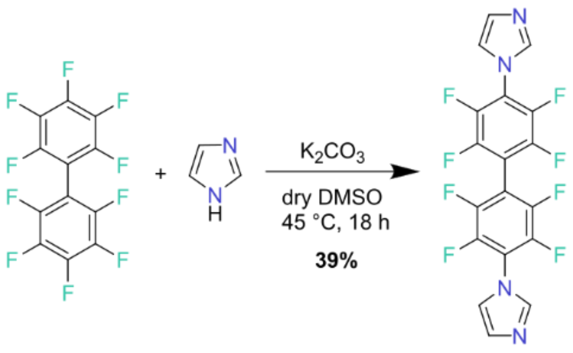

L¹ was synthesised using a modification of a previous literature procedure.[2] A flame-dried 100 mL double-necked flask was charged with decafluorobiphenyl (668 mg, 2.00 mmol), imidazole (544 mg, 8.00 mmol), and potassium carbonate (1104 mg, 8.00 mmol) and degassed. Freshly dried and degassed DMSO (20 mL) was transferred to the flask which was then heated at 45 °C for 18 h. The resultant pale-yellow reaction mixture was added to ice-cold water (80 mL) and extracted with CH_2Cl_2 (40 mL × 3). The organic layer was again washed with brine and collected over Na_2SO_4, filtered, and the solvent removed *in vacuo*. The crude mixture was purified using silica gel column chromatography with THF:CH_2Cl_2 (1:9) as the eluent to afford ligand **L¹** as a white crystalline solid (336 mg, 0.78 mmol, 39%).

^{1}H NMR (500 MHz, DMSO-d_6) δ_H: 8.15 (s, 2H), 7.67 (s, 2H), 7.15 (s, 2H).

^{19}F NMR (376 MHz, DMSO-d_6) δ_F: −140.77 (m, 4F), −149.42 (q, 4F).

^{13}C NMR [^{1}H] (125 MHz, DMSO-d_6) δ_C: 144.2 (dd, *J* = 251.6, 14.1 Hz), 142.2 (dd, *J* = 251.8, 13.6 Hz), 138.8, 130.0, 121.4, 120.0 (t, *J* = 13.7 Hz), 105.2 (t, *J* = 15.4 Hz).

^{13}C NMR [^{19}F] (101 MHz, DMSO-d_6) δ_C: 143.8, 141.8, 138.3 (ddd, *J* = 216.6, 10.5, 6.3 Hz), 129.6 (dt, *J* = 191.6, 10.6 Hz), 121.0 (ddd, *J* = 196.2, 17.0, 3.2 Hz), 119.5, 104.7.

ESI-MS (ESI, MeCN), *m/z*: calculated for $[M+H]^+$, $[C_{18}H_7F_8N_4]^+$, 431.0537, found 431.0557.

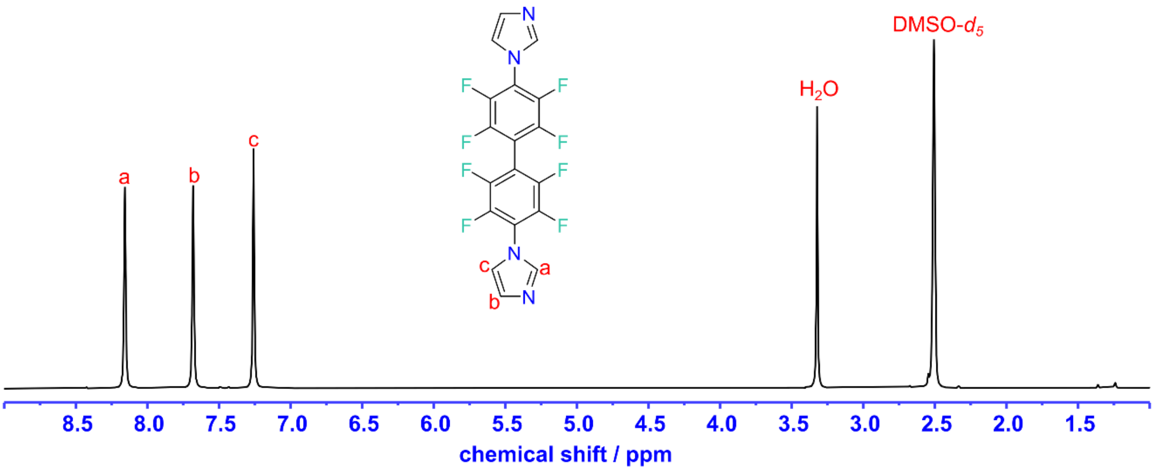

Figure S2: ^{1}H NMR spectrum of **L^1**
(500 MHz, 298 K, DMSO-d_6)

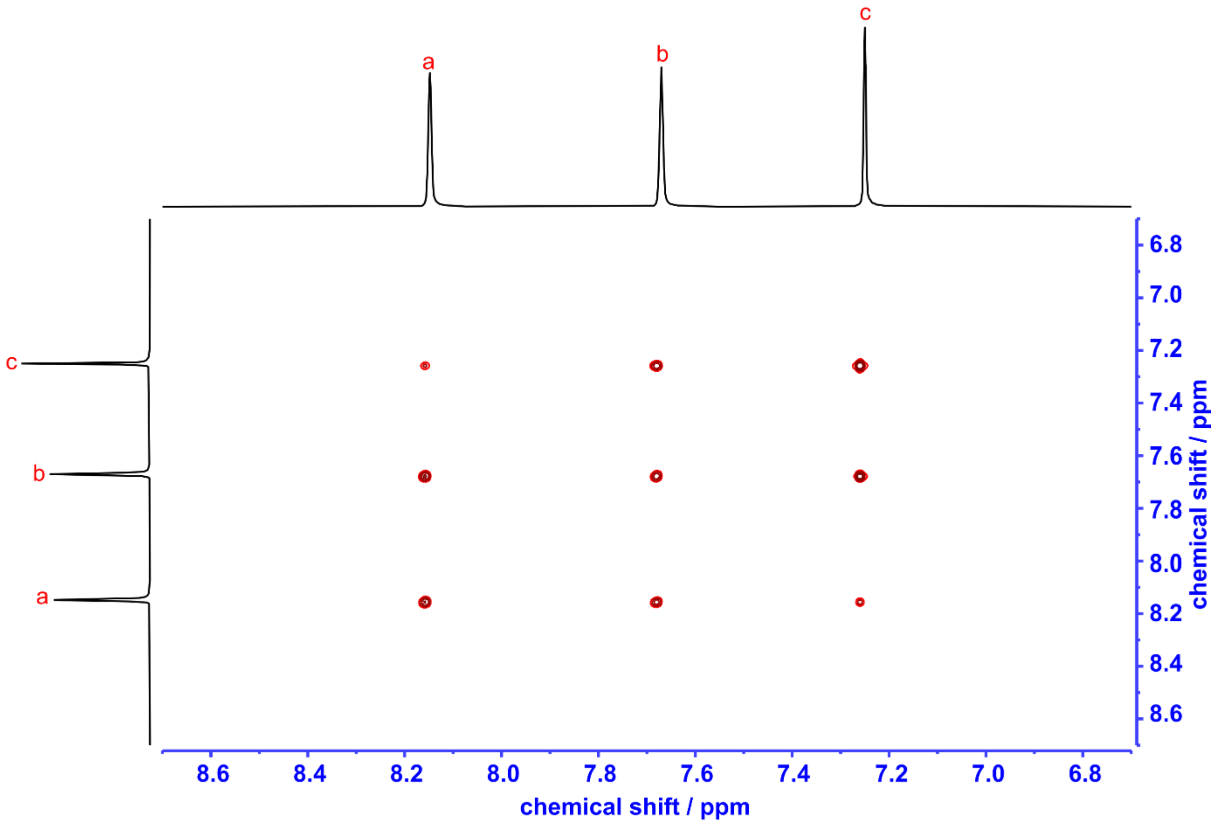

Figure S3: ^{1}H-^{1}H COSY spectrum of **L^1**
(500 MHz, 298 K, DMSO-d_6)

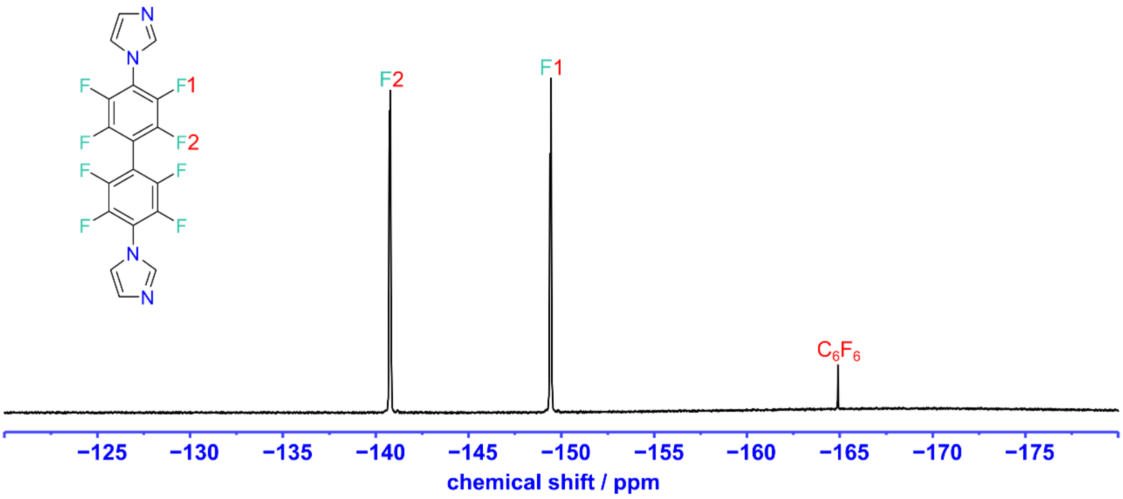

Figure S4: ^{19}F NMR spectrum of **L^1**
(376 MHz, 298 K, DMSO-*d*6)

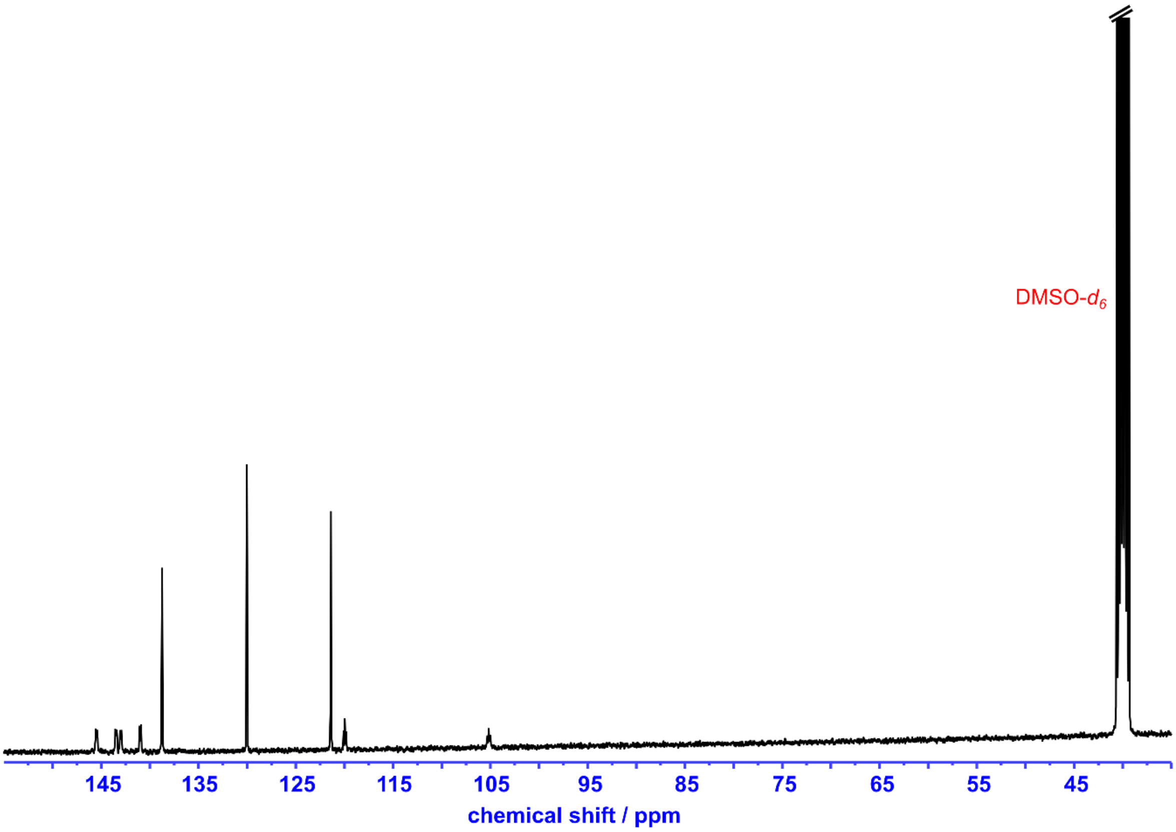

Figure S5: ^{13}C[^{1}H] NMR spectrum of **L^1**
(125 MHz, 298 K, DMSO-*d*6)

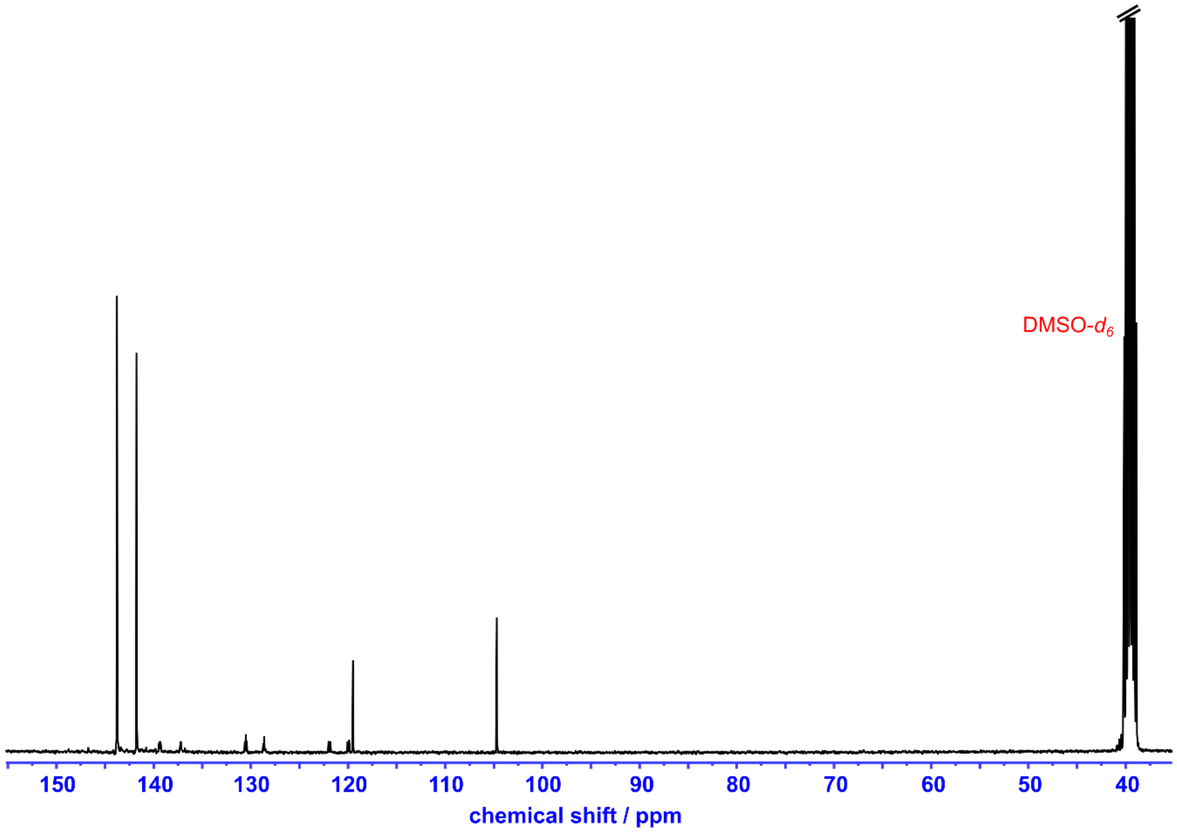

Figure S6: $^{13}C[^{19}F]$ NMR spectrum of $\mathbf{L^1}$
(101 MHz, 298 K, DMSO-d_6)

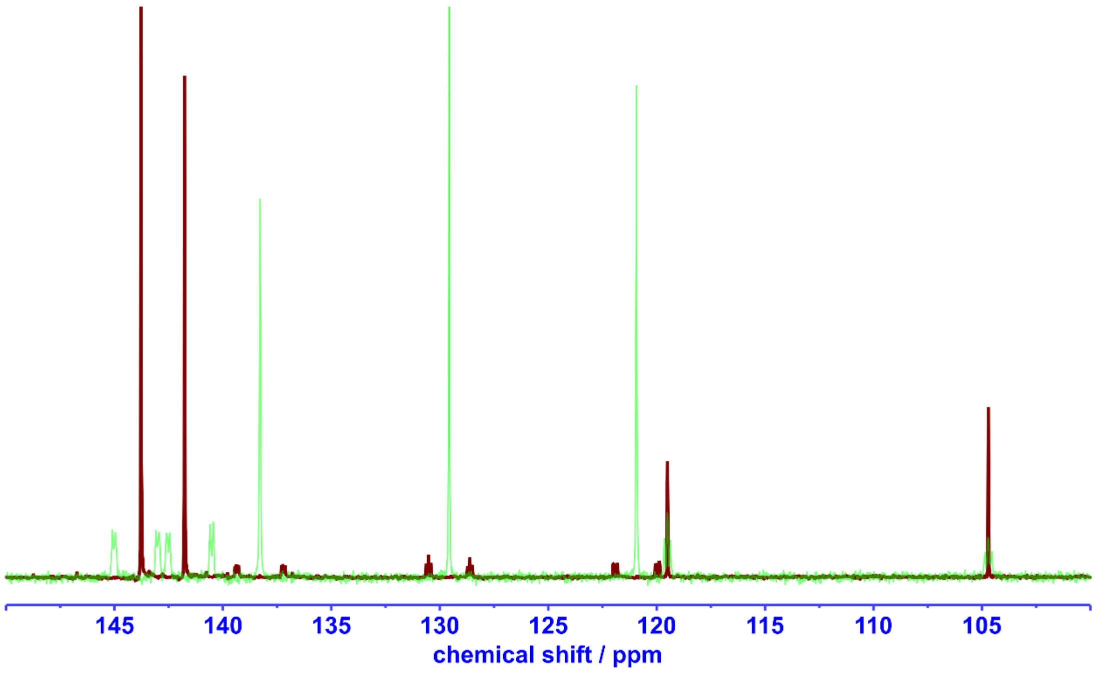

Figure S7: Stack plot of $^{13}C[^{19}F]$ NMR spectrum (maroon) (101 MHz, 298 K, DMSO-d_6) and $^{13}C[^{1}H]$ NMR spectrum (lime) (125 MHz, 298 K, DMSO-d_6) of $\mathbf{L^1}$

S4.2 Synthesis of 1,1'-(2,2'-dimethyl-[1,1'-biphenyl]-4,4'-diyl)bis(1H-imidazole) L³

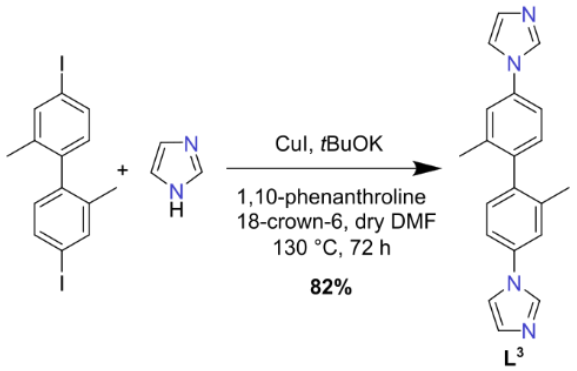

A flame-dried 100 mL double-necked flask was charged with CuI (38 mg, 0.20 mmol), 1,10-phenanthroline (72 mg, 0.40 mmol) and dry DMF (10 mL). After degassing the solution was heated at 120 °C for 5 min. To this mixture 4,4'-diiodo-2,2'-dimethyl-1,1'-biphenyl (868 mg, 2.00 mmol), imidazole (1090 mg, 16.0 mmol), potassium *tert*-butoxide (2240 mg, 20.0 mmol), and two crystals of 18-crown-6 were added and the mixture heated at 130 °C for 72 h under N_2 flow. The resultant thick brown slurry was added to ice-cold water (50 mL) and extracted with CH_2Cl_2 (50 mL × 2). The organics were collected over Na_2SO_4, filtered, and the solvent removed *in vacuo*. The crude mixture was purified using silica gel column chromatography with THF:DCM (1:5) as the eluent. Finally, the solid was washed Et_2O (3 × 15 mL) to remove of excess 18-crown-6 to afford ligand **L³** as a white crystalline solid (515 mg, 1.64 mmol, 82%).

^{1}H NMR (500 MHz, DMSO-d_6) δ_H: 8.30 (s, 2H), 7.79 (s, 2H), 7.66 (d, J = 2.4 Hz, 2H), 7.54 (dd, J = 8.2 Hz, 2.4 Hz, 2H), 7.24 (d, J = 8.2 Hz, 2H), 7.13 (s, 2H), 2.10 (s, 6H).

^{13}C NMR (125 MHz, DMSO-d_6) δ_C: 138.6, 137.4, 136.1, 135.5, 130.5, 129.9, 121.7, 118.0, 117.7, 19.6.

ESI-MS (ESI, MeCN), *m/z*: calculated for $[M+H]^+$, $[C_{20}H_{19}N_4]^+$, 315.1604, found 315.1611.

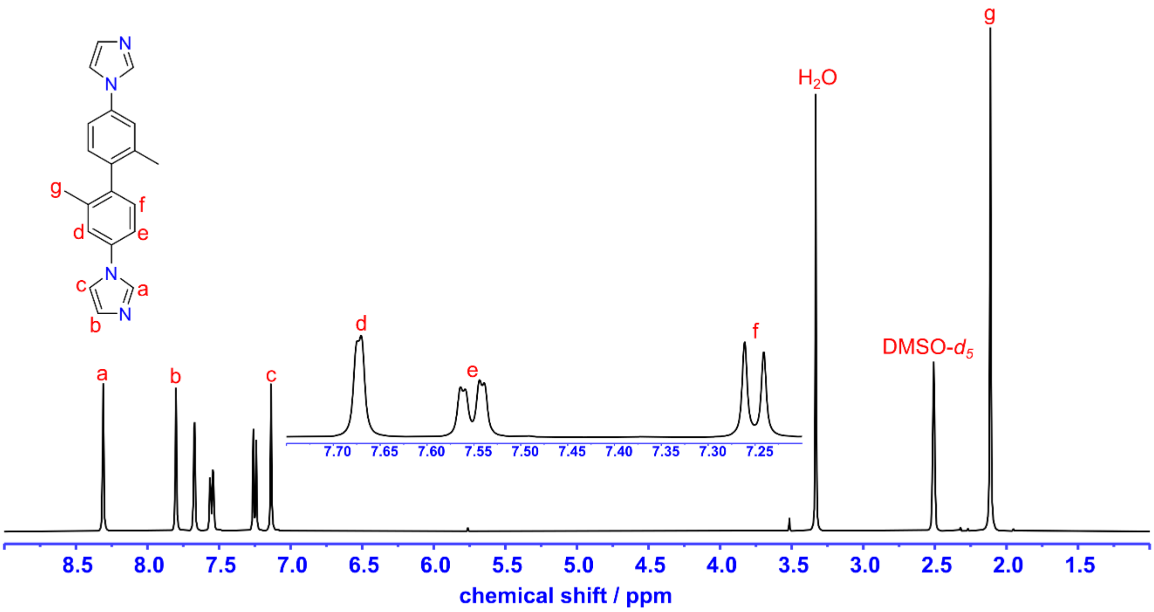

Figure S8: ^{1}H NMR spectrum of **L^3**
(500 MHz, 298 K, DMSO-d_6)

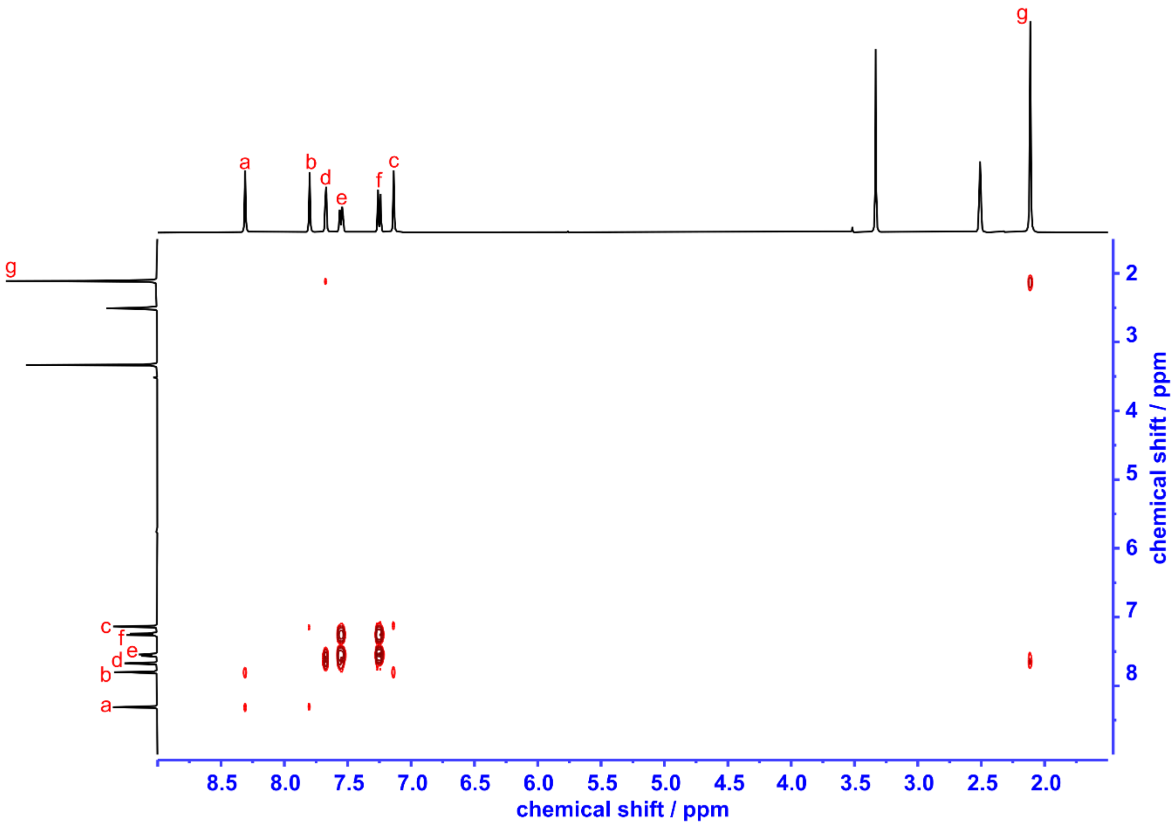

Figure S9: ^{1}H-^{1}H COSY spectrum of **L^3**
(500 MHz, 298 K, DMSO-d_6)

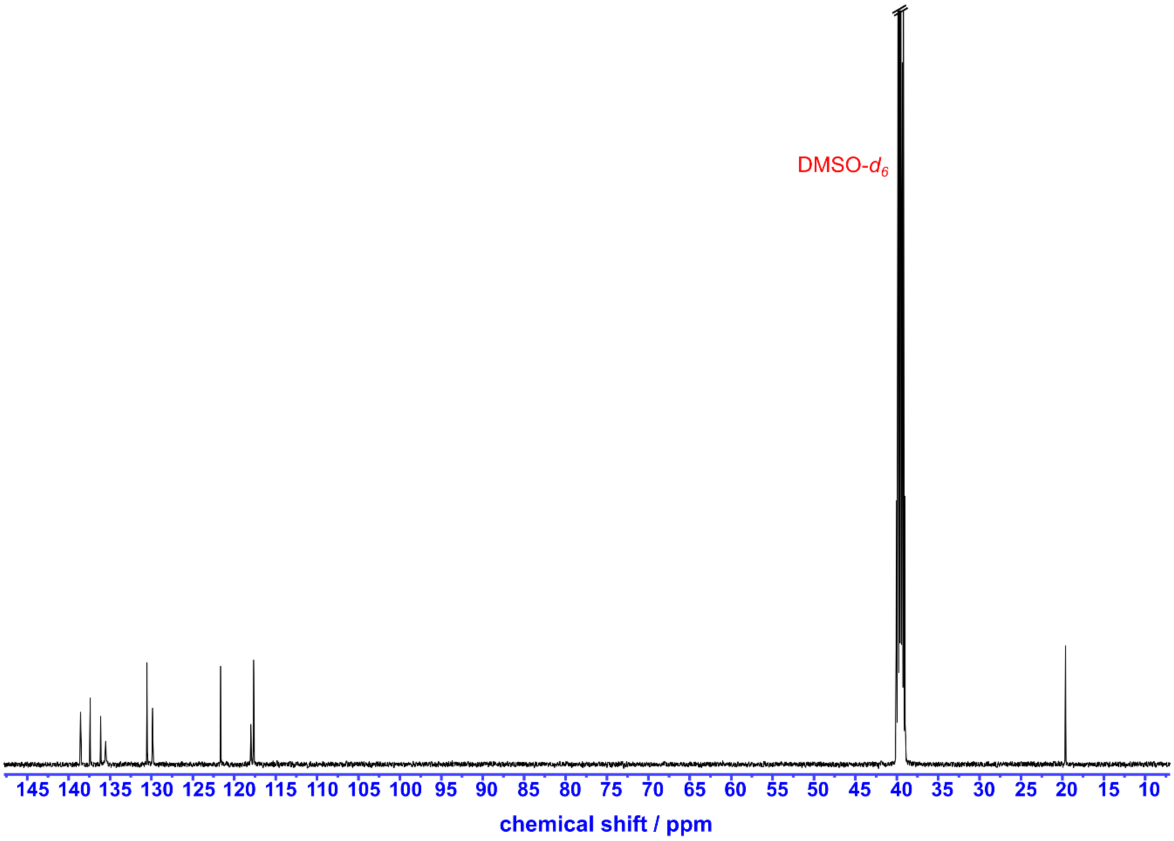

Figure S10: $^{13}C[^{1}H]$ NMR spectrum of **L^3**
(125 MHz, 298 K, DMSO-d_6)

S4.3 Synthesis of 1,1'-(3,3'-dimethyl-[1,1'-biphenyl]-4,4'-diyl)bis(1H-imidazole) L⁴

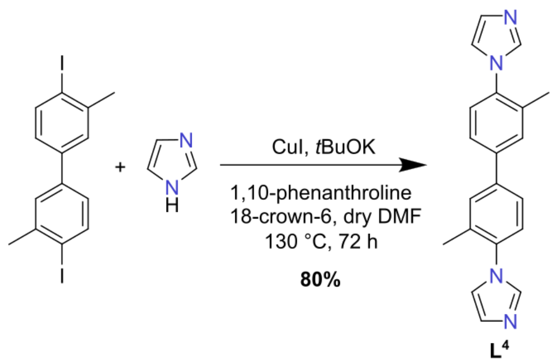

4,4'-Diiodo-3,3'-dimethyl-1,1'-biphenyl was synthesized following a literature protocol.[3] A flame-dried 100 mL double-necked flask was charged with CuI (29 mg, 0.15 mmol), 1,10-phenanthroline (54 mg, 0.30 mmol) and dry DMF (10 mL). After degassing the solution was heated at 120 °C for 5 min. To this mixture 4,4'-diiodo-3,3'-dimethyl-1,1'-biphenyl (651 mg, 1.50 mmol), imidazole (817 mg, 12.0 mmol), potassium *tert*-butoxide (1680 mg, 15.0 mmol), and two crystals of 18-crown-6 were added and the mixture heated at 130 °C for 72 h under N_2 flow. The resultant thick brown slurry was added to ice-cold water (50 mL) and extracted with CH_2Cl_2 (50 mL × 3). The organics were collected over Na_2SO_4, filtered, and the solvent removed *in vacuo*. The crude mixture was purified using silica gel column chromatography with THF:CH_2Cl_2 (1:4) as the eluent. Finally, the solid was washed Et_2O (3 × 15 mL) to remove excess 18-crown-6 to afford ligand **L^4** as a white crystalline solid (376 mg, 1.20 mmol, 80%).

^{1}H NMR (500 MHz, DMSO-d_6) δ_H: 7.88 (s, 2H), 7.79 (d, *J* = 1.7 Hz, 2H), 7.68 (dd, *J* = 8.1 Hz, 1.7 Hz, 2H), 7.45 (s, 2H), 7.41 (d, *J* = 8.1 Hz, 2H), 7.12 (s, 2H), 2.25 (s, 6H).

^{13}C NMR (125 MHz, DMSO-d_6) δ_C: 139.1, 137.7, 136.2, 133.8, 129.5, 128.8, 126.8, 125.2, 120.9, 17.6.

ESI-MS (ESI, MeCN), *m/z*: calculated for $[M+H]^+$, $[C_{20}H_{19}N_4]^+$, 315.1604, found 315.1608.

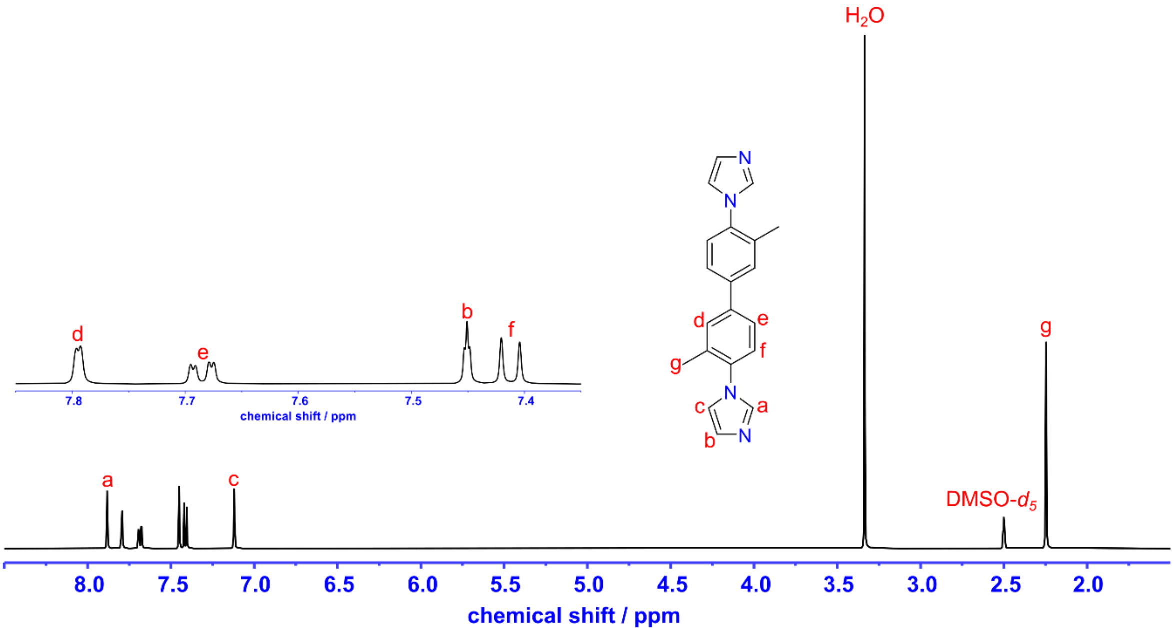

Figure S11: ^{1}H NMR spectrum of **L^4**
(500 MHz, 298 K, DMSO-d_6)

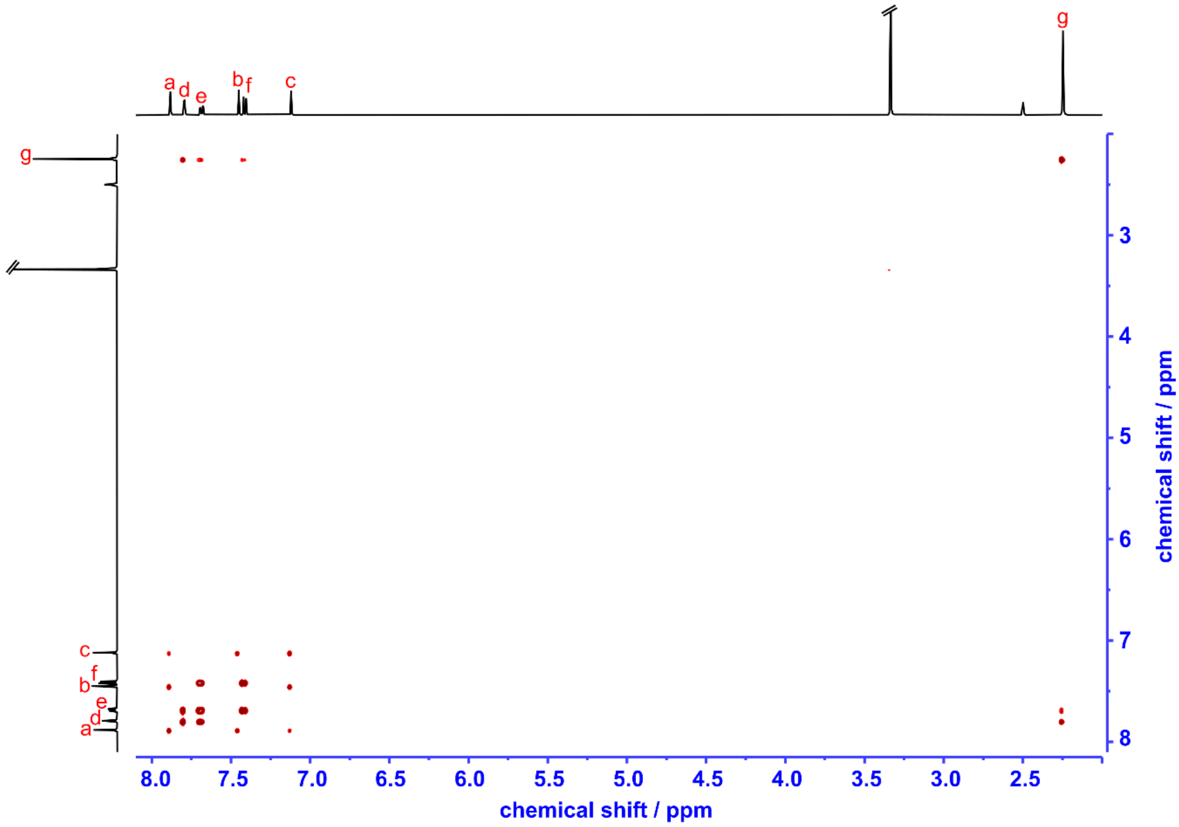

Figure S12: ^{1}H-^{1}H COSY spectrum of **L^4**
(500 MHz, 298 K, DMSO-d_6)

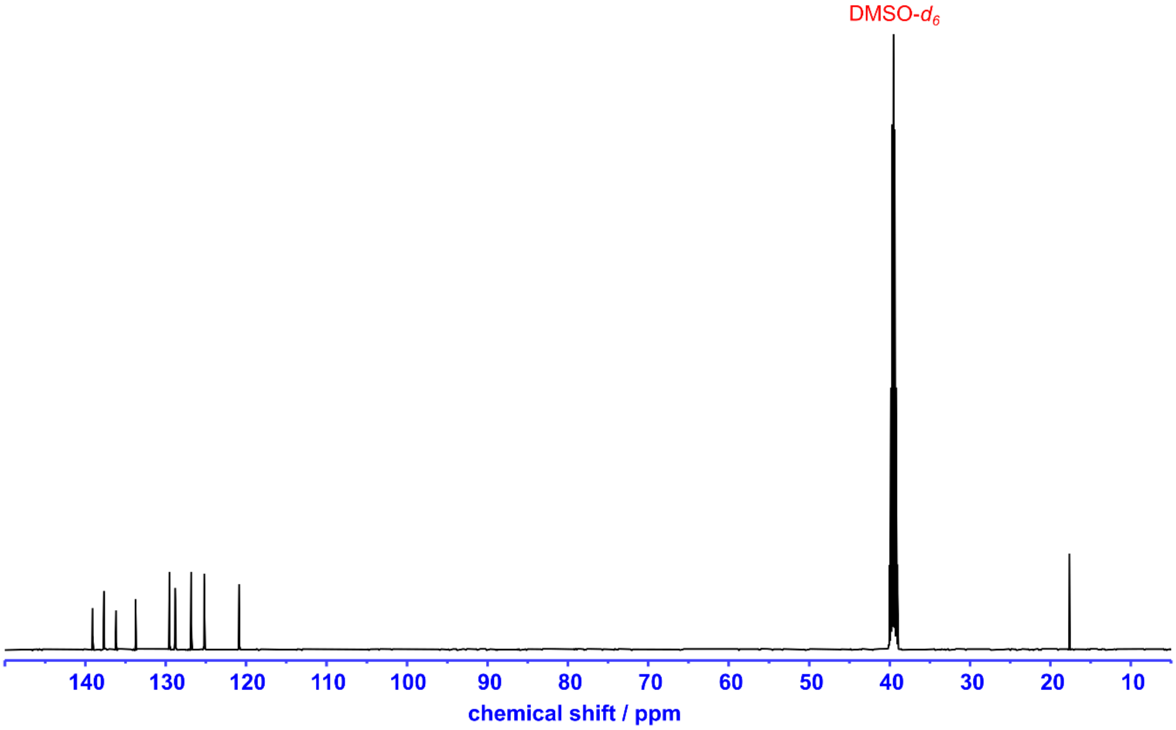

Figure S13: $^{13}C[^1H]$ NMR spectrum of **L^4**
(125 MHz, 298 K, DMSO-d_6)

S5 Synthesis and characterisation of metal-organic cages

S5.1 Self-assembly of $Pd_8L^1_{16}$ square antiprism 1

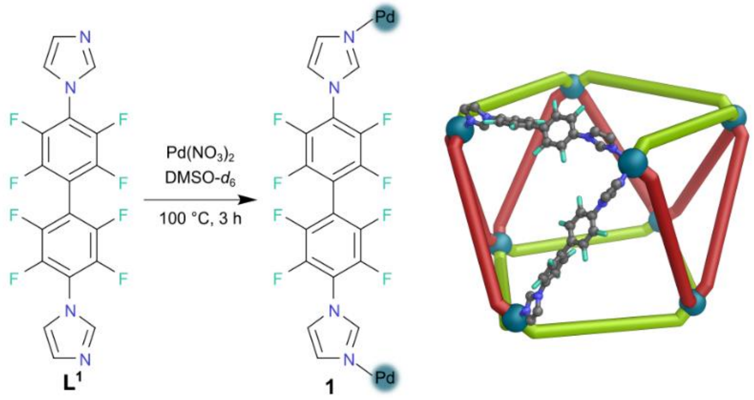

1,1'-(Perfluoro-[1,1'-biphenyl]-4,4'-diyl)bis(1H-imidazole) **L¹** (4.30 mg, 0.0100 mmol) and $Pd(NO_3)_2.2H_2O$ (1.33 mg, 0.00500 mmol) were placed in a 1-dram (4 mL) vial and DMSO-d_6 (0.6 mL) was added. This mixture was then heated at 100 °C for 3 h. The resulting reaction mixture was centrifuged (3000 rpm, 5 min) and the liquid decanted to obtain a pale-yellow solution of cage **1**. Multinuclear NMR experiments were performed on this sample after leaving it to sit for 2 h at room temperature.

^{1}H NMR (500 MHz, DMSO-d_6) δ_H: 9.14 (s, 16H, a), 8.77 (s, 16H, a’), 8.02 (s, 16H, b’), 7.94 (s, 16H, c’), 7.91 (s, 16H, b), 7.61 (s, 16H, c).

^{19}F NMR (376 MHz, DMSO-d_6) δ_F: −139.75 (br, 32F), −140.04 (br, 32F), −148.27 (br, 32F), −148.82 (br, 32F).

^{13}C NMR[^{19}F] (101 MHz, DMSO-d_6) δ_C: 143.64, 143.60, 141.6, 141.5, 140.10 (d, br. J = 223.5 Hz, 2 signals), 128.92 (d, br. J = 199.8 Hz, 2 signals), 123.11 (d, br. J = 204.4 Hz, 2 signals), 118.03 (2 signals), 105.84, 105.81.

ESI-MS (ESI, DMSO), *m/z*: [**1**$(NO_3)_{12}$]$^{4+}$ calculated 2119.7106, found 2119.7069; [**1**$(NO_3)_{11}$]$^{5+}$ calculated 1683.3709, found 1683.3710; [**1**$(NO_3)_{10}$]$^{6+}$ calculated 1392.4778, found 1392.4775; [**1**$(NO_3)_9$]$^{7+}$ calculated 1184.6970, found 1184.6984; [**1**$(NO_3)_8$]$^{8+}$ calculated 1028.8614, found 1028.8605; [**1**$(NO_3)_7$]$^{9+}$ calculated 907.6559, found 1028.6546.

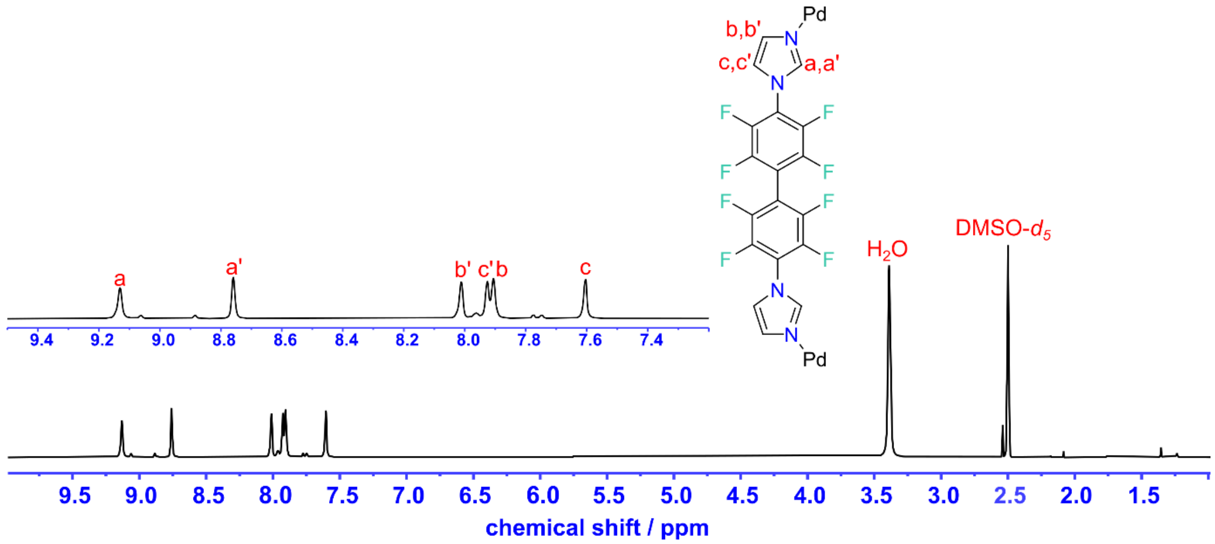

Figure S14: ^{1}H NMR spectrum of cage **1**
(500 MHz, 298 K, DMSO-d_6)

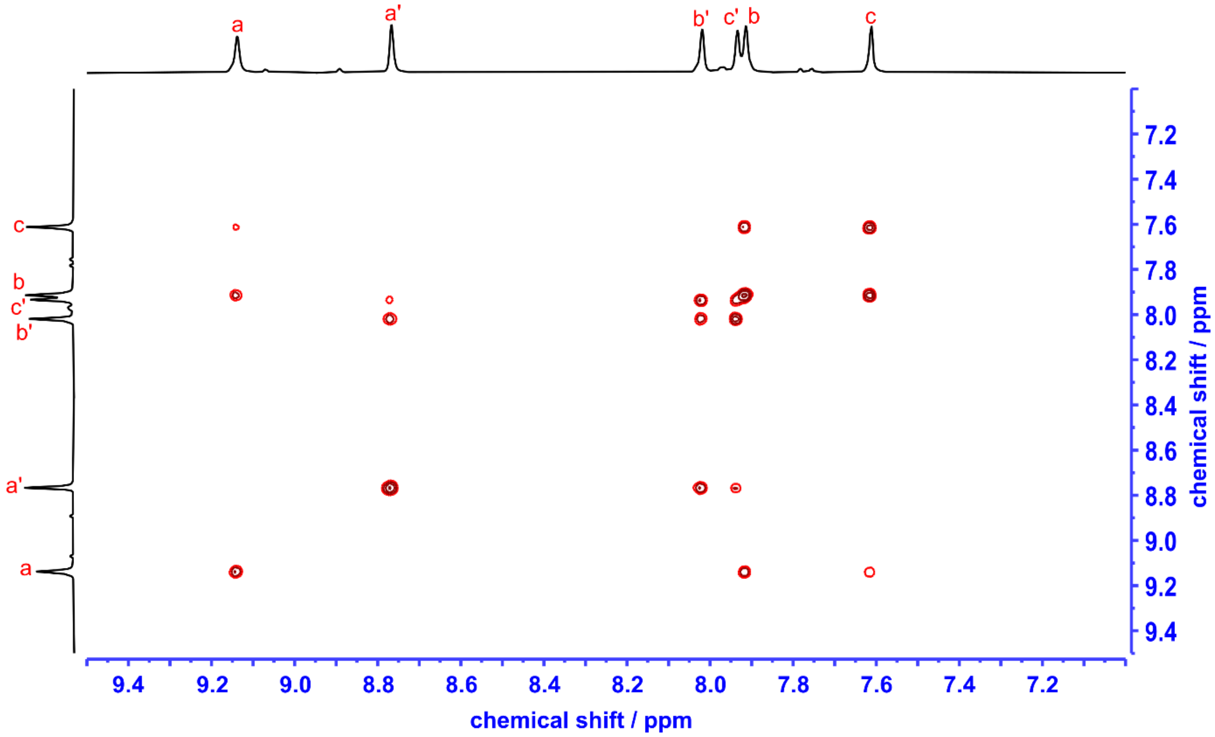

Figure S15: ^{1}H-^{1}H COSY spectrum of cage **1**
(500 MHz, 298 K, DMSO-d_6)

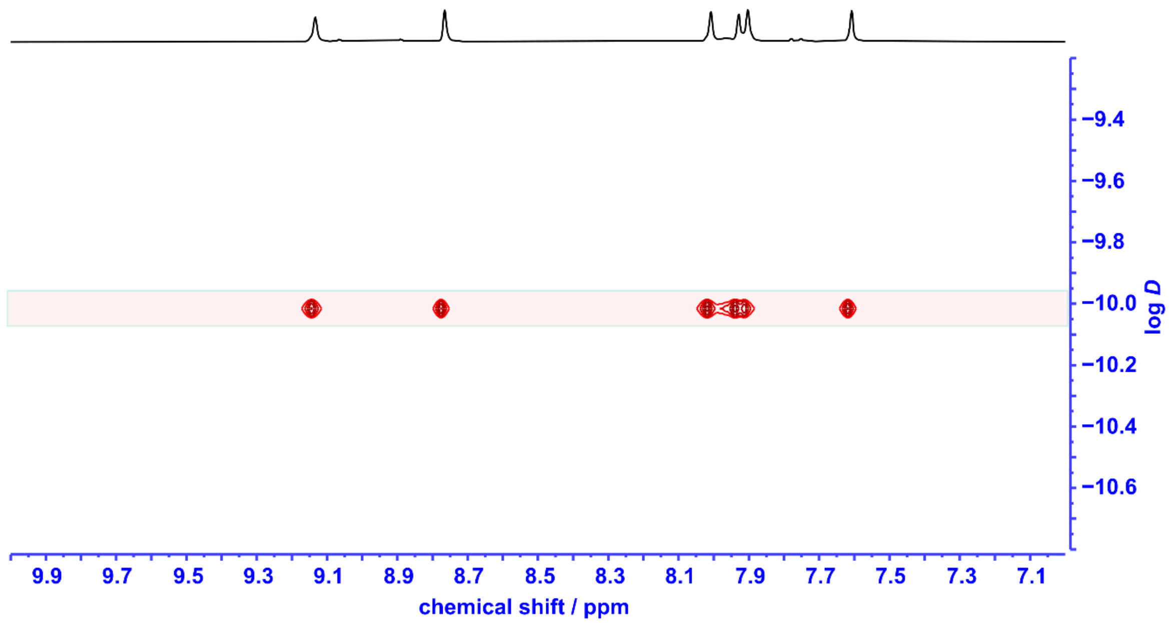

Figure S16: ^{1}H DOSY spectrum of cage **1**
(500 MHz, 298 K, DMSO-d_6)

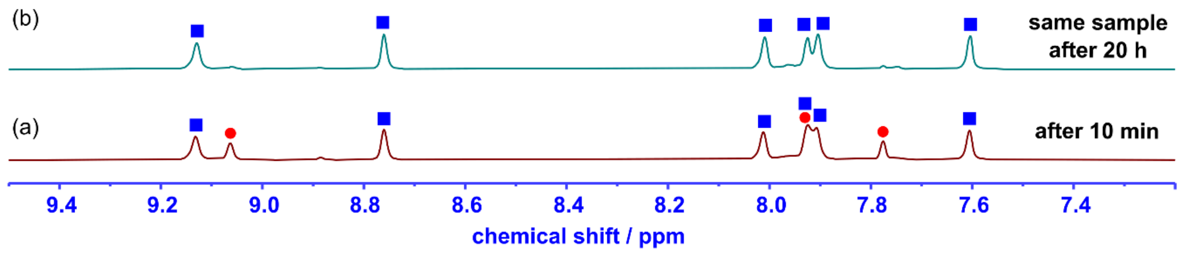

Figure S17: ^{1}H NMR spectrum of cage **1**: (a) recorded after 10 min of reaction time (maroon); (b) same sample recorded after 20 h (teal); Blue squares denote the peaks corresponding to Pd_8L_{16} square antiprism and red circles denote an intermediate (presumed Pd_6L_{12} octahedron).
(400 MHz, 298 K, DMSO-d_6)

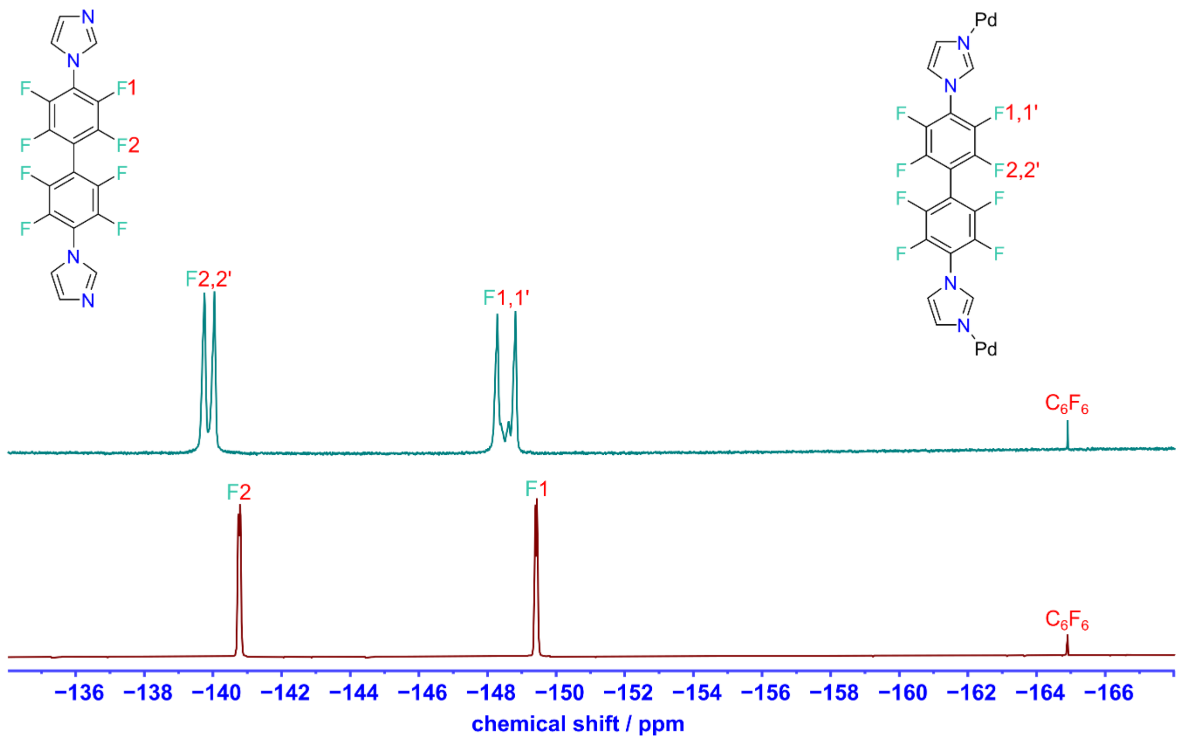

Figure S18: ^{19}F stacked plot NMR spectra of ligand **L^1** (maroon) and cage **1** (teal) (376 MHz, 298 K, DMSO-d_6)

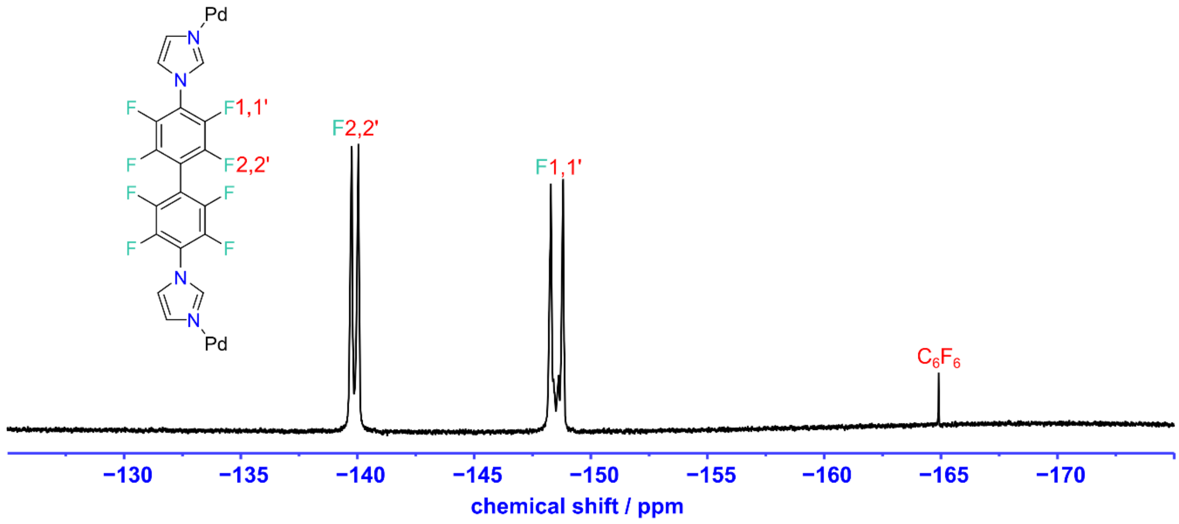

Figure S19: ^{19}F NMR spectrum of cage **1** (376 MHz, 298 K, DMSO-d_6)

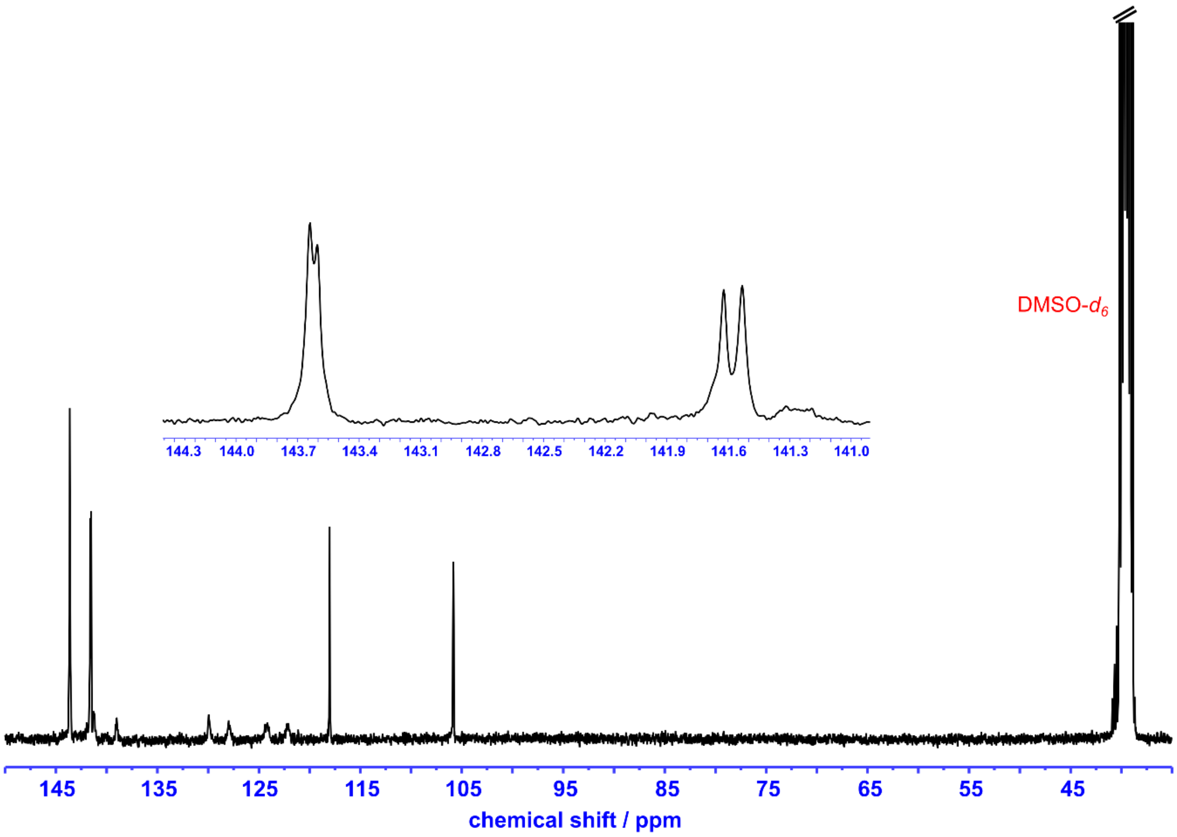

Figure S20: $^{13}C[^{19}F]$ NMR spectrum of **1**. (Inset) Enlarged partial spectrum showing the doubling of the ^{13}C signals
(101 MHz, 298 K, DMSO-d_6)

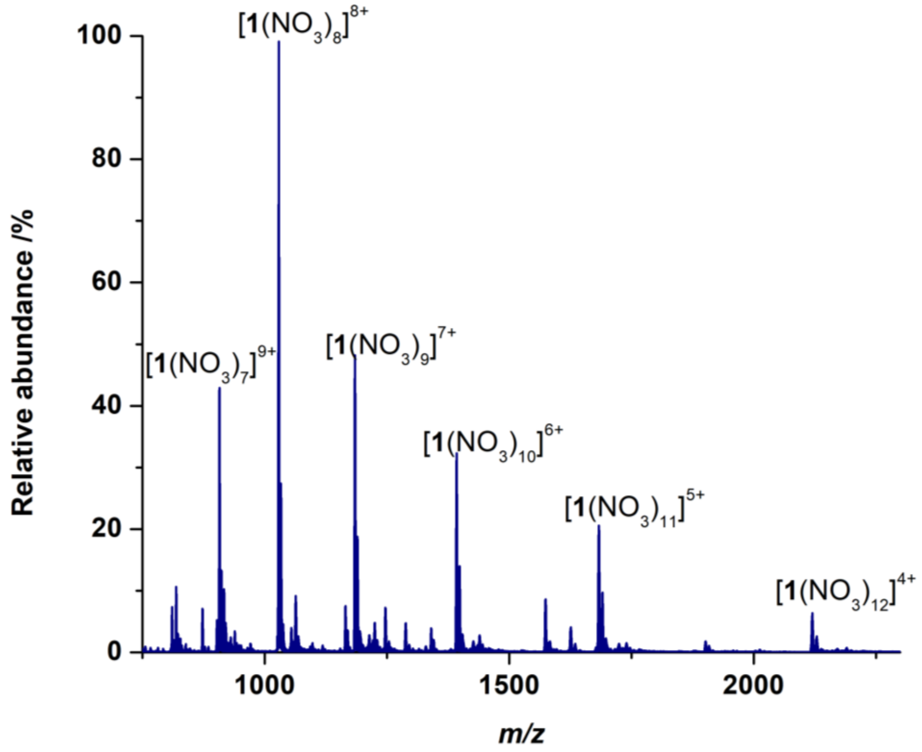

Figure S21: ESI-MS of cage **1**.

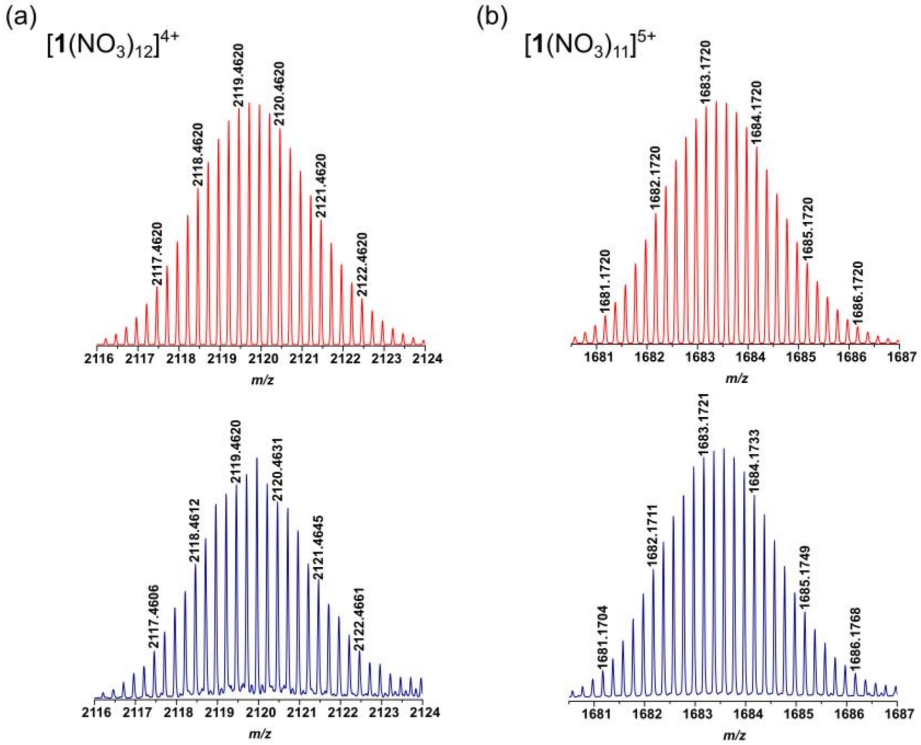

Figure S22: Isotopic distribution of selected peaks from cage **1** showing match between predicted (red) and experimentally observed (blue) patterns (a) $[\mathbf{1}(NO_3)_{12}]^{4+}$, (b) $[\mathbf{1}(NO_3)_{11}]^{5+}$.

S5.2 Self-assembly of $Pd_6L^2{}_{12}$ octahedron 2

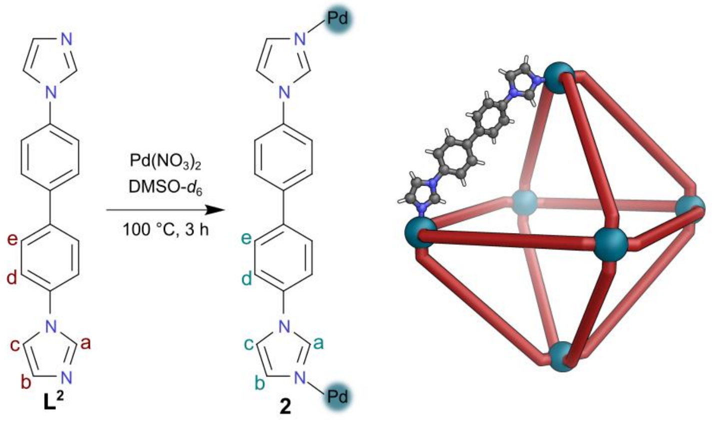

4,4'-Di(1H-imidazol-1-yl)-1,1'-biphenyl **L²** (2.86 mg, 0.0100 mmol) and $Pd(NO_3)_2.2H_2O$ (1.33 mg, 0.00500 mmol) were added in a 1-dram (4 mL) vial and DMSO-d_6 (0.6 mL) was added. This mixture was then heated at 100 °C for 3 h. The resulting reaction mixture was centrifuged (3000 rpm, 5 min) and the liquid decanted to obtain an orange solution of cage **2**.

^{1}H NMR (500 MHz, DMSO-d_6) δ_H: 9.21 (s, 24H, a), 8.08 (s, 24H, b), 7.97 (d, J = 8.5 Hz, 48H, d), 7.86 (d, J = 8.5 Hz, 48H, e), 7.60 (s, 24H, c).

^{13}C[^{1}H] NMR (125 MHz, DMSO-d_6) δ_C: 138.4, 137.0, 135.2, 128.9, 128.1, 121.5, 119.9.

ESI-MS (ESI, DMSO), *m/z*: [**2**$(NO_3)_9$]$^{3+}$ calculated 1543.9287, found 1543.9284; [**2**$(NO_3)_8$]$^{4+}$ calculated 1142.4496, found 1142.4517; [**2**$(NO_3)_7$]$^{5+}$ calculated 901.5621, found 901.5621; [**2**$(NO_3)_6$]$^{6+}$ calculated 740.9705, found 740.9693; [**2**$(NO_3)_5$]$^{7+}$ calculated 626.2621, found 626.2613; [**2**$(NO_3)_4$]$^{8+}$ calculated 540.2309, found 540.2307.

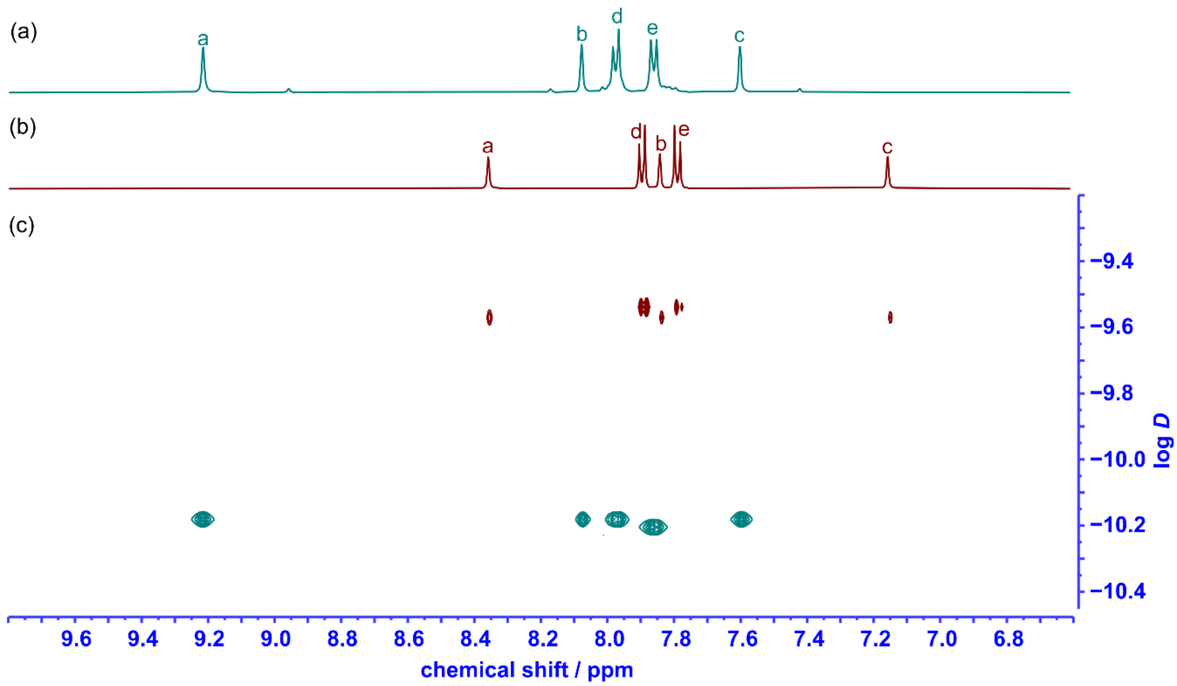

Figure S23: Stacked ^{1}H NMR spectra of: (a) cage **2** (teal) and (b) ligand $\mathbf{L^2}$ (maroon). (c) Overlaid DOSY spectra of cage **2** (teal) and ligand $\mathbf{L^2}$ (maroon). (Note: the DOSY spectra for cage **2** and ligand $\mathbf{L^2}$ were recorded on a different NMR probe to the rest of the DOSY experiments, due to a change in probe on the machine; the probe for these spectra shown here gave slightly higher values of diffusion coefficients than the other probe.) (500 MHz, 298 K, DMSO-d_6)

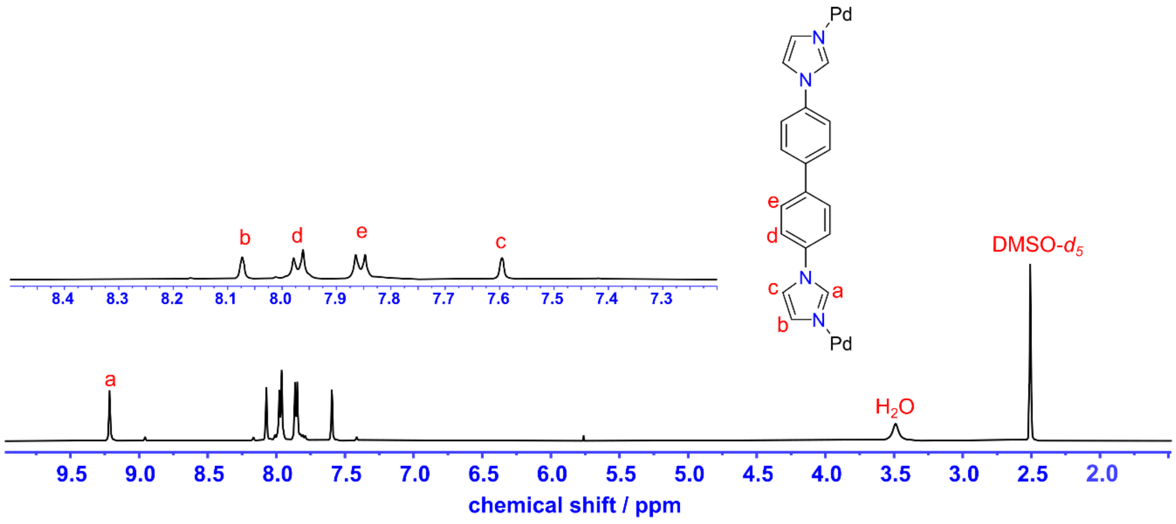

Figure S24: ^{1}H NMR spectrum of cage **2** (500 MHz, 298 K, DMSO-d_6)

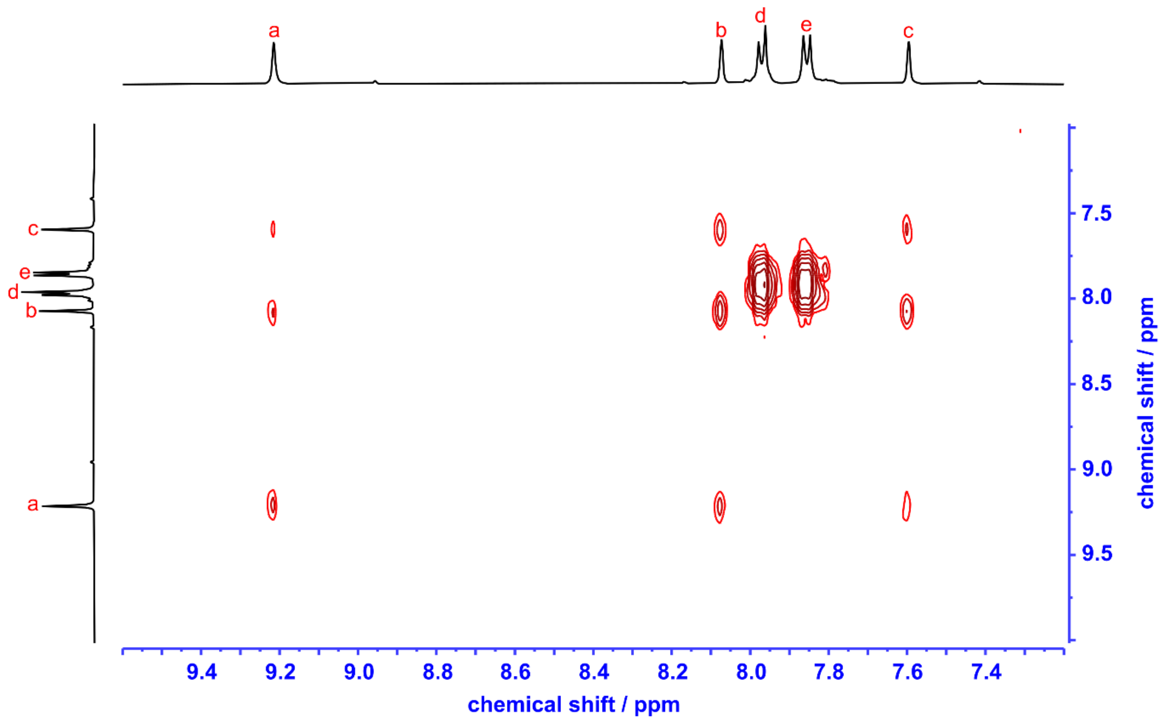

Figure S25: ^{1}H-^{1}H COSY spectrum of cage **2**
(500 MHz, 298 K, DMSO-d_6)

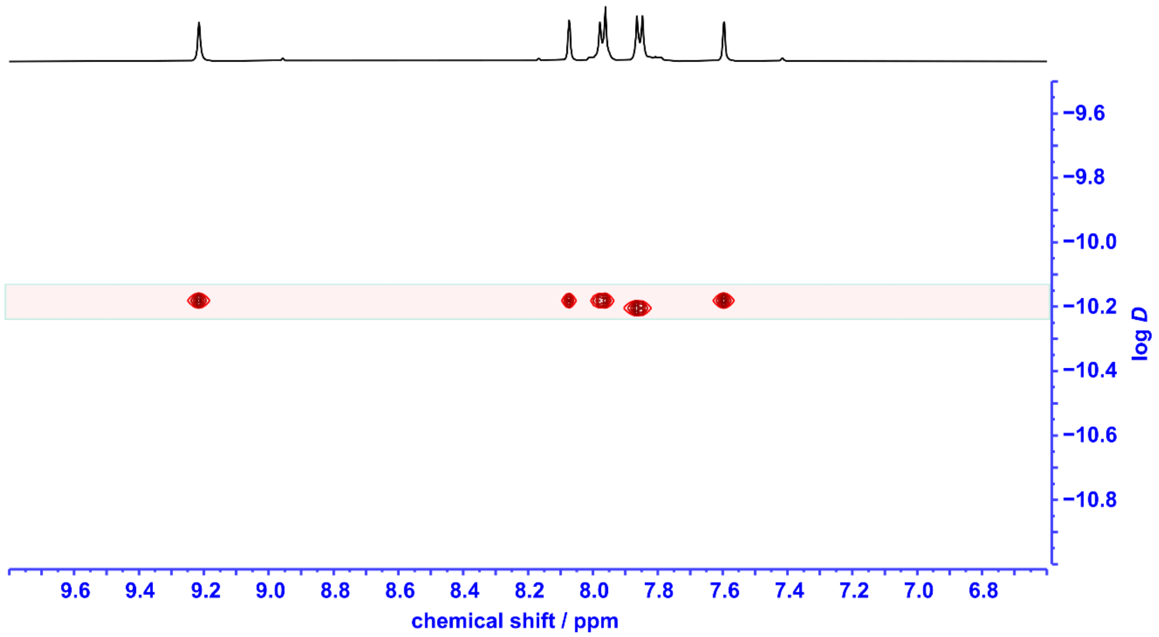

Figure S26: ^{1}H DOSY spectrum of cage **2**. (Note: the DOSY spectra for cage **2** was recorded on a different NMR probe to the rest of the DOSY experiments, due to a change in probe on the machine; the probe for this spectrum shown here gave slightly higher values of diffusion coefficients than the other probe.)
(500 MHz, 298 K, DMSO-d_6)

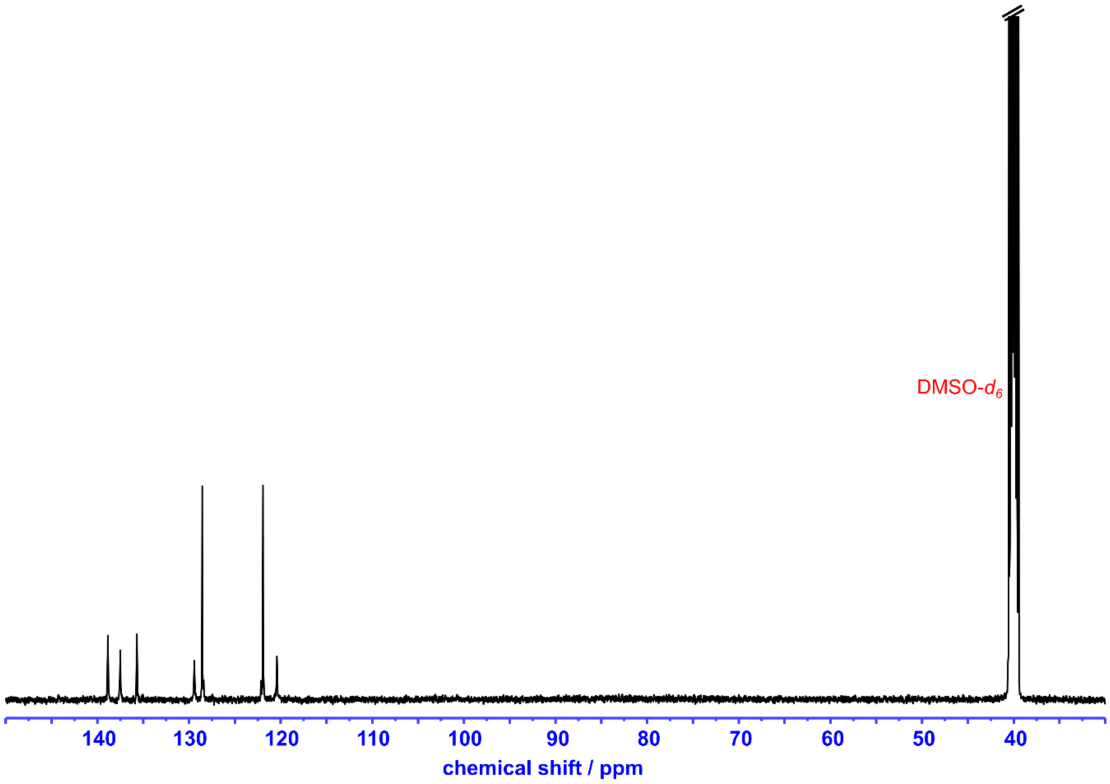

Figure S27: $^{13}C[^{1}H]$ NMR spectrum of cage **2**
(125 MHz, 298 K, DMSO-d_6)

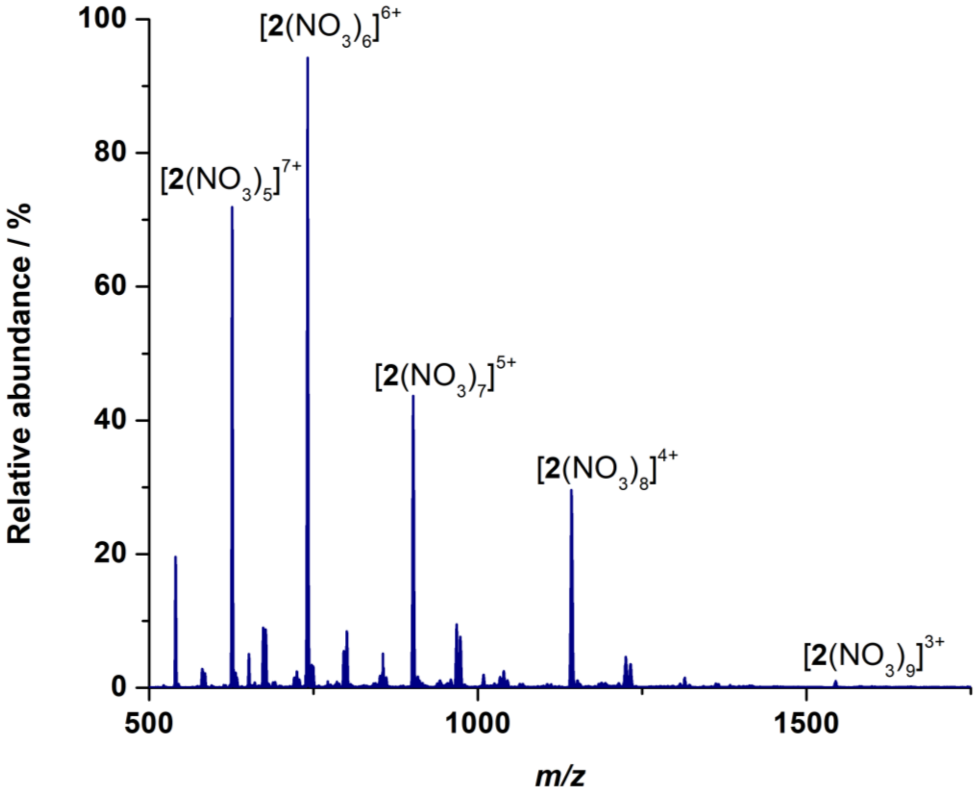

Figure S28: ESI-MS of cage **2**

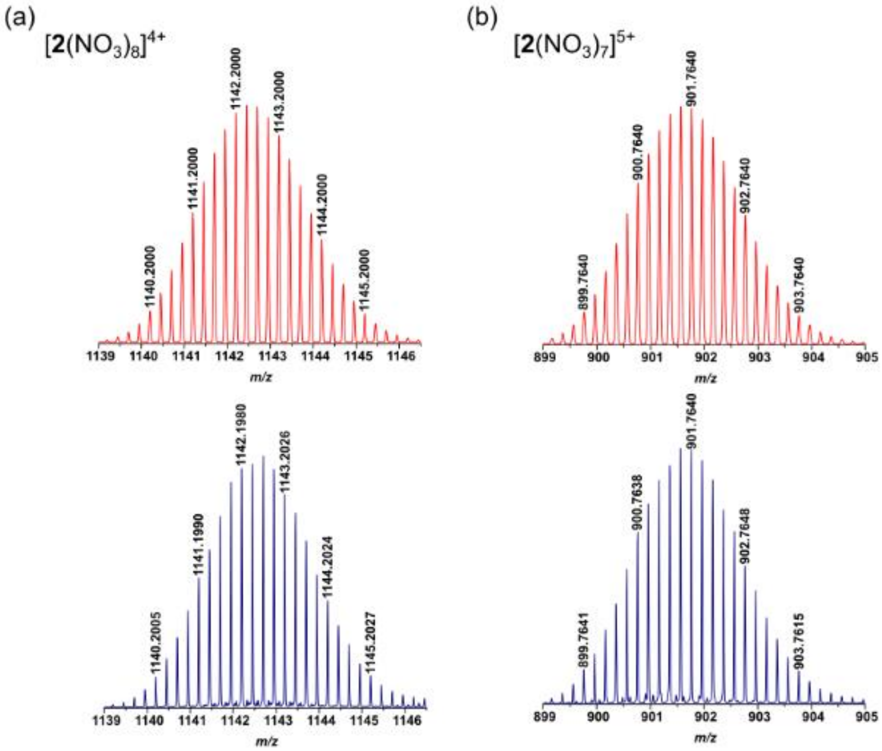

Figure S29: Isotopic distribution of selected peaks from cage **2** showing match between predicted (red) and experimentally observed (blue) patterns
(a) [**2**$(NO_3)_8$]$^{4+}$, (b) [**2**$(NO_3)_7$]$^{5+}$.

S5.3 Self-assembly of $Pd_6L^3_{12}$ octahedron 3

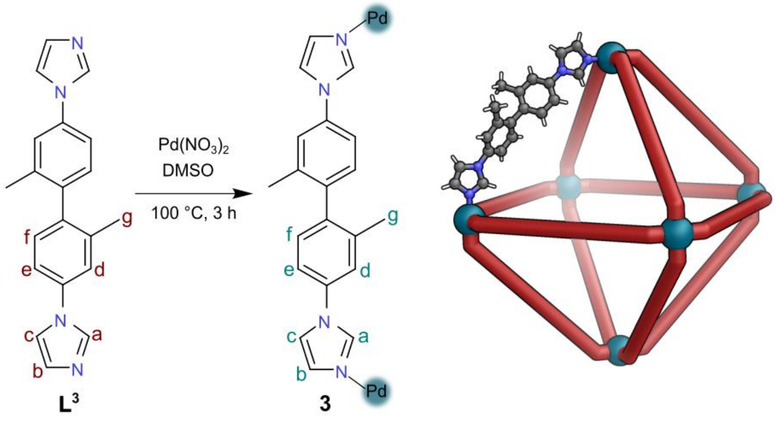

1,1'-(2,2'-Dimethyl-[1,1'-biphenyl]-4,4'-diyl)bis(1H-imidazole) **L^3** (6.30 mg, 0.0200 mmol) and $Pd(NO_3)_2.2H_2O$ (2.66 mg, 0.0100 mmol) were added to a 1-dram (4 mL) vial and 1.2 mL DMSO was added. This mixture was then heated at 100 °C for 3 h. The resulting solution was centrifuged and poured onto 25 mL of EtOAc and then left to sit for 18 h. The mixture was then centrifuged (300 rpm, 10 min), the solid decanted, and then washed with acetone (10 mL). The solid was dried *in vacuo* before being redissolved in DMSO-d_6 (0.6 mL) by sonication and gentle heating. This mixture was then further centrifuged (3000 rpm, 5 min) and the clear solution of cage **3** was decanted and used for NMR experiments.

^{1}H NMR (500 MHz, DMSO-d_6) δ_H: 9.01 (br. s, 24H, a), 8.02 (s, 24H, b), 7.66 (s, 24H, d), 7.59 (br. m, 48H, c,e), 7.22 (br. m, 24H, f), 2.05 (s, 72H, g).

^{13}C[^{1}H] NMR (125 MHz, DMSO-d_6) δ_C: 139.9, 137.5 (2 signals), 135.0, 130.5, 128.9, 122.9, 120.2, 119.1, 19.6.

ESI-MS (ESI, DMSO), *m/z*: [**3**$(NO_3)_{13}$]$^{3+}$ calculated 1656.3873, found 1656.3916; [**3**$(NO_3)_{12}$]$^{4+}$ calculated 1226.7935, found 1226.7954; [**3**$(NO_3)_{11}$]$^{5+}$ calculated 969.0372, found 969.0414; [**3**$(NO_3)_{10}$]$^{6+}$ calculated 797.1997, found 797.2013; [**3**$(NO_3)_9$]$^{7+}$ calculated 674.4586, found 674.4597.

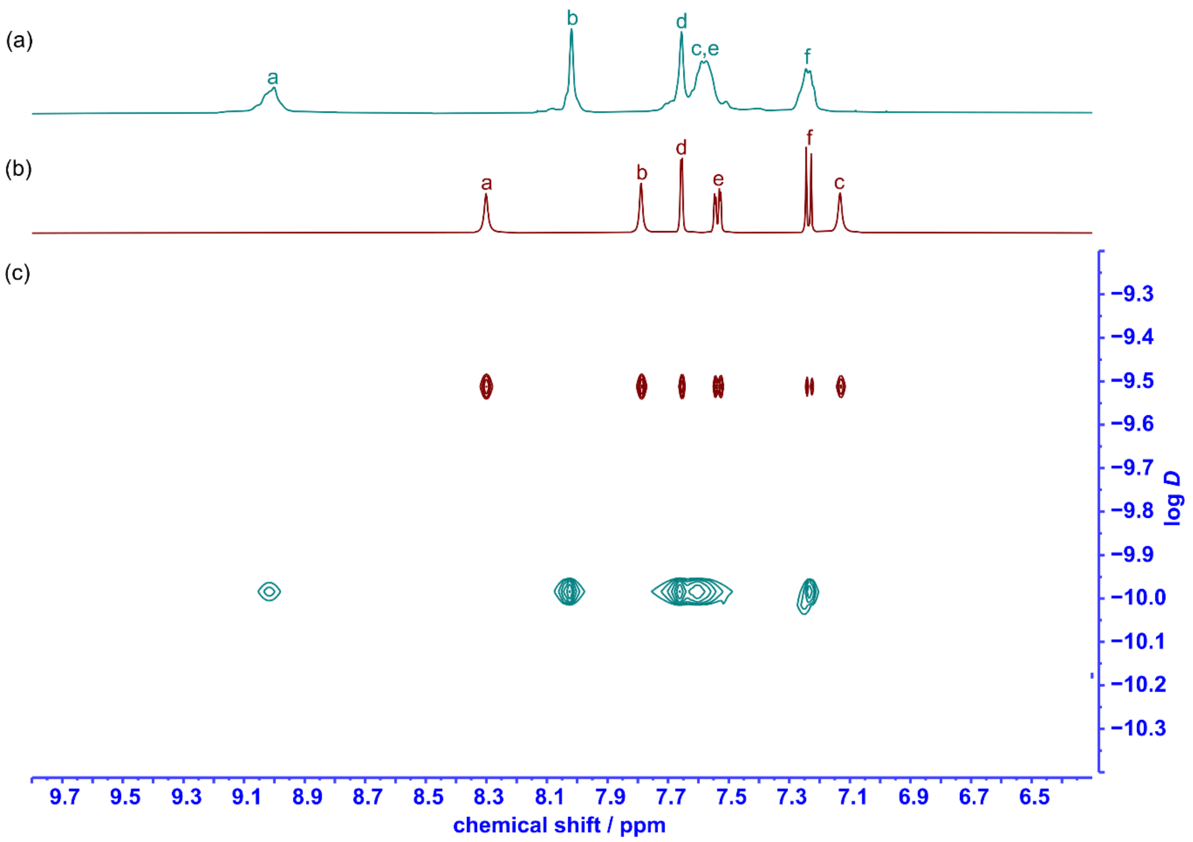

Figure S30: Stacked ^{1}H NMR spectra of: (a) cage **3** (teal) and (b) ligand $\mathbf{L^3}$ (maroon). (c) Overlaid DOSY spectra of cage **3** (teal) and ligand $\mathbf{L^3}$ (maroon) (500 MHz, 298 K, DMSO-d_6)

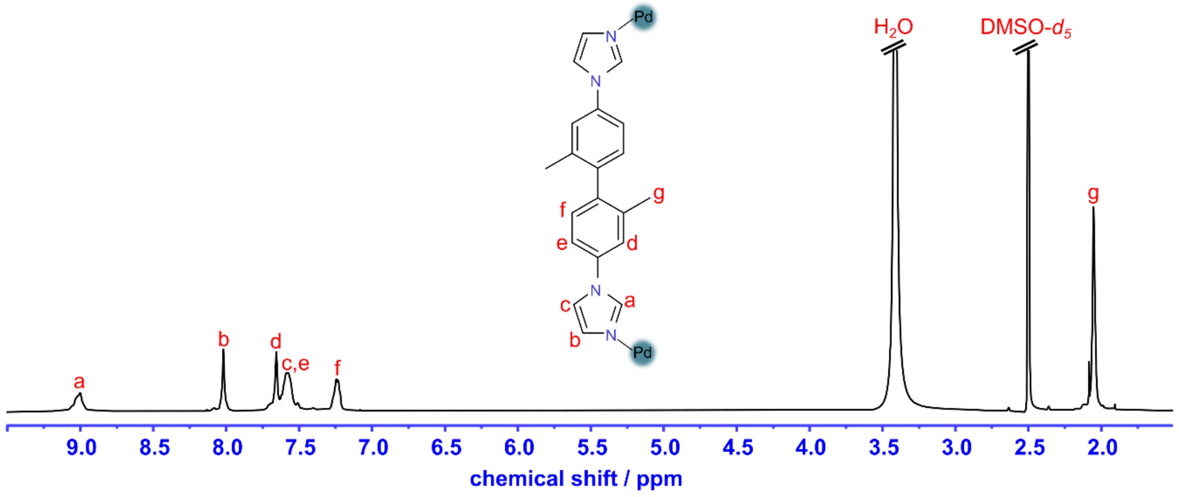

Figure S31: ^{1}H NMR spectrum of cage **3** (500 MHz, 298 K, DMSO-d_6)

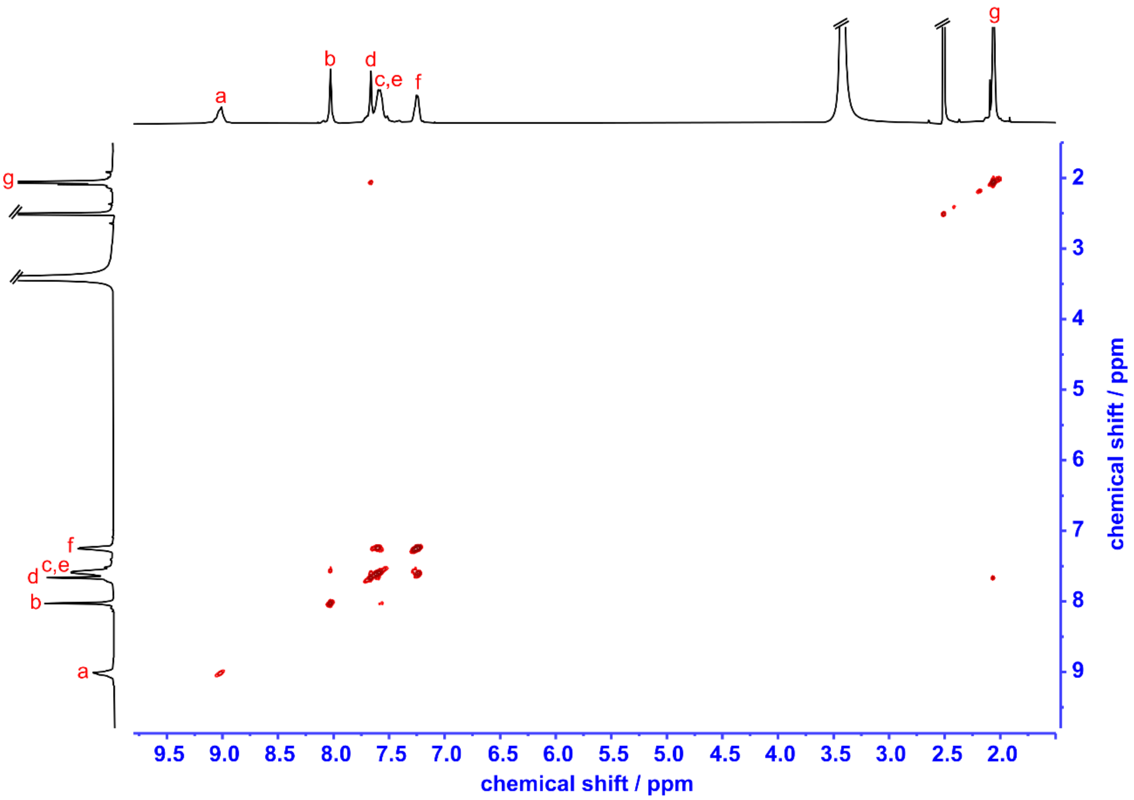

Figure S32: ^{1}H-^{1}H COSY spectrum of cage **3**
(500 MHz, 298 K, DMSO-d_6)

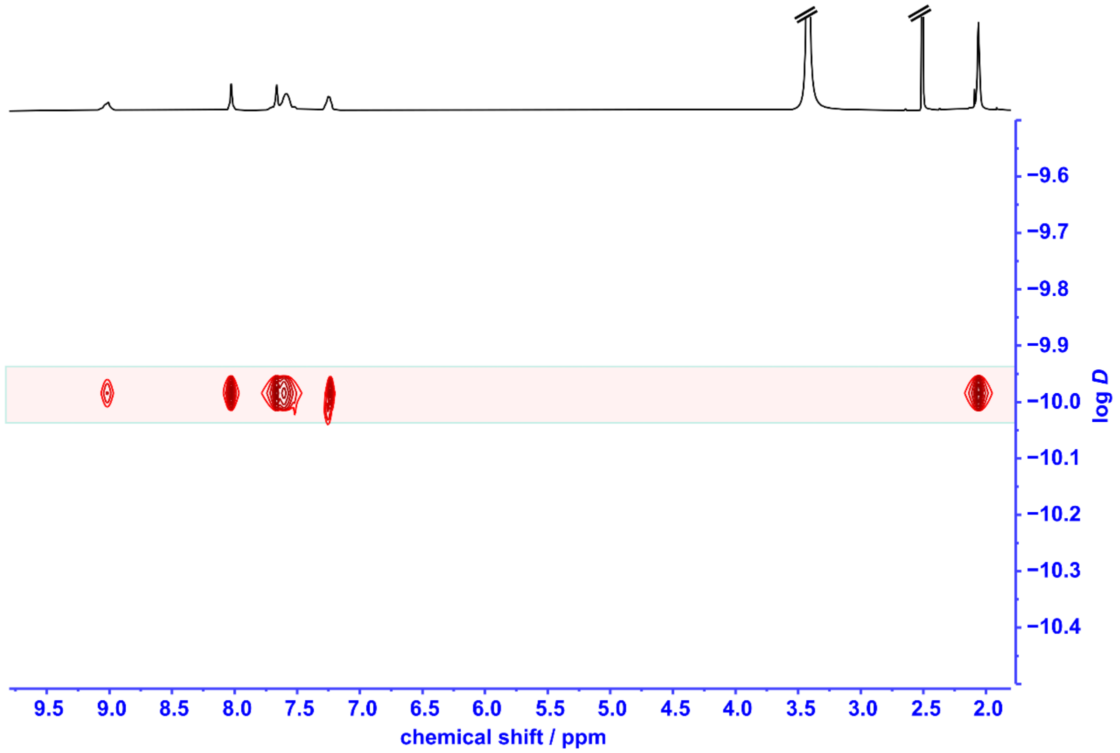

Figure S33: ^{1}H DOSY spectrum of cage **3**
(500 MHz, 298 K, DMSO-d_6)

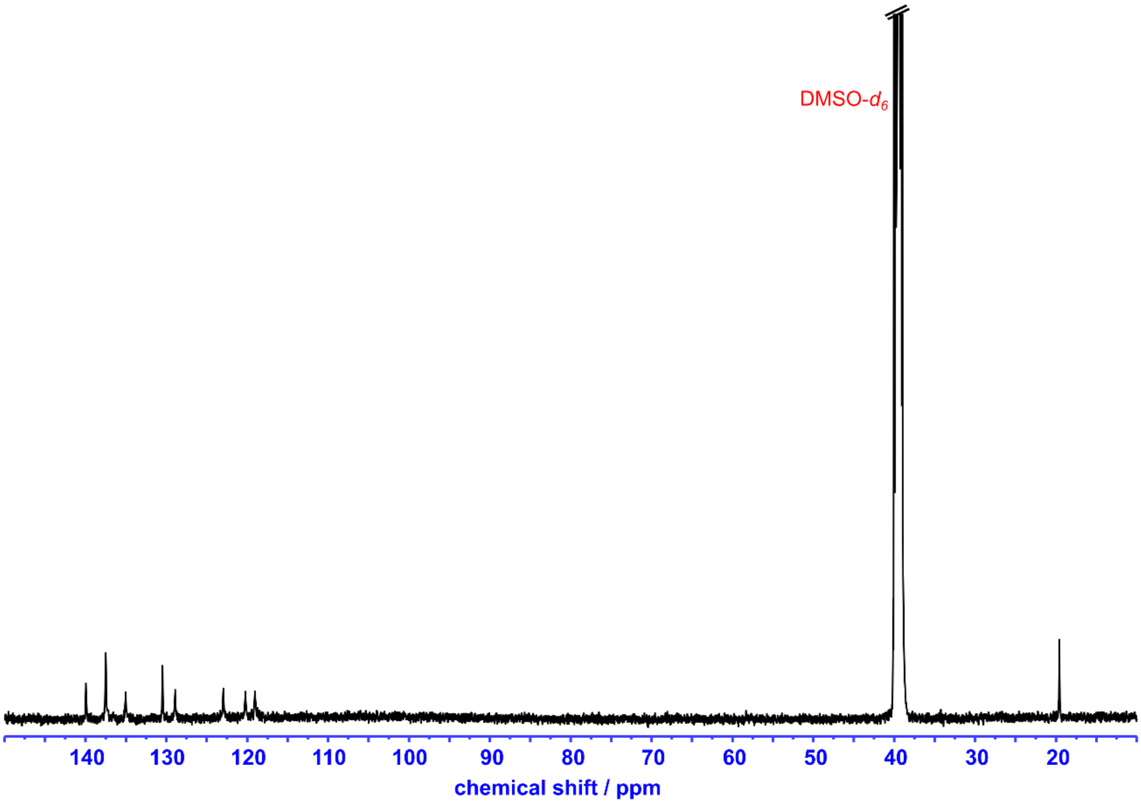

Figure S34: ^{13}C NMR spectrum of cage **3**
(125 MHz, 298 K, DMSO-d_6)

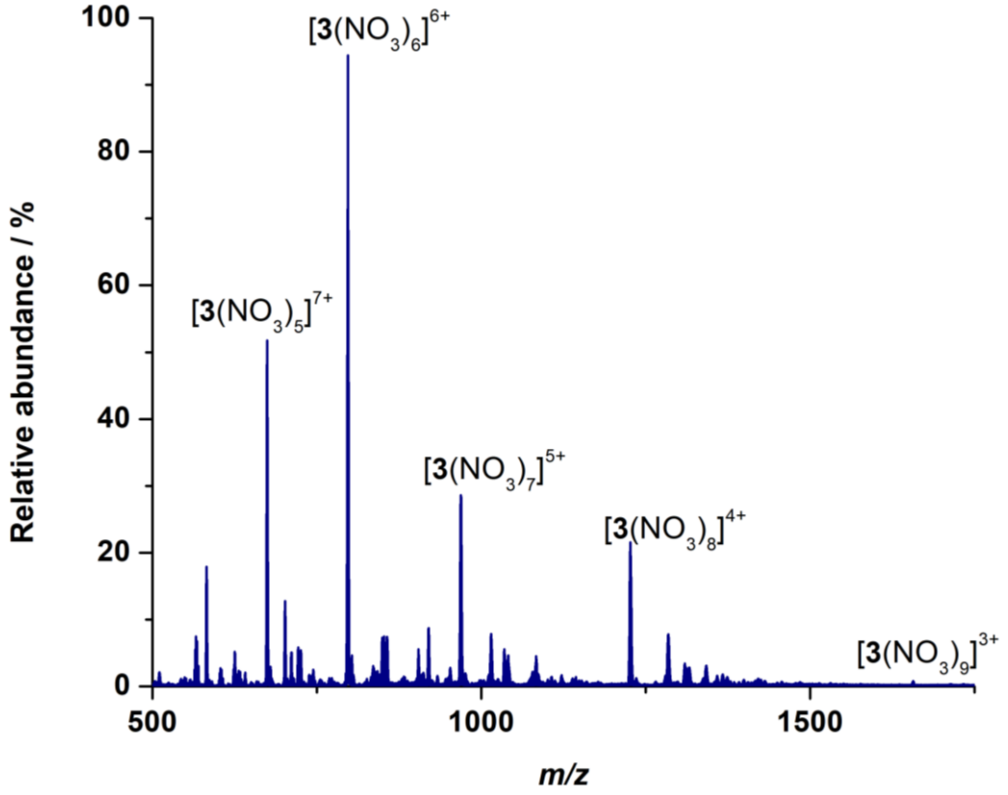

Figure S35: ESI-MS of cage **3**

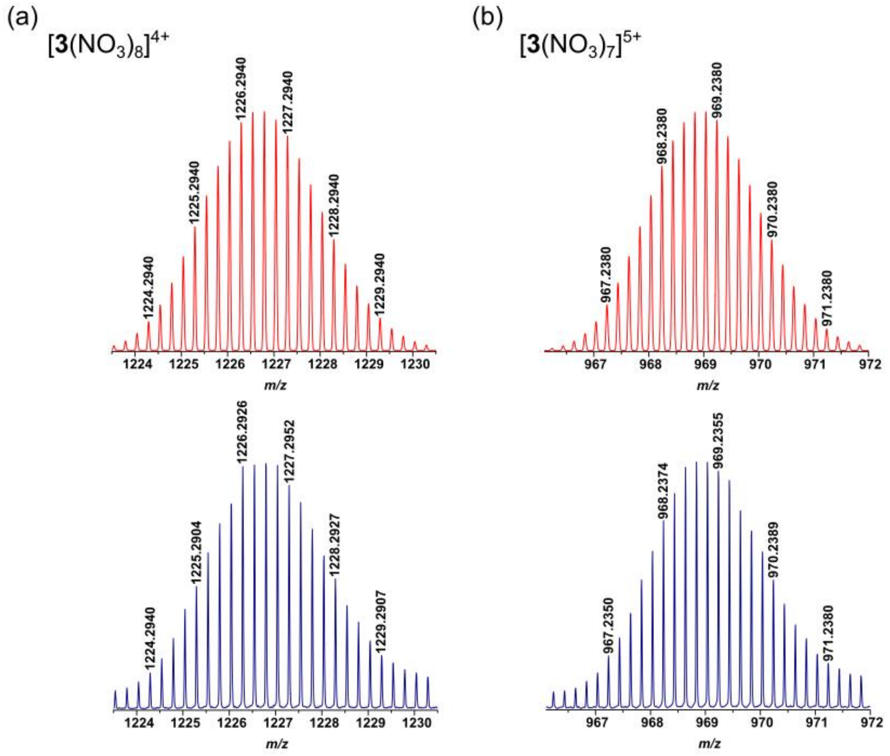

Figure S36: Isotopic distribution of selected peaks from cage **3** showing match between predicted (red) and experimentally (blue) observed patterns
(a) [**3**$(NO_3)_8$]$^{4+}$, (b) [**3**$(NO_3)_7$]$^{5+}$

S5.4 Self-assembly of $Pd_6L^4{}_{12}$ octahedron 4

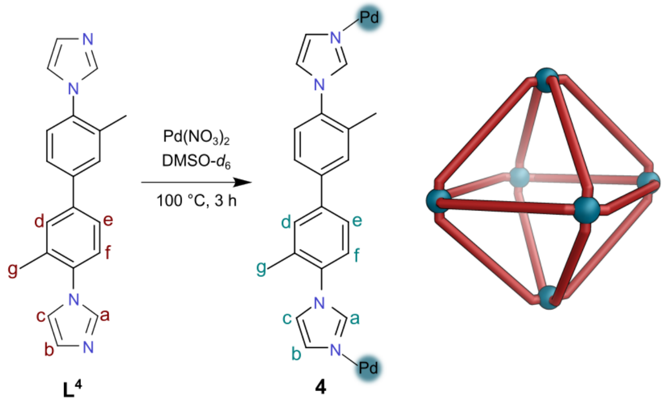

1,1'-(3,3'-Dimethyl-[1,1'-biphenyl]-4,4'-diyl)bis(1H-imidazole) **L^4** (3.14 mg, 0.0100 mmol) and $Pd(NO_3)_2.2H_2O$ (1.33 mg, 0.0050 mmol) were added to a 1-dram (4 mL) vial and DMSO-d_6 (0.6 mL) was added. This mixture was then heated at 100 °C for 3 h. The resulting reaction mixture was centrifuged (3000 rpm, 5 min) and the liquid decanted to obtain a yellow solution of cage **4**.

^{1}H NMR (500 MHz, DMSO-d_6) δ_H: 8.62 (s, 24H, a), 7.83 (s, 24H, d), 7.79 (s, 24H, b), 7.71 (d, J = 8.6 Hz, 24H, e), 7.56 (s, 24H, c), 7.44 (d, J = 8.6 Hz, 24H, f), 2.08 (s, 72H, g).

^{13}C[^{1}H] NMR (125 MHz, DMSO-d_6) δ_C: 139.9, 139.4, 135.0, 134.1, 129.4, 128.1, 127.2, 125.3, 122,7, 17.0.

ESI-MS (ESI, DMSO), *m/z*: [**4**$(NO_3)_9]^{3+}$ calculated 1656.3873, found 1656.3926; [**4**$(NO_3)_8]^{4+}$ calculated 1226.7935, found 1226.7960; [**4**$(NO_3)_7]^{5+}$ calculated 969.0372, found 969.0389; [**4**$(NO_3)_6]^{6+}$ calculated 797.1997, found 797.1987.

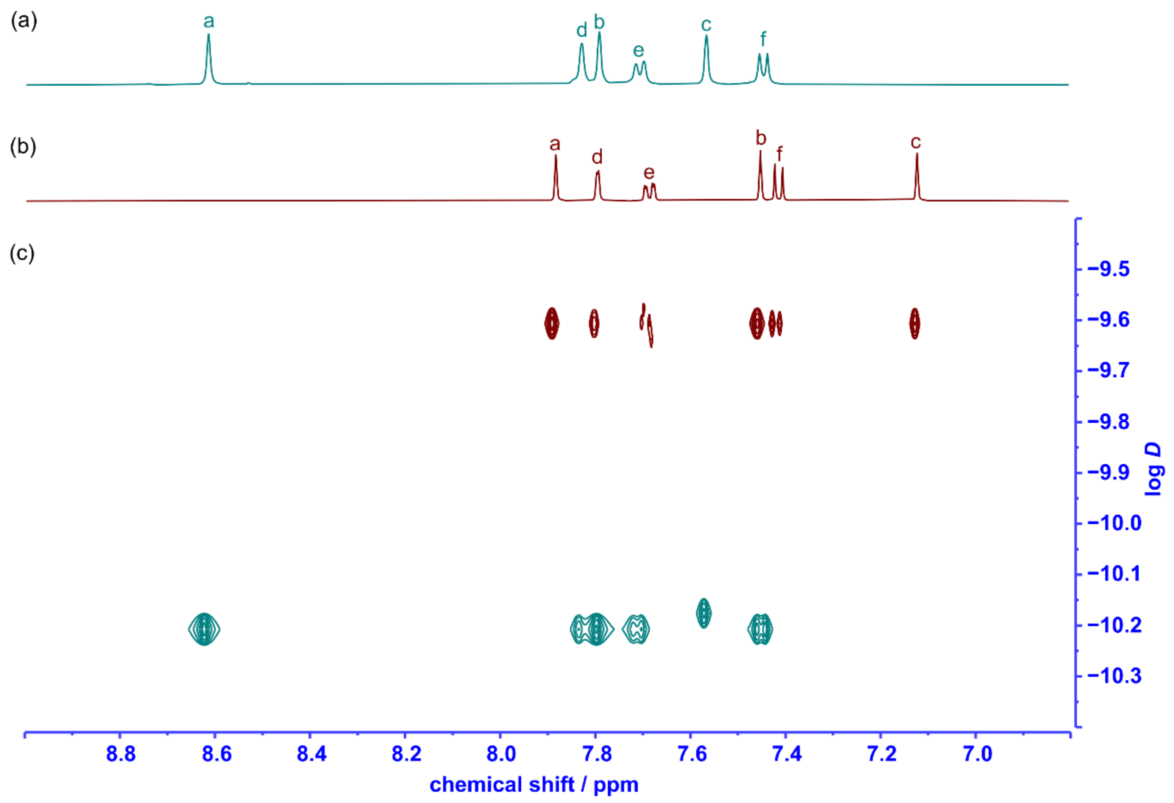

Figure S37: Stacked ^{1}H NMR spectra of: (a) cage **4** (teal) and (b) ligand **L⁴** (maroon). (c) Overlaid DOSY spectra of cage **4** (teal) and ligand **L⁴** (maroon) (500 MHz, 298 K, DMSO-d_6)

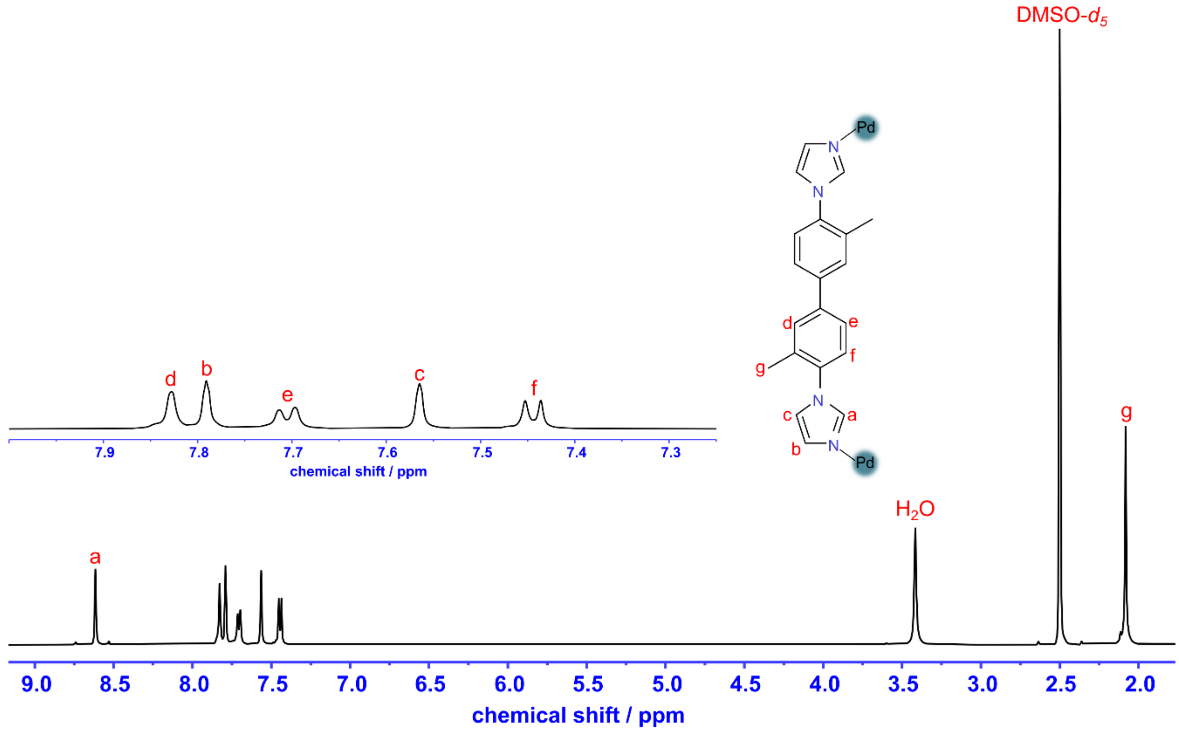

Figure S38: ^{1}H NMR spectrum of cage **4** (500 MHz, 298 K, DMSO-d_6)

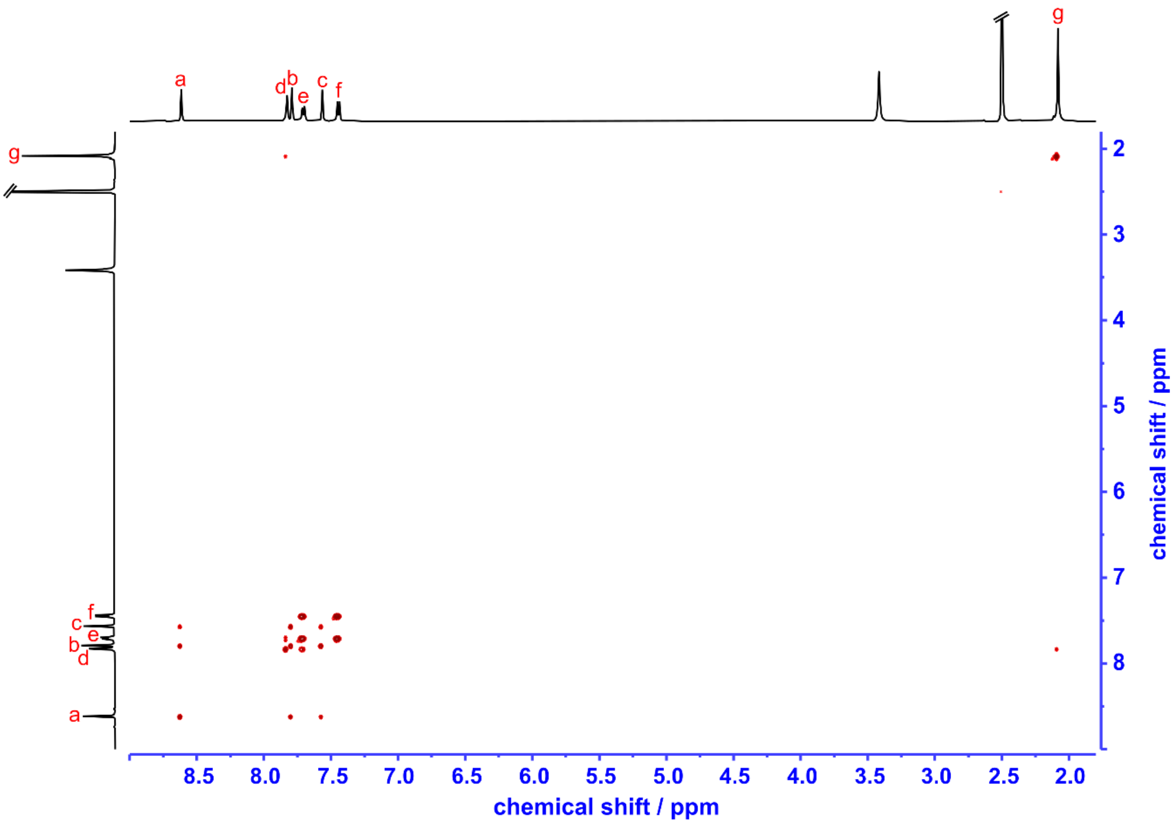

Figure S39: ^{1}H-^{1}H COSY spectrum of cage **4**
(500 MHz, 298 K, DMSO-d_6)

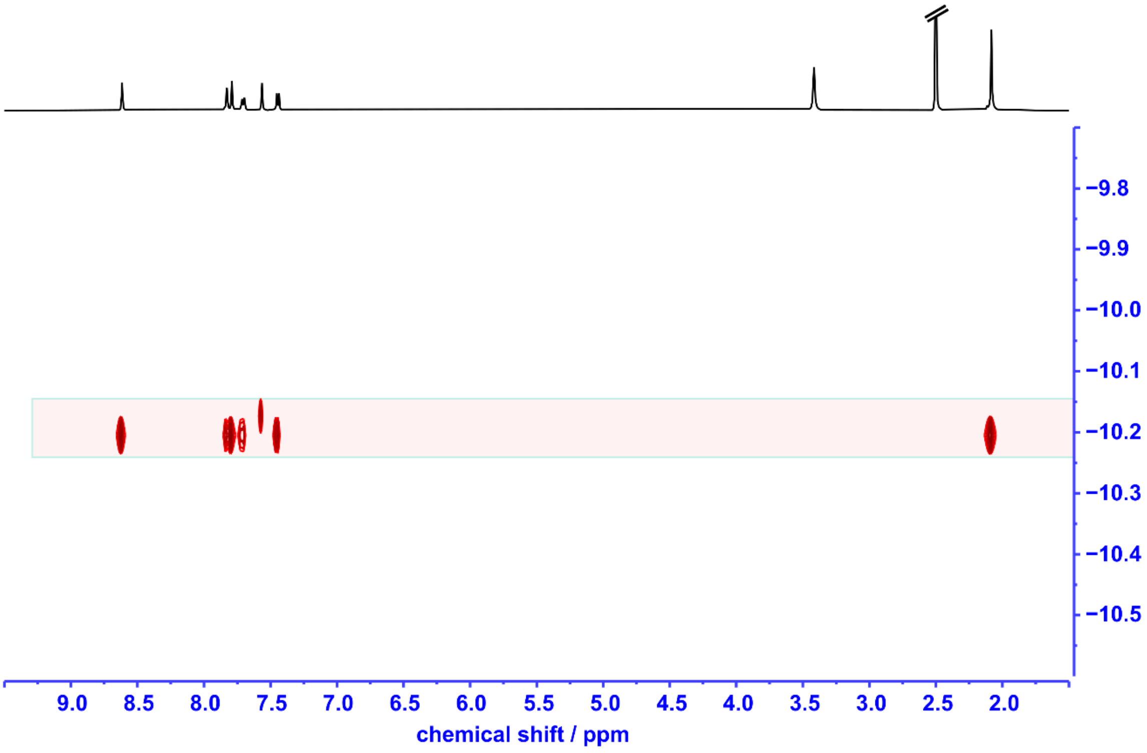

Figure S40: ^{1}H DOSY spectrum of cage **4**
(500 MHz, 298 K, DMSO-d_6)

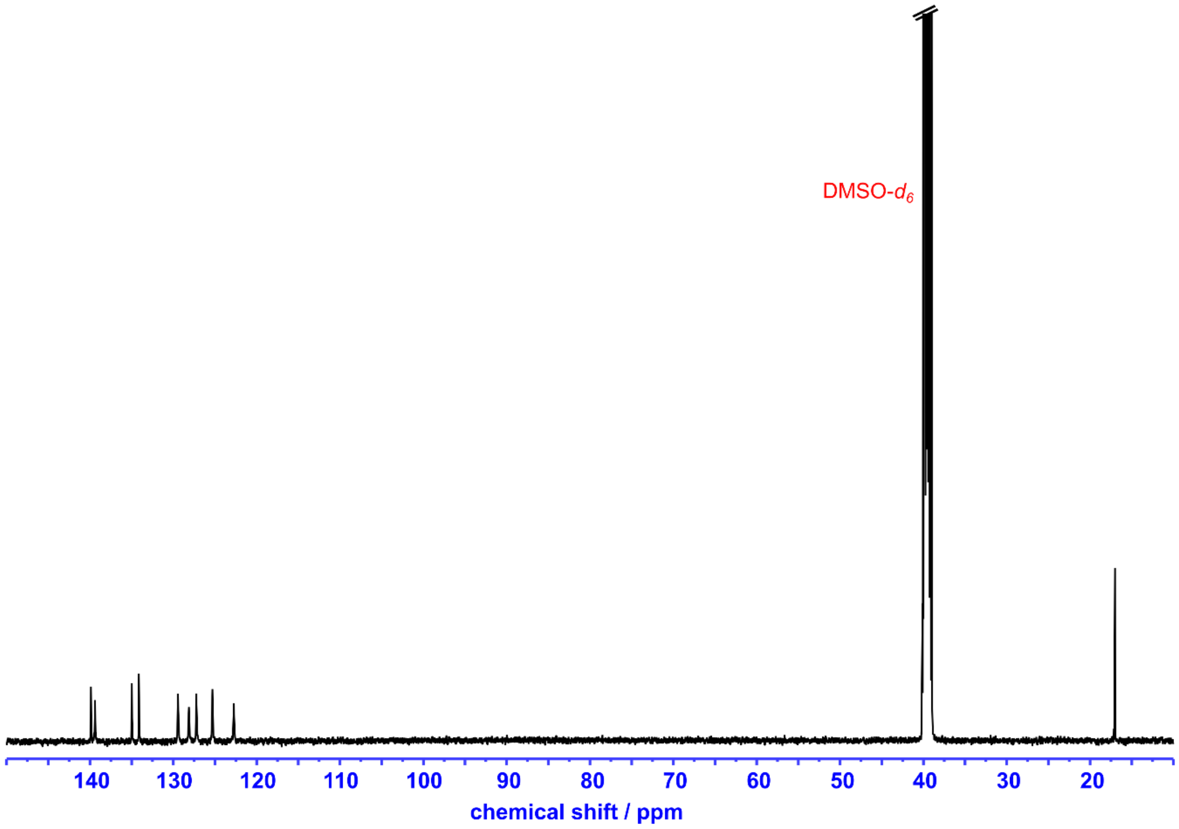

Figure S41: ^{13}C NMR spectrum of cage **4**
(125 MHz, 298 K, DMSO-d_6)

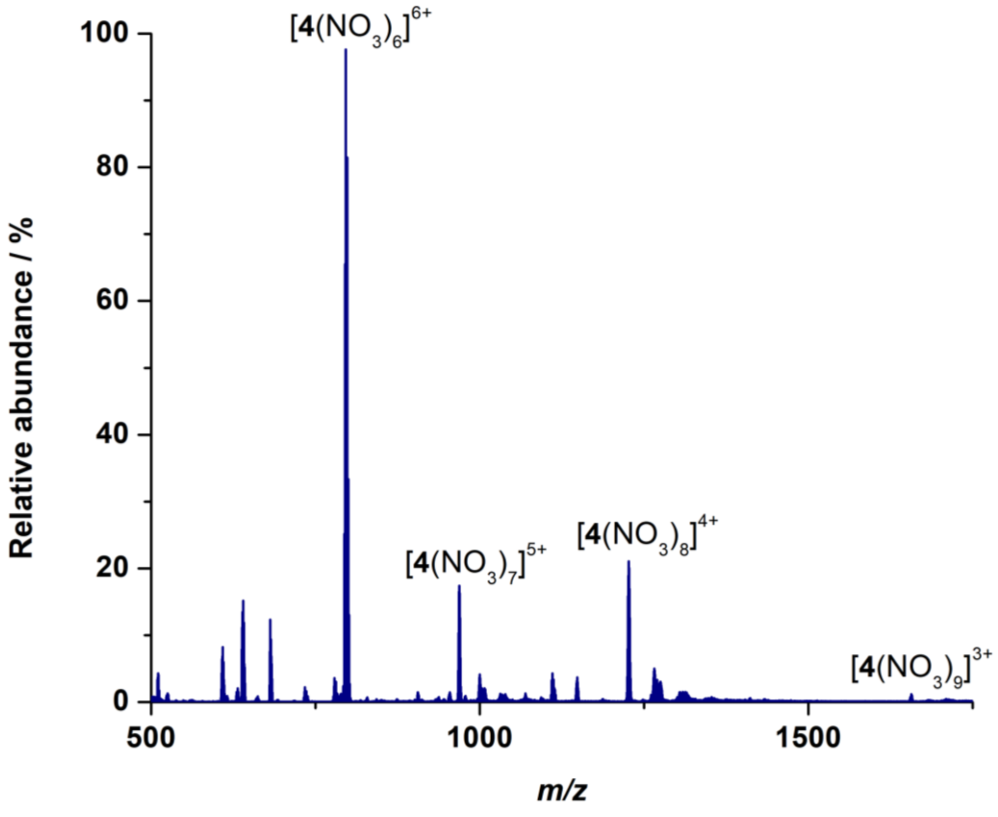

Figure S42: ESI-MS of cage **4**

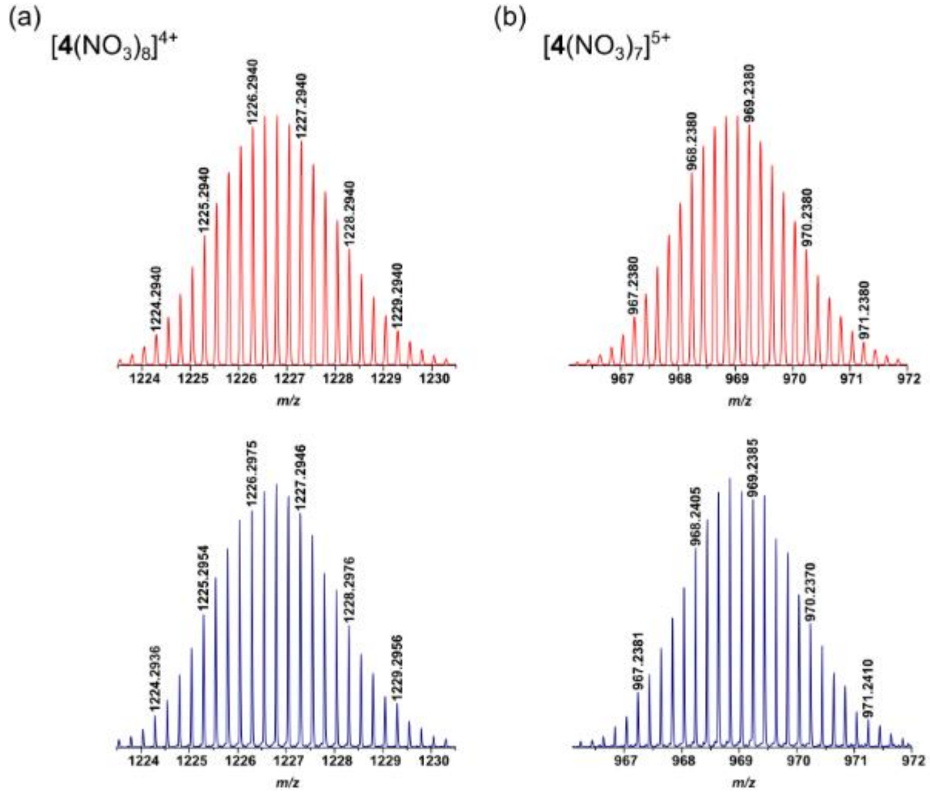

Figure S43: Isotopic distribution of selected peaks from cage **4** showing match between predicted (red) and experimentally (blue) observed patterns
(a) [**4**$(NO_3)_8$]$^{4+}$, (b) [**4**$(NO_3)_7$]$^{5+}$

S6 Stability studies of cage systems

S6.1 Cage 1

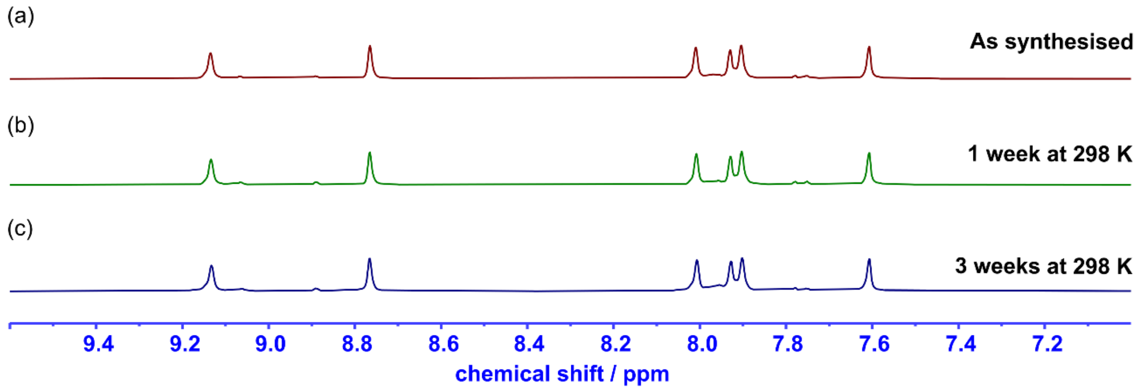

Figure S44: ^{1}H NMR spectra of cage **1**: (a) immediately after synthesis (maroon); (b) after leaving at 298 K for 1 week (green); and (c) after leaving at 298 K for 3 weeks (navy). (500 MHz, 298 K, DMSO-d_6)

S6.2 Cage 2

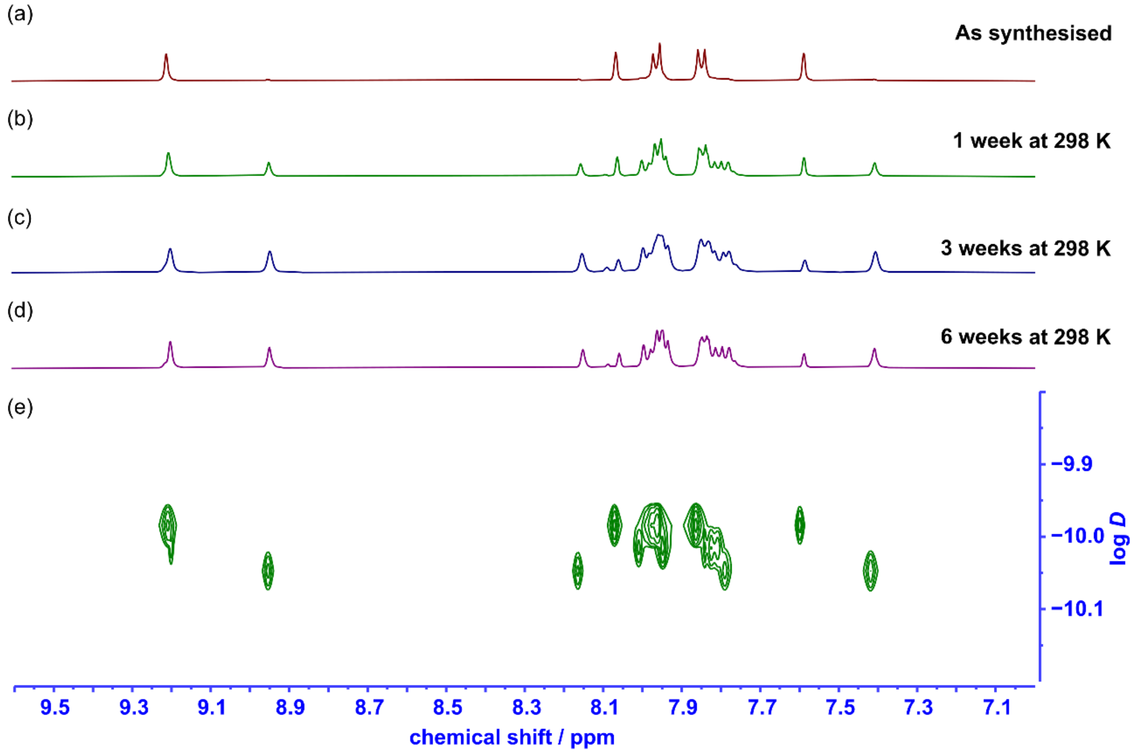

Figure S45: ^{1}H NMR spectra of cage **2**: (a) immediately after synthesis (maroon); (b) after leaving at 298 K for 1 week (green); (c) after leaving at 298 K for 3 weeks (navy); (d) after leaving at 298 K for 6 weeks (purple); and (e) DOSY trace after leaving at 298 K for 1 week. (500 MHz, 298 K, DMSO-d_6)

S6.2.1 Variable temperature NMR of cage 2

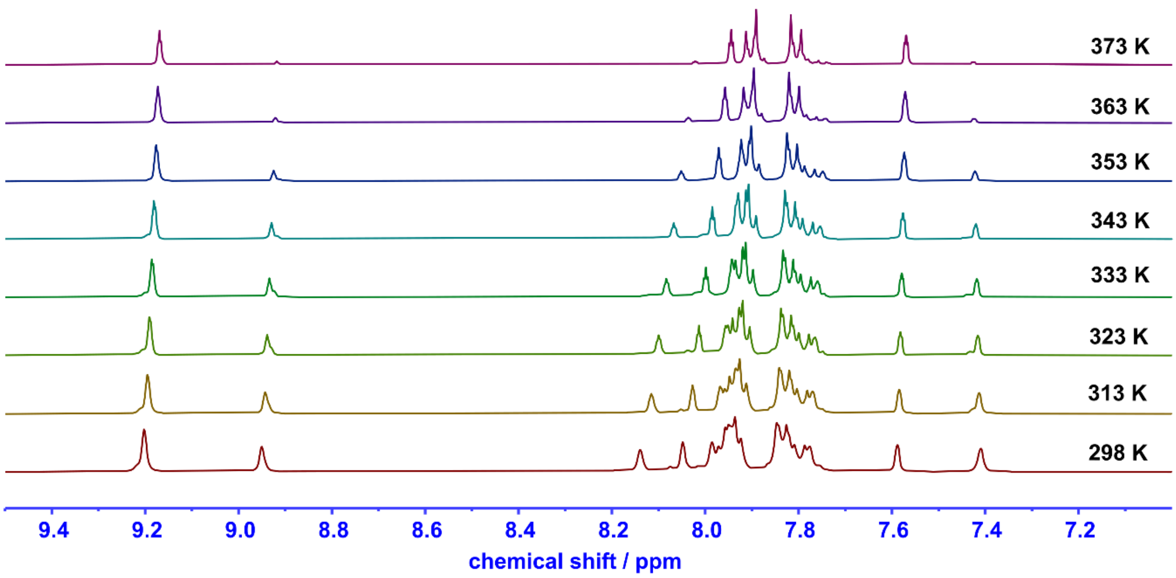

Figure S46: Variable temperature ^{1}H NMR spectra of a sample of cage **2**, which settled to an equilibrium after 3 weeks. (400 MHz, 298 K, DMSO-d_6)

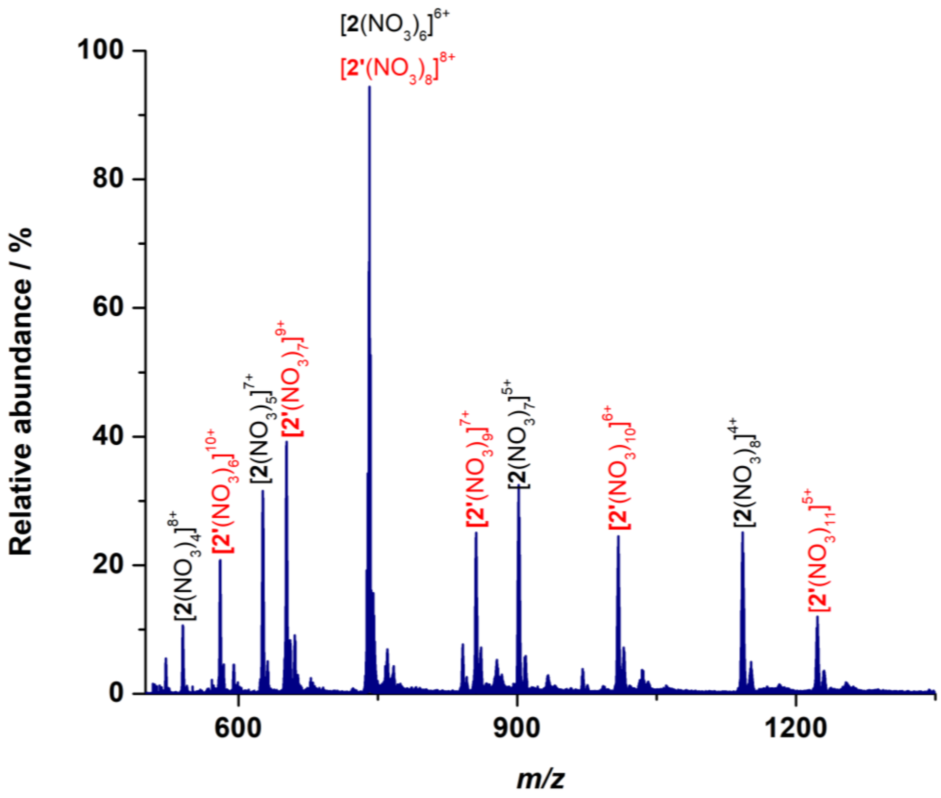

Figure S47: ESI-MS of cage **2** after leaving at 298 K for 6 weeks

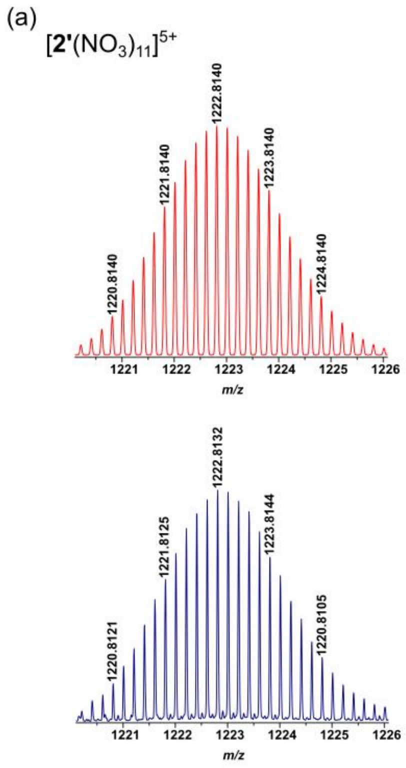

Figure S48: Isotopic distribution of selected peaks from cage **2’** showing match between predicted (red) and experimentally observed (blue) patterns
(a) [**2’**$(NO_3)_{11}$]$^{5+}$, (b) [**2’**$(NO_3)_{10}$]$^{6+}$.

S6.3 Cage 3

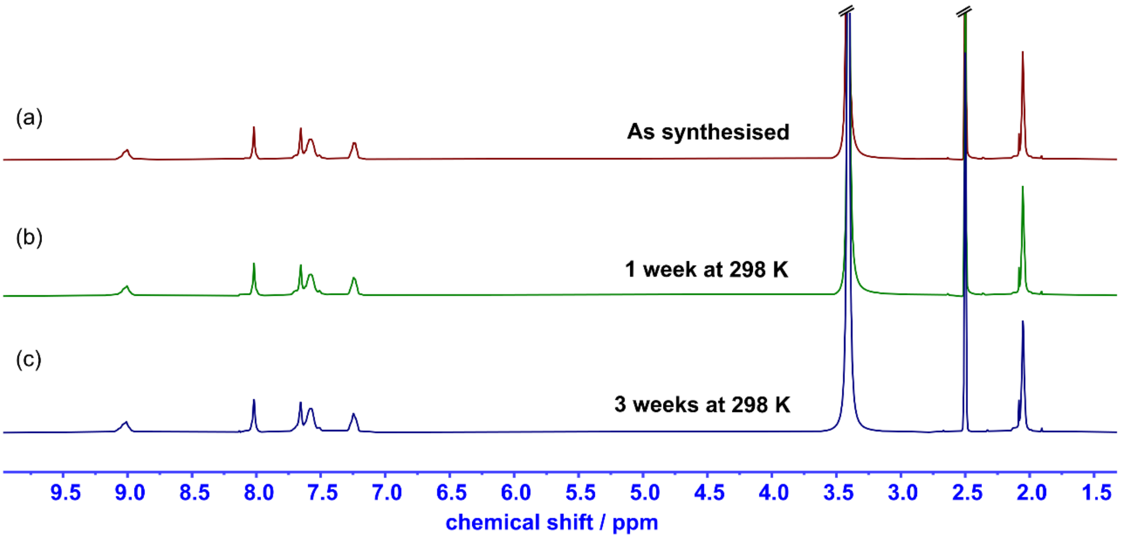

Figure S49: ^{1}H NMR spectra of cage **3**: (a) immediately after synthesis (maroon); (b) after leaving at 298 K for 1 week (green); and (c) after leaving at 298 K for 3 weeks (navy). (500 MHz, 298 K, DMSO-d_6)

S7 Determination of ligand vector angles and dihedral angles

The angles quoted in this work were determined from the single crystal X-ray diffraction data obtained. In the processing of the X-ray data, atom labels were assigned to each atom in the final Crystallographic Information File (.cif). These files were then opened within PyMOL. A single atom at a time was selected in the PyMOL interface and the following command was used to retrieve the (x,y,z) coordinates of that atom.

xyz = cmd.get_coords('sele', 1),

print xyz

The atom coordinates of all atoms in question were tabulated in Excel. 3D Cartesian vectors were defined from the position of one atom to another atom. For the vectors for calculation of the metal-ligand coordination vector angle, θ, the start of each vector was on the imidazole nitrogen atom and the end of each vector was on the palladium atom. The resultant 3D Cartesian vectors **A**(x,y,z) and **B**(x,y,z) were calculated through subtraction of the coordinates of the starting atom from the coordinates of the final atom.

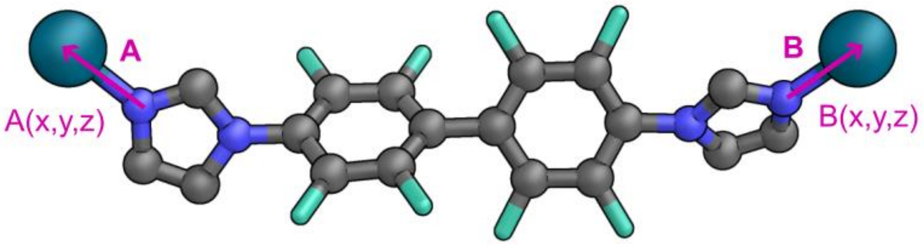

Figure S50: Vectors for calculation of the metal-ligand coordination vector angle, θ

As the two vectors **A**(x,y,z) and **B**(x,y,z) are not coplanar, the following mathematical formula was used to calculate the angle between the two 3D Cartesian vectors, with the vector components denoted Ax, Ay, Az, Bx, By, and Bz respectively.

$$\theta = \cos^{-1}\frac{(AxBx + AyBy + AzBz)}{\sqrt{Ax^2 + Ay^2 + Az^2} * \sqrt{Bx^2 + By^2 + Bz^2}}$$

Calculation of the dihedral angle, φ, was performed in Olex2. As the rings were not completely planar within the crystal structure, dihedral angles were calculated using the angles between the C–C or N–C bonds. For each biaryl bond, angles could be defined between two sets of four atoms (**1A**, **2**, **3**, and **4A**) or (**1B**, **2**, **3**, and **4B**). The desired four atoms were selected from the .cif in Olex2 and the dihedral angle was obtained from a list on the screen by keeping

the cursor on top of the fourth atom selected. The two values were then averaged to give an overall dihedral angle for each biaryl bond.

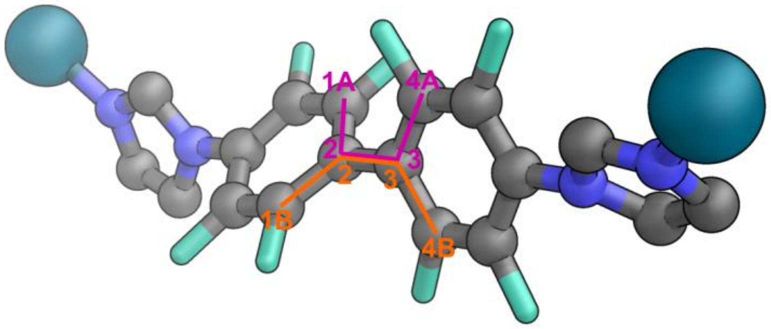

Figure S51: Vectors for calculation of the dihedral angle, φ

Within each structure, each crystallographically independent value of the angles θ and φ were calculated. For the octahedra, single mean angles θ_{av} and φ_{av} were then calculated for the overall structure, accounting for any statistical weighting due to ligands equivalent by symmetry in the crystal structure. For the square antiprism, mean angles θ_{av} and φ_{av} were then calculated for both square/triangle edges and triangle/triangle edges, with overall means also calculated. All mean values in the manuscript are quoted ± a standard deviation. The numbering of the atoms follows the same numbering convention as the numbers assigned in the Crystallographic Information File (.cif).

S7.1 Coordination vectors in $Pd_8L^1_{16}$ square antiprism 1

Table S1: Coordination vectors for square/triangle edges of cage 1

Atom/Vector	x	y	z	Atom/Vector	x	y	z
Pd5	5.478	8.522	76.894	Pd3	16.044	15.402	53.247
N11H	4.168	7.045	76.415	N11C	17.071	13.914	52.418
**A vector**	**1.31**	**1.48**	**0.48**	**A vector**	**−1.03**	**1.49**	**0.83**
Pd8	3.065	−2.833	64.902	Pd4	16.253	−1.582	53.383
N31H	1.753	−2.052	66.256	N31C	17.42	−0.118	52.693
**B vector**	**1.31**	**−0.78**	**−1.35**	**B vector**	**−1.17**	**−1.46**	**0.69**
**θ**			**91.15°**	**θ**			**95.90°**
Pd8	3.065	−2.833	64.902	Pd4	16.253	−1.582	53.383
N31G	1.845	−2.639	63.345	N11D	17.576	−1.863	54.868
**A vector**	**1.22**	**−0.19**	**1.56**	**A vector**	**−1.32**	**0.28**	**−1.49**
Pd7	1.675	6.794	50.924	Pd1	19.177	0.59	70.266
N11G	0.662	5.426	51.975	N31D	20.159	0.406	68.446
**B vector**	**1.01**	**1.37**	**−1.05**	**B vector**	**−0.98**	**0.18**	**1.82**
**θ**			**99.64°**	**θ**			**108.91°**
Pd7	1.675	6.794	50.924	Pd1	19.177	0.59	70.266
N11F	0.582	8.197	51.853	N11A	20.438	2.153	70.767
**A vector**	**1.09**	**−1.40**	**−0.93**	**A vector**	**−1.26**	**−1.56**	**−0.50**
Pd6	2.549	18.53	63.343	Pd2	18.507	17.413	70.119
N31F	1.21	17.834	61.978	N31A	19.858	15.941	70.512
**B vector**	**1.34**	**0.70**	**1.37**	**B vector**	**−1.35**	**1.47**	**−0.39**
**θ**			**101.03°**	**θ**			**95.45°**
Pd6	2.549	18.53	63.343	Pd2	18.507	17.413	70.119
N11E	1.627	17.598	64.847	N31B	19.386	17.823	68.359
**A vector**	**0.92**	**0.93**	**−1.50**	**A vector**	**−0.88**	**−0.41**	**1.76**
Pd5	5.478	8.522	76.894	Pd3	16.044	15.402	53.247
N31E	4.087	9.89	76.33	N11B	17.488	15.661	54.681
**B vector**	**1.39**	**−1.37**	**0.56**	**B vector**	**−1.44**	**−0.26**	**−1.43**
**θ**			**101.98°**	**θ**			**106.17°**

Table S2: Coordination vectors for triangle/triangle edges of cage 1

**Atom/Vector**	**x**	**y**	**z**	**Atom/Vector**	**x**	**y**	**z**
Pd3	16.044	15.402	53.247	Pd1	19.177	0.590	70.266
N31L	14.929	16.794	54.146	N31P	17.901	−0.914	69.853
**A vector**	**1.12**	**−1.39**	**−0.90**	**A vector**	**1.28**	**1.50**	**0.41**
Pd6	2.549	18.530	63.343	Pd8	3.065	−2.833	−64.900
N11L	3.541	19.458	61.758	N11P	4.355	−3.012	66.466
**B vector**	**−0.99**	**−0.93**	**1.59**	**B vector**	**−1.29**	**0.18**	**−1.57**
**θ**			**107.29°**	**θ**			**119.54°**
Pd6	2.549	18.530	63.343	Pd8	3.065	−2.833	64.902
N31K	3.962	19.274	64.673	N31O	4.475	−3.654	63.661
**A vector**	**−1.41**	**−0.74**	**−1.33**	**A vector**	**−1.41**	**0.82**	**1.24**
Pd2	18.507	17.413	70.119	Pd4	16.253	−1.582	53.383
N11K	17.068	18.745	69.639	N11O	15.020	−2.889	54.250
**B vector**	**1.44**	**−1.33**	**0.48**	**B vector**	**1.23**	**1.31**	**−0.87**
**θ**			**113.62°**	**θ**			**115.20°**
Pd2	18.507	17.413	70.119	Pd4	16.253	−1.582	53.383
N31J	17.629	16.923	71.890	N31N	14.938	−1.276	51.926
**A vector**	**0.88**	**0.49**	**−1.77**	**A vector**	**1.32**	**−0.31**	**1.46**
Pd5	5.478	8.522	76.894	Pd7	1.675	6.794	50.924
N11J	6.737	10.073	77.287	N11N	2.809	5.344	50.112
**B vector**	**−1.26**	**−1.55**	**−0.39**	**B vector**	**−1.13**	**1.45**	**0.81**
**θ**			**106.38°**	**θ**			**100.84°**
Pd5	5.478	8.522	76.894	Pd7	1.675	6.794	50.924
N31I	6.954	7.157	77.352	N11M	2.740	8.224	49.977
**A vector**	**−1.48**	**1.37**	**−0.46**	**A vector**	**−1.07**	**−1.43**	**0.95**
Pd1	19.177	0.590	70.266	Pd3	16.044	15.402	53.247
N11I	18.195	0.872	72.061	N31M	14.668	15.124	51.788
**B vector**	**0.98**	**−0.28**	**−1.80**	**B vector**	**1.38**	**0.28**	**1.46**
**θ**			**103.75°**	**θ**			**96.76°**

S7.2 Dihedral twist angles in $Pd_8L^1_{16}$ square antiprism 1

Table S3: Dihedral twist angles of perfluorophenyl-perfluorophenyl in square/triangle edges of cage 1

Atoms	φ	Atoms	φ
C25A-C24A-C44A-C43A	57.61°	C25E-C24E-C44E-C43E	60.89°
C23A-C24A-C44A-C45A	62.94°	C23E-C24E-C44E-C45E	66.43°
	**60.28°**		**63.66°**
C25B-C24B-C44B-C43B	62.31°	C25F-C24F-C44F-C43F	58.39°
C23B-C24B-C44B-C45B	52.25°	C23F-C24F-C44F-C45F	61.51°
	**57.28°**		**59.95°**
C25C-C24C-C44C-C45C	55.28°	C25G-C24G-C44G-C45G	56.98°
C23C-C24C-C44C-C43C	45.54°	C23G-C24G-C44G-C43G	56.49°
	**50.41°**		**56.74°**
C25D-C24D-C44D-C45D	52.48°	C25H-C24H-C44H-C45H	51.65°
C23D-C24D-C44D-C43D	55.21°	C23H-C24H-C44H-C43H	47.77°
	**53.85°**		**49.71°**

Table S4: Dihedral twist angles of perfluorophenyl-perfluorophenyl in triangle/triangle edges of cage 1

Atoms	φ	Atoms	φ
C25I-C24I-C44I-C43I	63.45°	C25M-C24M-C44M-C43M	54.65°
C23I-C24I-C44I-C45I	55.09°	C23M-C24M-C44M-C45M	67.24°
	**59.27°**		**60.95°**
C25J-C24J-C44J-C45J	47.83°	C25N-C24N-C44N-C45N	57.10°
C23J-C24J-C44J-C43J	58.61°	C23N-C24N-C44N-C43N	54.23°
	**53.22°**		**55.67°**
C25K-C24K-C44K-C43K	56.22°	C25O-C24O-C44O-C45O	49.14°
C23K-C24K-C44K-C45K	54.21°	C23O-C24O-C44O-C43O	58.27°
	**55.21°**		**53.71°**
C25L-C24L-C44L-C45L	50.99°	C25P-C24P-C44P-C43P	66.95°
C23L-C24L-C44L-C43L	57.59°	C23P-C24P-C44P-C45P	61.77°
	**54.29°**		**64.36°**

Table S5: Dihedral twist angles of perfluorophenyl-imidazole in square/triangle edges of cage 1

Atoms	φ	Atoms	φ
C14A-N13A-C21A-C26A	80.51°	C14E-N13E-C21E-C22E	41.26°
C12A-N13A-C21A-C22A	73.73°	C12E-N13E-C21E-C26E	54.92°
	**77.12°**		**48.09°**
C34A-N33A-C41A-C46A	56.22°	C34E-N33E-C41E-C46E	47.87°
C32A-N33A-C41A-C42A	37.69°	C32E-N33E-C41E-C42E	45.34°
	**46.96°**		**46.60°**
C14B-N13B-C21B-C26B	88.56°	C14F-N13F-C21F-C22F	44.27°
C12B-N13B-C21B-C22B	94.04°	C12F-N13F-C21F-C26F	47.26°
	**91.30°**		**45.76°**
C34B-N33B-C41B-C46B	56.62°	C34F-N33F-C41F-C46F	38.64°
C32B-N33B-C41B-C42B	65.44°	C32F-N33F-C41F-C42F	41.95°
	**61.03°**		**40.29°**
C14C-N13C-C21C-C26C	55.11°	C14G-N13G-C21G-C22G	49.54°
C12C-N13C-C21C-C22C	33.89°	C12G-N13G-C21G-C26G	45.62°
	**44.50°**		**47.58°**
C34C-N33C-C41C-C46C	56.12°	C34G-N33G-C41G-C46G	50.19°
C32C-N33C-C41C-C42C	61.55°	C32G-N33G-C41G-C42G	58.53°
	**58.84°**		**54.36°**
C14D-N13D-C21D-C26D	58.43°	C14H-N13H-C21H-C22H	41.37°
C12D-N13D-C21D-C22D	57.62°	C12H-N13H-C21H-C26H	48.76°
	**58.02°**		**45.07°**
C34D-N33D-C41D-C46D	43.17°	C34H-N33H-C41H-C42H	76.50°
C32D-N33D-C41D-C42D	50.64°	C32H-N33H-C41H-C46H	84.22°
	**46.91°**		**80.36°**

Table S6: Dihedral twist angle of perfluorophenyl-imidazole in triangle/triangle edges of cage 1

Atoms	φ	Atoms	φ
C14I-N13I-C21I-C22I	53.36°	C14M-N13M-C21M-C22M	48.06°
C12I-N13I-C21I-C26I	47.46°	C12M-N13M-C21M-C26M	41.70°
	**50.41°**		**44.88°**
C34I-N33I-C41I-C46I	43.23°	C34M-N33M-C41M-C46M	45.99°
C32I-N33I-C41I-C42I	51.10°	C32M-N33M-C41M-C42M	51.15°
	**47.17°**		**48.57°**
C14J-N13J-C21J-C26J	55.34°	C14N-N13N-C21N-C22N	44.24°
C12J-N13J-C21J-C22J	43.44°	C12N-N13N-C21N-C26N	44.07°
	**49.39°**		**44.16°**
C34J-N33J-C41J-C46J	48.12°	C34N-N33N-C41N-C42N	45.75°
C32J-N33J-C41J-C42J	46.27°	C32N-N33N-C41N-C46N	48.55°
	**47.19°**		**47.15°**
C14K-N13K-C21K-C22K	66.91°	C14O-N13O-C21O-C26O	63.29°
C12K-N13K-C21K-C26K	78.67°	C12O-N13O-C21O-C22O	62.88°
	**72.79°**		**63.08°**
C34K-N33K-C41K-C46K	47.14°	C34O-N33O-C41O-C42O	43.80°
C32K-N33K-C41K-C42K	48.34°	C32O-N33O-C41O-C46O	47.33°
	**47.74°**		**45.57°**
C14L-N13L-C21L-C26L	58.01°	C14P-N13P-C21P-C26P	43.83°
C12L-N13L-C21L-C22L	55.37°	C12P-N13P-C21P-C22P	46.60°
	**56.69°**		**45.21°**
C34L-N33L-C41L-C46L	44.80°	C34P-N33P-C41P-C42P	48.95°
C32L-N33L-C41L-C42L	53.47°	C32P-N33P-C41P-C46P	51.57°
	**49.14°**		**50.26°**

S7.3 Coordination vectors in $Pd_6L^2_{12}$ octahedron 2

Due to crystallographic symmetry, only six of the 12 edges in octahedron **2** are unique.

Table S7: Coordination vectors of cage 2

Atom/Vector	x	y	z
Pd1	−2.40	−1.64	10.02
N11A	−2.92	−0.95	11.80
**A vector**	**0.53**	**−0.69**	**−1.79**
Pd2	2.27	10.49	21.08
N31A	0.93	8.99	20.95
**B vector**	**1.34**	**1.50**	**0.13**
**θ**			**97.87°**
Pd1	−2.40	−1.64	10.02
N31B	−1.04	−2.75	10.95
**A vector**	**−1.36**	**1.12**	**−0.93**
Pd3	13.68	−0.24	15.02
N11B	12.35	−1.69	14.89
**B vector**	**1.34**	**1.45**	**0.12**
**θ**			**94.54°**
Pd1	−2.40	−1.64	10.02
N31C	−1.83	−2.24	8.21
**A vector**	**−0.57**	**0.60**	**1.81**
Pd2	9.02	3.86	−0.73
N11C	7.69	2.36	−0.76
**B vector**	**1.34**	**1.49**	**0.03**
**θ**			**87.26°**
Pd1	−2.40	−1.64	10.02
N31D	−3.69	−0.40	9.15
**A vector**	**1.29**	**−1.24**	**0.87**
Pd3	−2.39	14.59	5.33
N11D	−3.61	13.00	5.15
**B vector**	**1.22**	**1.59**	**0.19**
**θ**			**93.38°**
Pd2	2.27	10.49	21.08
N11E	0.92	11.71	20.25
**A vector**	**1.36**	**−1.22**	**0.82**
Pd3	−2.39	14.59	5.33
N31E	−2.98	14.89	7.24
**B vector**	**0.59**	**−0.30**	**−1.90**
**θ**			**95.74°**
Pd3	−2.39	14.59	5.33
N31F	−1.73	14.21	3.50
**A vector**	**−0.66**	**0.38**	**1.83**
Pd2	9.02	3.86	−0.73
N11F	7.65	5.12	−1.44
**B vector**	**1.37**	**−1.27**	**0.71**
**θ**			**91.08°**

S7.4 Dihedral twist angles in $Pd_6L^2_{12}$ octahedron 2

Due to crystallographic symmetry, only six of the 12 edges in octahedron **2** are unique.

Table S8: Dihedral twist angles of phenyl-phenyl in cage 2

Atoms	φ
C25A-C24A-C44A-C45A	34.65°
C23A-C24A-C44A-C43A	37.45°
	**36.05°**
C25B-C24B-C44B-C45Y	31.20°
C23B-C24B-C44B-C43Y	30.13°
	**30.67°**
C25W-C24C-C44C-C45C	33.04°
C23W-C24C-C44C-C43C	32.43°
	**32.73°**
C25D-C24D-C44D-C45D	25.95°
C23D-C24D-C44D-C43D	26.53°
	**26.24°**
C25E-C24E-C44E-C45E	25.10°
C23E-C24E-C44E-C43E	30.17°
	**27.63°**
C25F-C24F-C44F-C45F	36.74°
C23F-C24F-C44F-C43F	34.63°
	**35.68°**

Table S9: Dihedral twist angles of phenyl-imidazole in cage 2

Atoms	φ	Atoms	φ
C14A-N13A-C21A-C26A	26.36°	C14D-N13D-C21D-C26D	32.18°
C12A-N13A-C21A-C22A	32.66°	C12D-N13D-C21D-C22D	21.89°
	**29.51°**		**27.04°**
C34A-N33A-C41A-C46A	28.77°	C34D-N33D-C41D-C46D	33.84°
C32A-N33A-C41A-C42A	22.85°	C32D-N33D-C41D-C42D	25.54°
	**25.81°**		**29.69°**
C14B-N13B-C21B-C26B	17.65°	C14E-N13E-C21E-C26E	22.36°
C12B-N13B-C21B-C22B	27.73°	C12E-N13E-C21E-C22E	18.56°
	**22.69°**		**20.46°**
C34B-N33B-C41B-C46B	37.86°	C34E-N33E-C41E-C46E	34.05°
C32B-N33B-C41B-C42B	27.62°	C32E-N33E-C41E-C42E	40.05°
	**32.74°**		**37.05°**
C14C-N13C-C21C-C26C	31.12°	C14F-N13F-C21F-C26F	60.96°
C12C-N13C-C21C-C22C	52.33°	C12F-N13F-C21F-C22F	49.43°
	**41.73°**		**55.19°**
C34C-N33C-C41C-C46C	23.39°	C34F-N33F-C41F-C46F	16.54°
C32C-N33C-C41C-C42C	33.62°	C32F-N33F-C41F-C42F	18.82°
	**28.51°**		**17.68°**

S7.5 Coordination vectors in $Pd_6L^3_{12}$ octahedron 3

Due to crystallographic symmetry, the A and the B edges (marked with a *) occur in four places, whereas the C and D edges occur in two places. This weighting is accounted for in the mean calculations.

Table S10: Coordination vectors of cage 3

Atom/Vector	x	y	z
Pd2	15.932	23.498	20.248
N11C	15.365	24.778	18.789
**A vector**	**0.57**	**−1.28**	**1.46**
Pd2	9.896	23.498	4.186
N11C	10.463	24.778	5.645
**B vector**	**−0.57**	**−1.28**	**−1.46**
**θ**			**101.45°**
Pd2	15.932	23.498	20.248
N31A	17.795	23.417	19.540
**A vector**	**−1.86**	**0.08**	**0.71**
Pd1	24.014	15.321	8.051
N11A	24.489	16.720	9.401
**B vector**	**−0.48**	**−1.40**	**−1.35**
**θ**			**92.64°***
Pd1	24.014	15.321	8.051
N11B	23.459	16.713	6.721
**A vector**	**0.56**	**−1.39**	**1.33**
Pd2	9.896	23.498	4.186
N31B	11.785	23.532	3.500
**B vector**	**−1.89**	**−0.03**	**0.69**
**θ**			**91.26°***
Pd2	15.932	23.498	20.248
N11D	16.442	22.174	21.664
**A vector**	**−0.51**	**1.32**	**−1.42**
Pd2	15.932	7.143	20.248
N11D	16.442	8.467	21.664
**B vector**	**−0.51**	**−1.32**	**−1.42**
**θ**			**82.68°**

S7.6 Dihedral twist angles in $Pd_6L^3_{12}$ octahedron 3

Due to crystallographic symmetry, imidazole-phenyl twist angles at the end of the A and the B edges are different, whereas imidazole-phenyl twist angles at the end of the C and D edges are equivalent by symmetry.

Table S11: Dihedral twist angles of phenyl-phenyl in cage 3

Atoms	φ
C25A-C24A-C44A-C45A	62.68°
C23A-C24A-C44A-C43A	69.26°
	**65.97°**
C25B-C24B-C44B-C45B	76.23°
C23B-C24B-C44B-C43B	77.02°
	**76.63°**
C25C-C24C-C24C2-C25C2	78.54°
C23C-C24C-C24C2-C23C2	78.61°
	**78.58°**
C25D-C24D-C24E-C25E	67.33°
C23D-C24D-C24E-C23E	69.55°
	**68.44°**

Table S12: Dihedral twist angles of phenyl-imidazole in cage 3

Phenyl-imidazole	
**Atoms**	**φ**
C14A-N13A-C21A-C22A	35.99°
C12A-N13A-C21A-C26A	33.14°
	**34.56°**
C34A-N33A-C41A-C42A	38.37°
C32A-N33A-C41A-C46A	39.29°
	**38.83°**
C14B-N13B-C21B-C26B	35.61°
C12B-N13B-C21B-C22B	32.85°
	**34.23°**
C34B-N33B-C41B-C46B	26.53°
C32B-N33B-C41B-C42B	24.64°
	**25.58°**
C14C-N13C-C21C-C22C	28.28°
C12C-N13C-C21C-C26C	31.03°
	**29.65°**
C14D-N13D-C21D-C22D	38.59°
C12D-N13D-C21D-C26D	26.52°
	**32.55°**

S7.7 Dihedral twist angles in ligand L^1

Table S13: Dihedral twist angles in ligand L^1

Perfluorophenyl-perfluorophenyl	
**Atoms**	**φ**
C8-C9-C20-C21	51.59°
C10-C9-C20-C19	49.71°
	**50.65°**
**Perfluorophenyl-imidazole**	
C3-N4-C6-C7	40.06°
C5-N4-C6-C11	40.03°
	**40.05°**
C14-N16-C17-C22	37.10°
C16-N16-C17-C18	36.93°
	**37.01°**

S7.8 Dihedral twist angles in ligand L^2

Table S14: Dihedral twist angles in ligand L^2

Phenyl-phenyl	
**Atoms**	**φ**
C8-C9-C20-C21	19.69°
C10-C9-C20-C19	21.71°
	**20.70°**
**Phenyl-imidazole**	
C3-N4-C6-C7	7.48°
C5-N4-C6-C11	9.99°
	**8.73°**
C14-N16-C17-C22	16.86°
C16-N16-C17-C18	15.46°
	**16.16°**

S7.9 Dihedral twist angles in ligand L^3

Table S15: Dihedral twist angles in ligand L^3

Phenyl-phenyl	
**Atoms**	**φ**
C9-C10-C22-C21	80.30°
C11-C10-C22-C23	83.09°
	**81.69°**
**Phenyl-imidazole**	
C4-N3-C7-C8	32.90°
C2-N3-C7-C12	36.15°
	**34.52°**
C17-N16-C19-C20	35.18°
C15-N16-C19-C24	38.43°
	**36.81°**

S7.10 Dihedral twist angles in ligand L^4

Table S16: Dihedral twist angles in ligand L^4

Phenyl-phenyl	
**Atoms**	**φ**
C23-C24-C24-C23	49.96°
C25-C24-C24-C25	49.41°
	**49.69°**
**Phenyl-imidazole**	
C15-N14-C21-C26	57.53°
C13-N14-C21-C22	61.10°
	**59.32°**

S8 Cavity modelling

Single crystal structures of the cages were used to calculate the solvent accessible void and electrostatic potential (ESP) surfaces of the cage cavities using CageCavityCalc (*C*3) tool developed by the Duarte group.[4]

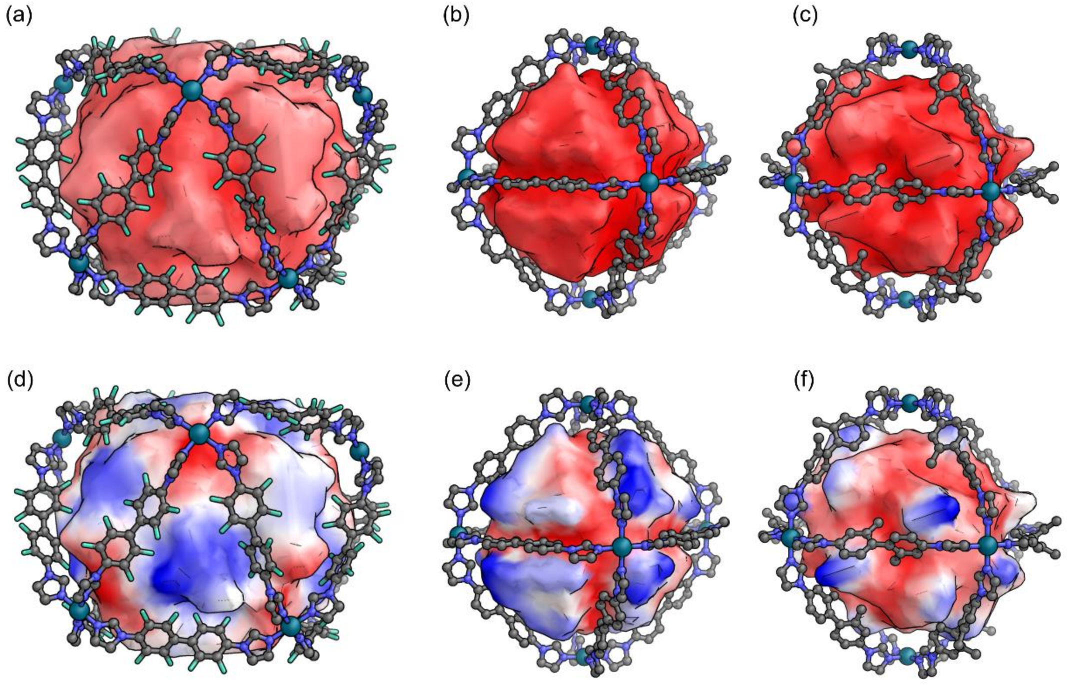

Figure S52: Computed cavity volumes for the cages: (a) cage **1**; (b) cage **2**; (c) cage **3**. Electrostatic potential (ESP) mapped on the cage cavities: (d) cage **1**; (e) cage **2**; (f) cage **3**. For the ESP figures red = positive and blue = negative.

Table S17: Computed cavity diameters and volumes of cages

Cage	Diameter / Å	Volume / Å^3
**1**	26.8	5420
**2**	23.9	3005
**3**	23.7	2813

S9 X-ray crystallography

Single crystals were selected and mounted using Fomblin® (YR-1800 perfluoropolyether oil) on a polymer-tipped MiTeGen MicroMountTM. Crystals **2**, **3**, **L^1**, **L^2**, **L^3** and **L^4** were cooled rapidly to 120 K in a stream of cold N_2 using an Oxford Cryosystems open flow cryostat.[5] Crystal **1** was rapidly cooled in liquid nitrogen for shipping to Diamond Light Source and was cooled to 100 K in a stream of cold N_2 during data acquisition using an Oxford Cryosystems open flow cryostat. Single crystal X-ray diffraction data for **2**, **3**, and **L^2** were collected on an XtaLAB PRO MM007 (PILATUS3 R 200K Hybrid Pixel Array detector, mirror-monochromated Cu-K_α radiation source; λ = 1.54184 Å, ω scans). Single crystal X-ray diffraction data for **L^1**, **L^3** and **L^4** were collected on an Oxford Diffraction GV1000 (TitanS2 CCD area detector, mirror-monochromated Cu-Kα radiation source; λ = 1.54184 Å, ω scans). Cell parameters were refined from the observed positions of all strong reflections and absorption corrections were applied using a Gaussian numerical method with beam profile correction (CrysAlisPro).[6] X-ray diffraction data for **1** was collected in Experiment Hutch 1 (EH1) of Beamline I19, at Diamond Light Source.[7] The data were collected at a wavelength of 0.6889 Å on a Fluid Film Devices 3-circle fixed-chi diffractometer using a Dectris Pilatus 2M detector. The collected frames were integrated using XIA26 software.[8]

Structures were solved within Olex2[9] by dual space iterative methods (SHELXT)[10] and all non-hydrogen atoms refined by full-matrix least-squares on all unique F^2 values with anisotropic displacement parameters (SHELXL).[11] Hydrogen atoms were refined with constrained riding geometries and thermal parameters linked to U_{iso} of their parent atoms. Hydrogen atoms were refined both freely (see specific crystal structure and refinement details section for each crystal) and with constrained riding geometries and thermal parameters linked to U_{iso} of their parent atoms. Structures were checked with checkCIF.[12] CCDC 2450736 (cage **1**); CCDC 2450737 (cage **2**); CCDC 2450738 (cage **3**); CCDC 2450739 (ligand **L^1**); CCDC 2450740 (ligand **L^2**); 2450741 (ligand **L^3**) and 2476005 (ligand **L^4**) contain the supplementary data for these compounds. These data can be obtained free of charge from The Cambridge Crystallographic Data Centre via www.ccdc.cam.ac.uk/data_request/cif.

Table 18. Experimental details for cages 1, 2, and 3; ligands L¹, L², L³ and L⁴

	1	2	3	L¹	L²	L³	L⁴
Chemical formula	$C_{288}H_{96}F_{128}N_{64}Pd_8 \cdot NO_3 \cdot 15[NO_3] \cdot 80[C_2H_6SO] \cdot 40[H_2O]$	$C_{216}H_{168}N_{48}Pd_6 \cdot 8(NO_3) \cdot 2(C_2H_6OS) \cdot 4[NO_3] \cdot 40[C_2H_6SO] \cdot 160[H_2O]$	$C_{240}H_{216}N_{48}Pd_6 \cdot 5.55(NO_3) \cdot 6[NO_3] \cdot 280[H_2O]$	$C_{18}H_6F_8N_4$	$C_{18}H_{14}N_4$	$C_{20}H_{18}N_4$	$C_{20}H_{18}N_4$
M_r	15784.21	11325.26	10771.55	430.27	286.33	314.38	314.38
Crystal system, space group	Monoclinic, $P2_1/n$	Triclinic, $P\overline{1}$	Monoclinic, $C2/m$	Triclinic, $P\overline{1}$	Triclinic, $P\overline{1}$	Triclinic, $P\overline{1}$	Monoclinic, $C2/c$
Temperature / K	100	120	120	120	120	120	120
a, *b*, *c* / Å	23.9292 (5), 27.4328 (6), 101.0207 (17)	22.6979 (3), 22.9505 (3), 24.0237 (4)	31.9252 (3), 30.6414 (3), 25.1833 (2)	7.4513 (7), 9.9556 (9), 12.1471 (9)	10.4775 (3), 11.08226 (16), 14.0465 (2)	5.7222 (5), 8.0511 (5), 17.5832 (12)	13.7948 (3), 9.16890 (10), 13.6444 (3)
α, β, γ / °	90, 95.084 (2), 90	108.716 (2), 113.318 (2), 94.731 (1)	90, 104.011 (1), 90	104.406 (7), 104.745 (7), 108.395 (8)	72.7379 (13), 68.3735 (18), 62.4714 (19)	96.433 (6), 93.583 (6), 95.924 (6)	90, 113.043 (2), 90
V / Å³	66054 (2)	10564.3 (3)	23902.2 (4)	771.89 (13)	1328.63 (5)	798.41 (10)	1588.09(6)
Z	4	1	2	2	4	2	4
Radiation type	Synchrotron, 0.6889 Å	Cu K_a	Cu K_a	Cu K_a	Cu K_a	Cu K_a	Cu K_a
μ / mm⁻¹	0.54	4.58	2.15	1.63	0.70	0.63	0.63
Crystal size / mm	0.1 × 0.08 × 0.04	0.25 × 0.16 × 0.10	0.17 × 0.15 × 0.07	0.25 × 0.11 × 0.03	0.19 × 0.08 × 0.03	0.07 × 0.05 × 0.02	0.22 × 0.16 × 0.09
Diffractometer	Diamond Light Source beamline i19-EH1	XtalLAB PRO *MM007*, PILATUS3 R 200K	XtalLAB PRO *MM007*, PILATUS3 R 200K	SuperNova, Titan S2	XtalLAB PRO *MM007*, PILATUS3 R 200K	SuperNova, Titan S2	SuperNova, Titan S2
T_{min}, T_{max}	0.837, 1.0	0.470, 1.000	0.623, 1.000	0.798, 1.000	0.718, 1.000	0.946, 1.000	0.606,1.000
No. of measured, independent and observed [I > 2σ(I)] reflections	167362, 31333, 20734	139609, 30339, 22417	260434, 25039, 21203	5028, 2916, 2190	23421, 5390, 4841	7811, 2993, 2532	10406, 1558, 1506
R_{int}	0.086	0.102	0.057	0.040	0.023	0.037	0.016
(sin θ/λ)$_{max}$ / Å⁻¹	0.385	0.556	0.628	0.616	0.629	0.617	0.617
$R[F^2 > 2s(F^2)]$, $wR(F^2)$, S	0.118, 0.367, 1.45	0.103, 0.319, 1.26	0.068, 0.236, 1.08	0.047, 0.127, 1.01	0.038, 0.117, 1.04	0.052, 0.143, 1.05	0.037, 0.095, 1.07
No. of reflections	31333	30339	25039	2916	5390	2993	1558
No. of parameters	4429	1542	981	297	581	219	110
No. of restraints	27522	5459	3751	428	2072	0	0
$\Delta\rho_{max}$, $\Delta\rho_{min}$ / e Å⁻³	1.41, −0.80	2.15, −0.83	1.31, −0.77	0.31, −0.32	0.28, −0.24	0.24, −0.26	0.18, −0.33
CCDC	2450736	2450737	2450738	2450739	2450740	2450741	2476005

S9.1 Single crystal structure of $Pd_8L^1_{16}$ square antiprism 1

Single crystals of cage **1** were grown via slow vapour diffusion of EtOAc into DMSO. A suitable crystal was selected and mounted using Fomblin (YR-1800 perfluoropolyether oil) on a Fluid Film Devices diffractometer. The crystal was kept at 100(2) K during data collection.

S9.1.1 Specific crystal structure and refinement details for 1

The crystals of the porous metallosupramolecular cage complex diffracted weakly with a low-resolution diffraction limit of 1.30 Å despite the use of a synchrotron radiation source. A large number of reflections (62) were omitted from the refinement as they either had error/esd or significant disagreement between Fobs and Fcalc. Because of the low-resolution diffraction limit the refinement has a low data to parameter ratio of 7.1. Extensive use was made of restraints to aid refinement of the structure given the low data to parameter ratio. Rigid bond and similarity restraints were applied to all anisotropic displacement parameters in the structure. Refinement of the structure with non-metal atoms modelled isotropically resulted in a larger R1 value thus supporting the decision to model the atoms anisotropically despite the low data to parameter ratio. All symmetry equivalent ligand moieties were restrained to have similar geometries (SAME, SADI). The anisotropic displacement parameter of carbon atom C32J was restrained to have more isotropic character (ISOR).

A large void region in the unit cell (both inside and outside the cage complex) contained diffuse counterion and solvent residues which could not be identified or modelled from the electron density map. The Olex2 solvent mask routine was used to account for the scattering contribution from this region. The contents of the asymmetric void were assigned as a mixture of counterions, DMSO solvent, and water solvent residues chosen to satisfy charge balance, the volume of the void, and to a lesser extent the calculated electron content.

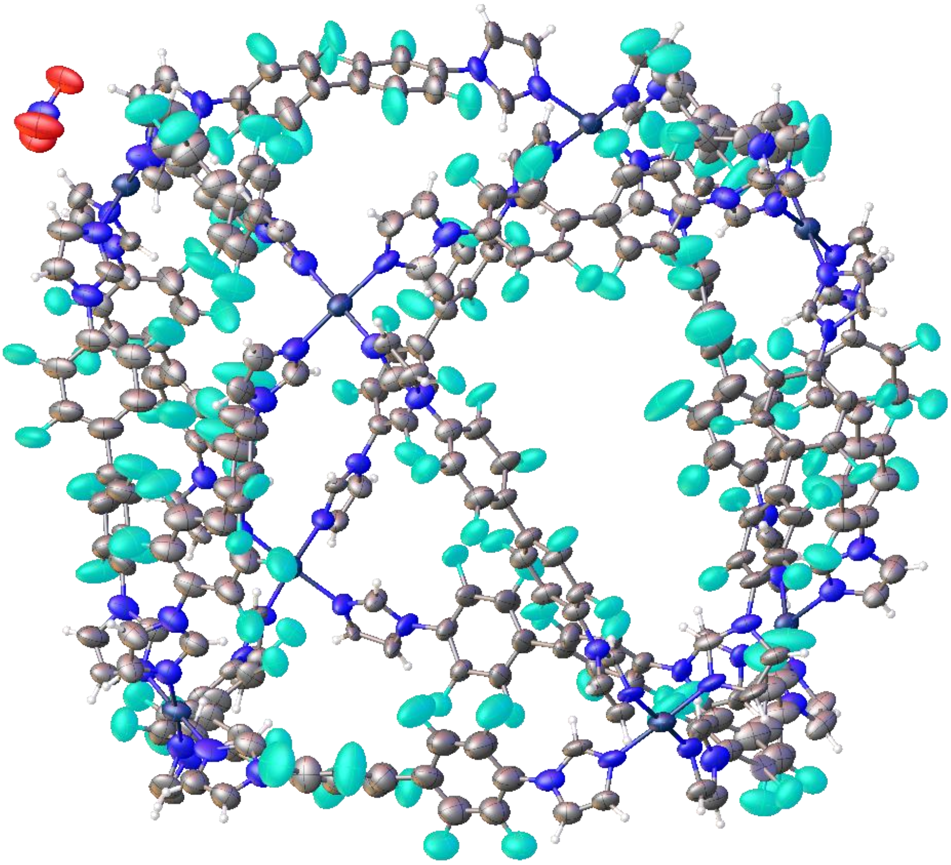

Figure S53: Image of **1** at 50% ellipsoids

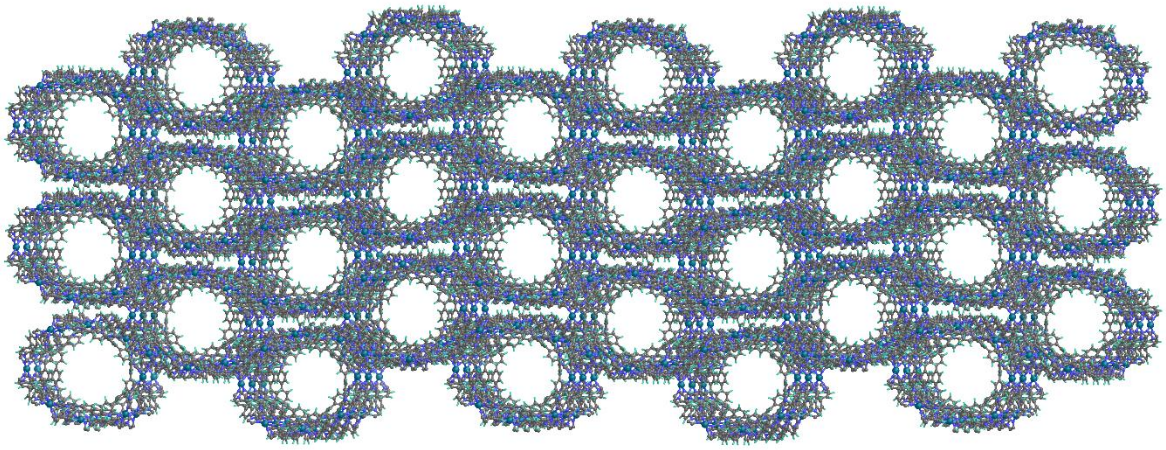

Figure S54: Packing structure of **1** showing the channels

S9.2 Single crystal structure of cage 2

Single crystals of cage **2** were grown via slow vapour diffusion of EtOAc into DMSO. A suitable crystal was selected and mounted using Fomblin (YR-1800 perfluoropolyether oil) on a XtalLAB PRO MM007, PILATUS3 R 200K diffractometer. The crystal was kept at 120(2) K during data collection.

S9.2.1 Specific crystal structure and refinement details for 2

The crystals of the large metallosupramolecular cage complex diffracted weakly with a low-resolution diffraction limit of 0.90 Å, likely as a consequence of large regions of diffuse solvent residues and counterions. Data used in the refinement was truncated to a resolution of 0.90 Å.

Three ligand phenyl ring moieties in the structure are modelled with conformational disorder with the rings in two orientations, the occupancies of which were refined and each pair respectively constrained to sum to unity resulting in values of 0.56(1) and 0.69(2) for the major components (one pair of adjacent disordered rings share occupancy parameters). Each disordered ring has common shared atom positions with its counterparts at the 1 and 4-positions where they are connected to neighbouring ring moieties. The geometries of the disordered rings were restrained to have planar geometries (FLAT).

The geometries of all ligands, nitrate anions, and DMSO solvent residues were restrained to have similar geometries that reflected their ideal symmetry (SAME, SADI). Rigid bond and similarity restraints were applied to the anisotropic displacement parameters of all carbon, nitrogen, oxygen, and sulfur atoms in the structure (RIGU, SIMU).

The occupancies of the two DMSO solvent residues were refined before being fixed at values of 0.5 in each case.

The large void regions were treated with the Olex2 solvent mask routine as no sensible models for the remaining electron density could be developed. The assumed contents of the masked region included nitrate anions to balance the charge of the main cage residue and a mixture of DMSO and water solvent molecules sufficient to give a plausible atomic volume per atom in the void region.

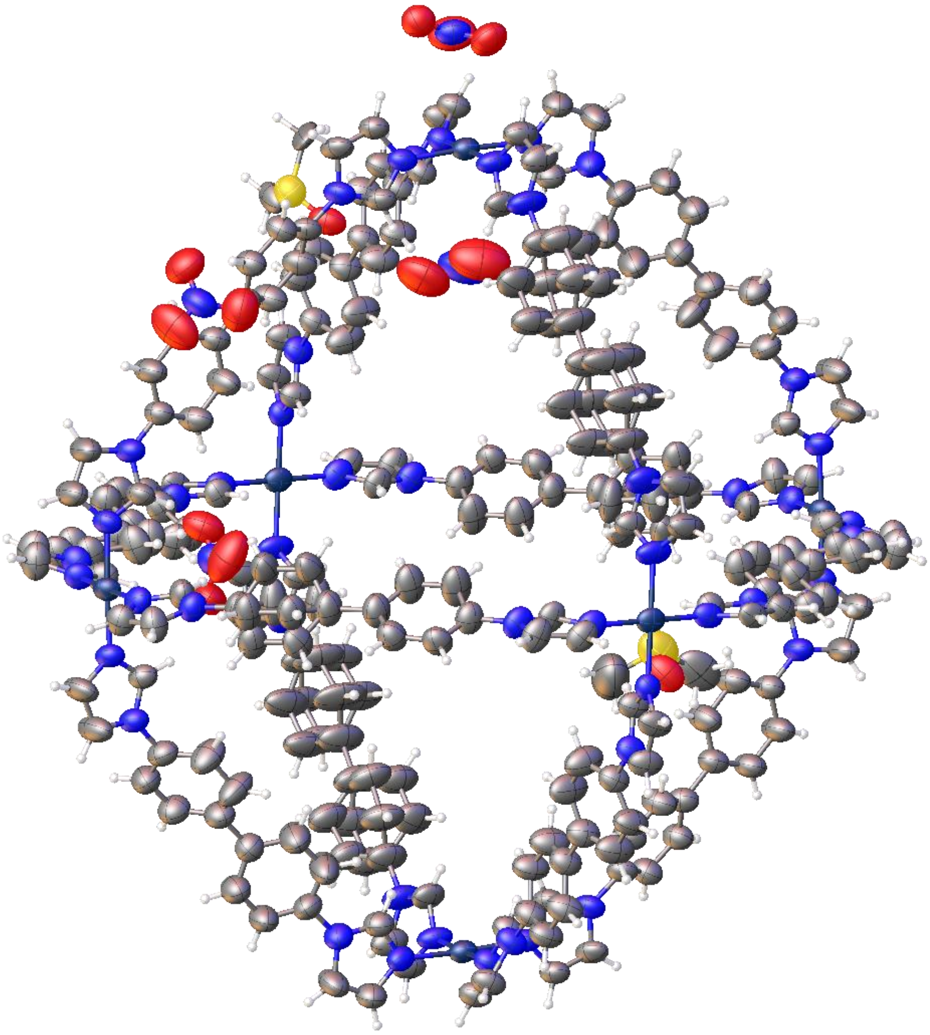

Figure S55: Image of **2** at 50% ellipsoids

S9.3 Single crystal structure of cage 3

Single crystals of cage **3** were grown via slow vapour diffusion of EtOAc into DMSO. A suitable crystal was selected and mounted using Fomblin (YR-1800 perfluoropolyether oil) on a XtalLAB PRO MM007, PILATUS3 R 200K diffractometer. The crystal was kept at 120(2) K during data collection.

S9.3.1 Specific crystal structure and refinement details for 3

The crystals of the large metallosupramolecular cage complex diffracted strongly and to high resolution despite the presence of large regions of diffuse counterions and solvent in the unit cell. To aid refinement of the large and disordered structure rigid bond and similarity restraints were applied to the thermal displacement parameters of all atoms in the structure (RIGU, SIMU). The thermal displacement parameters of the disordered methylphenyl moieties were additionally restrained to have more isotropic character (ISOR). The geometries of all symmetry equivalent ligand moieties in the structure, (i.e., the methylphenyl-imidazole fragments) were restrained to have similar 1,2 and 1,3 bonds distances (SAME, SADI). All hydrogen atoms were geometrically placed and refined with a riding model.

Three of the ligand methylphenyl residues are conformationally disordered with phenyl rings found in two orientations in each case. The occupancies of the respective disorder components were refined and constrained to sum to unity resulting in occupancies of 0.74(1), 0.64(1), and 0.51(1) for the major components. In the case of disorder pair C21D/E in which the ligand straddles a mirror plane, the components were placed in parts −1 and −2 to prevent them from bonding to their geometrically incompatible symmetry equivalent parts. The geometries of the disordered ligands were restrained to have co-planar geometries for the carbon atoms of the imidazole and phenyl rings that are connected to each other (FLAT). The geometries of the disordered methyl phenyl moieties were restrained to have planar geometry (FLAT).

A total of three nitrate counterions are expected in the asymmetric unit to charge balance the 1.5 Pd(II) cations. Two nitrate sites can be determined in the structure, both adjacent to Pd(II) metal centres. The occupancies of each nitrate anion were refined resulting in crystallographic occupancies of 0.97(1) and 0.42(1). The geometries of the nitrate anions were geometrically restrained to be similar, symmetrical, and planar (SADI, FLAT).

No further counterions or solvent residues could be identified and modelled in the large diffuse void volumes both inside and outside the cage molecule. A solvent mask was calculated and 4438 electrons were found in a volume of 14128 Å^3 in 1 void per unit cell. The void volume was assigned to contain 6[NO_3], 280[H_2O] per asymmetric unit which account for 11572

electrons per unit cell. The contents were chosen to charge balance the structure and fill the remaining void with an appropriate volume of water solvent molecules. Solvent contents were chosen to match the void volume rather than the electron estimation, as it is assumed that the latter is more prone to erroneous values. Water is a likely identity for diffuse solvent in a crystal grown from DMSO solution, however the presence of DMSO in the void region cannot be discounted (though it would likely be more apparent in the electron density map).

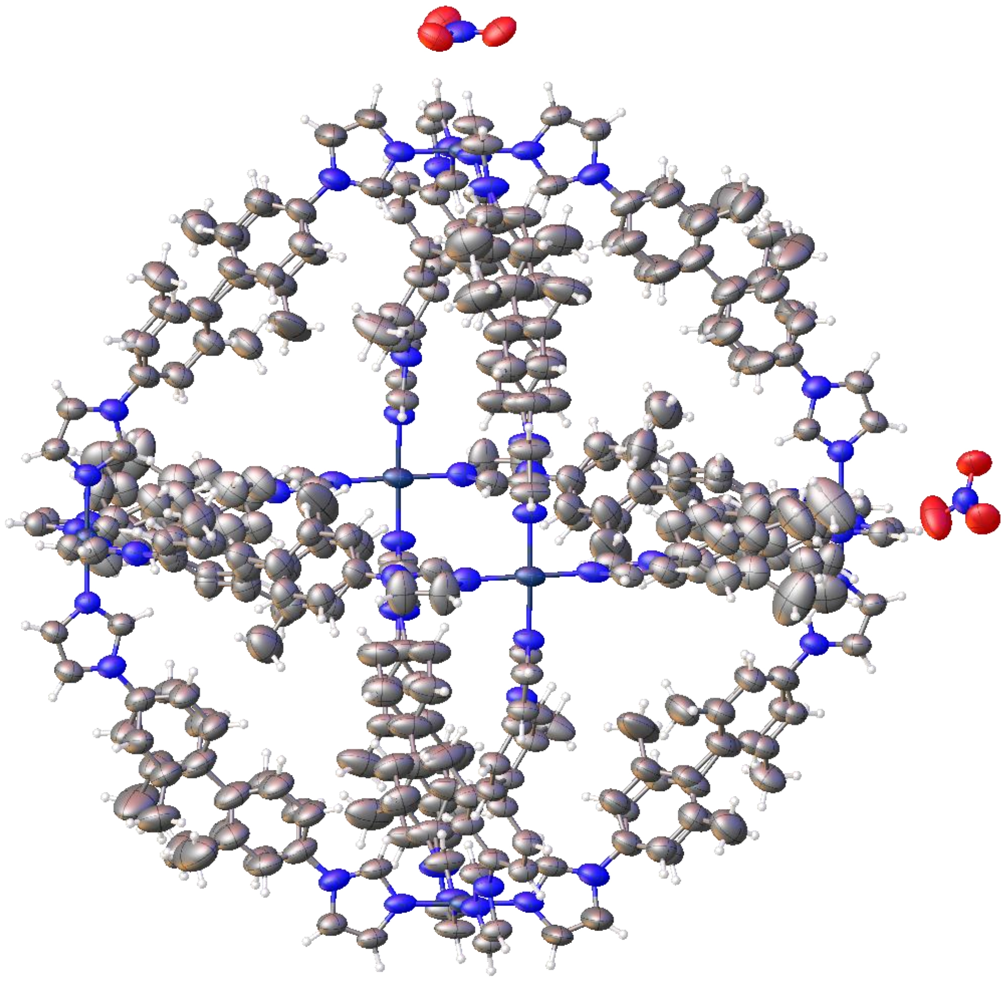

Figure S56: Image of **3** at 50% ellipsoids

S9.4 Single crystal structure of ligand L[1]

Single crystals of ligand **L[1]** were grown via vapour diffusion of hexane into chloroform. A suitable crystal was selected and mounted using Fomblin (YR-1800 perfluoropolyether oil) on an Oxford Diffraction GV1000 (TitanS2 CCD area detector, mirror-monochromated Cu-Kα radiation source; λ = 1.54184 Å, ω scans). The crystal was kept at 120(2) K during data collection.

S9.4.1 Specific crystal structure and refinement details for L[1]

Conformational disorder is modelled for both imidazole moieties, with respective pairs of rings rotated by approximately 180 degrees and substantially overlapping the atoms of the other component. In each case the occupancies of the components were refined and constrained to sum to unity giving values of 0.62(2) and 0.66(3) for the major components. The geometries of all four disordered imidazole moieties were restrained to be similar and planar (SAME, FLAT). The pairs of nitrogen atoms connecting to the phenyl rings were restrained to have identical positions. All pairs of overlapping disordered atoms were constrained to have identical atomic displacement parameters. Rigid bond and similarity restraints were applied to the anisotropic displacement parameters of all disordered atoms (RIGU, SIMU).

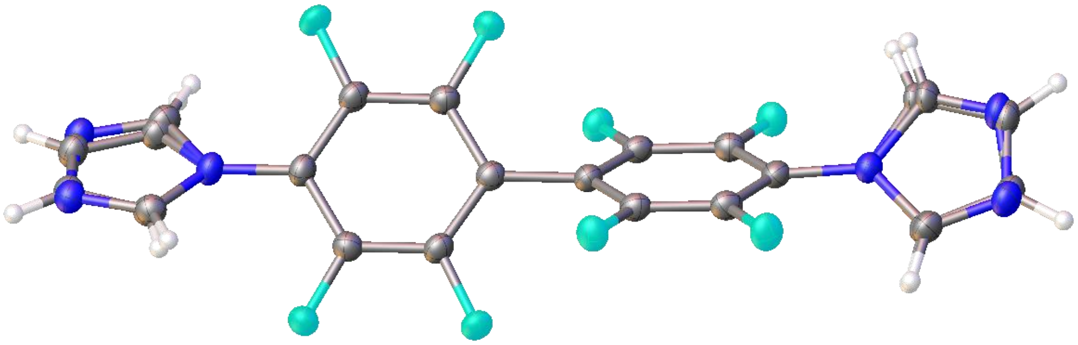

Figure S57: Image of **L[1]** at 50% ellipsoids

S9.5 Single crystal structure of ligand L^2

Single crystals of ligand **L**^{2} were grown via vapour diffusion of hexane into chloroform. A suitable crystal was selected and mounted using Fomblin (YR-1800 perfluoropolyether oil) on a XtalLAB PRO MM007, PILATUS3 R 200K diffractometer. The crystal was kept at 120(2) K during data collection.

S9.5.1 Specific crystal structure and refinement details for L^2

Conformational disorder is observed for all four imidazole moieties in the structure. The occupancies of the four pairs of disordered moieties were refined and respectively constrained to sum to unity, giving values of 0.63(1), 0.64(1), 0.71(1), and 0.71(1) for the major component in each case. The geometries of all the imidazole moieties were restrained to be similar (SAME, SADI). Rigid bond and similarity restraints were applied to the anisotropic displacement parameters of all atoms in the structure. All hydrogen atoms, including those of the minor disorder components, were observed in the electron density map before being geometrically placed and refined with a riding model.

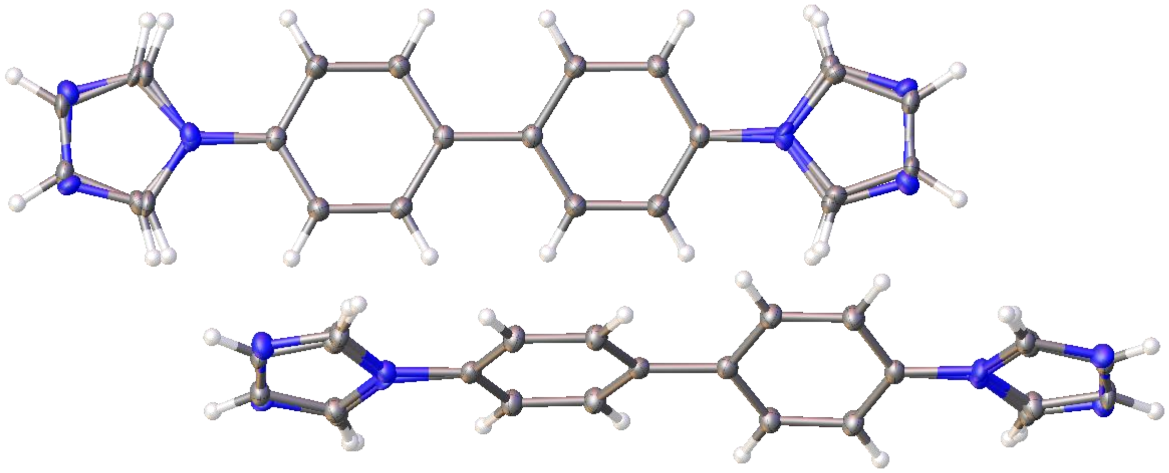

Figure S58: Image of **L**^{2} at 50% ellipsoids

S9.6 Single crystal structure of ligand L³

Single crystals of ligand **L³** were grown via vapour diffusion of hexane into chloroform. A suitable crystal was selected and mounted using Fomblin (YR-1800 perfluoropolyether oil) on an Oxford Diffraction GV1000 (TitanS2 CCD area detector, mirror-monochromated Cu-Kα radiation source; λ = 1.54184 Å, ω scans). The crystal was kept at 120(2) K during data collection.

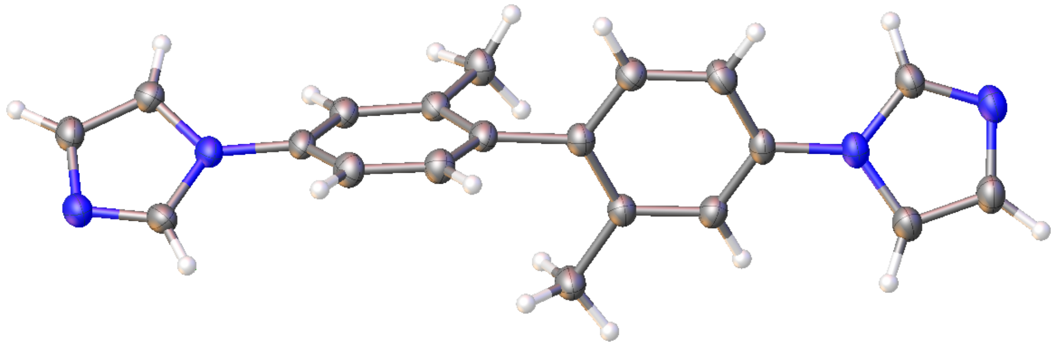

Figure S59: Image of **L³** at 50% ellipsoids

S9.7 Single crystal structure of ligand L^4

Single crystals of ligand $\mathbf{L^4}$ were grown via slow evaporation from a dichloromethane solution. A suitable crystal was selected and mounted using Fomblin (YR-1800 perfluoropolyether oil) on an Oxford Diffraction GV1000 (TitanS2 CCD area detector, mirror-monochromated Cu-Kα radiation source; λ = 1.54184 Å, ω scans). The crystal was kept at 120(2) K during data collection.

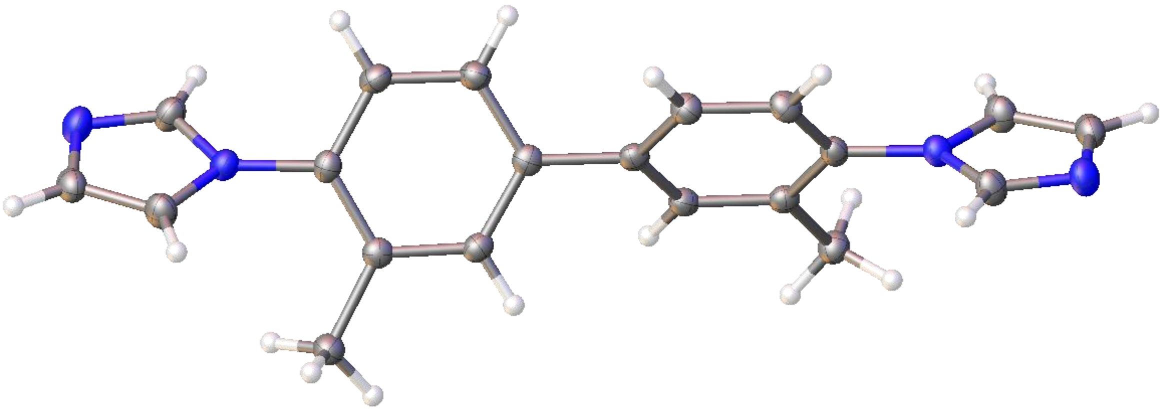

Figure S60: Image of $\mathbf{L^4}$ at 50% ellipsoids

S10 References

(1) Vlahakis, J. Z.; Mitu, S.; Roman, G.; Patricia Rodriguez, E.; Crandall, I. E.; Szarek, W. A. The anti-malarial activity of bivalent imidazolium salts. *Bioorg. Med. Chem.* **2011**, *19*, 6525 -6542.

(2) Belliston-Bittner, W.; Dunn, A. R.; Nguyen, Y. H. L.; Stuehr, D. J.; Winkler, J. R.; Gray, H. B. Picosecond Photoreduction of Inducible Nitric Oxide Synthase by Rhenium(I)−Diimine Wires. *J. Am. Chem. Soc.* **2005**, *127*, 15907 -15915.

(3) Hylland, K. T.; Øien-Ødegaard, S.; Lillerud, K. P.; Tilset, M. Efficient, Scalable Syntheses of Linker Molecules for Metal-Organic Frameworks. *Synlett* **2015**, *26*, 1480-1485.

(4) Martí-Centelles, V.; Piskorz, T. K.; Duarte, F. CageCavityCalc (C3): A Computational Tool for Calculating and Visualizing Cavities in Molecular Cages. *J. Chem. Inf. Model.* **2024**, *64*, 5604-5616.

(5) Cosier, J. t.; Glazer, A. A nitrogen-gas-stream cryostat for general X-ray diffraction studies. *J. Appl. Crystallogr.* **1986**, *19*, 105 -107.

(6) Rigaku Oxford Diffraction, (2018), CrysAlisPro Software system, version 1.171.40.45a, Rigaku Corporation, Oxford, UK.

(7) Allan, D. R.; Nowell, H.; Barnett, S. A.; Warren, M. R.; Wilcox, A.; Christensen, J.; Saunders, L. K.; Peach, A.; Hooper, M. T.; Zaja, L. A novel dual air-bearing fixed-χ diffractometer for small-molecule single-crystal X-ray diffraction on beamline I19 at diamond light source. *Crystals* **2017**, *7*, 336.

(8) Winter, G. xia2: an expert system for macromolecular crystallography data reduction. *J. Appl. Crystallogr.* **2010**, *43*, 186 -190.

(9) Dolomanov, O. V.; Bourhis, L. J.; Gildea, R. J.; Howard, J. A.; Puschmann, H. OLEX2: a complete structure solution, refinement and analysis program. *J. Appl. Crystallogr.* **2009**, *42*, 339 -341.

(10) Sheldrick, G. SHELXT - Integrated space-group and crystal-structure determination. *Acta Cryst. A* **2015**, *71*, 3 -8.

(11) Sheldrick, G. Crystal structure refinement with SHELXL. *Acta Cryst. C* **2015**, *71*, 3 -8.

(12) “CheckCIF,” can be found under http://checkcif.iucr.org.
